# Remarkable effect of alkynyl substituents on the fluorescence properties of a BN-phenanthrene

**DOI:** 10.3762/bjoc.15.122

**Published:** 2019-06-06

**Authors:** Alberto Abengózar, David Sucunza, Patricia García-García, Juan J Vaquero

**Affiliations:** 1 Departamento de Química Orgánica y Química Inorgánica, Instituto de Investigación Química “Andrés M. del Río” (IQAR), Universidad de Alcalá, 28871-Alcalá de Henares, Madrid, Spain

**Keywords:** alkyne, BN-phenanthrene, cross-coupling, fluorescence, heterocycles

## Abstract

A series of BN-phenanthrenes with substituents of a diverse nature have been synthesized by palladium-catalyzed cross-coupling reactions of a common chloro-substituted precursor, which was made from readily available materials in only four steps. Evaluation of the photophysical properties of the prepared compounds unveiled an impressive effect of the presence of alkynyl substituents on the fluorescence quantum yield, which improved from 0.01 in the parent compound to up to 0.65 in derivatives containing a triple bond.

## Introduction

BN-polycyclic aromatic hydrocarbons (BN-PAHs) have received increasing interest over the past few years [1–5], particularly in the field of materials science [6]. The presence of a polarized B–N bond induces significant changes in the photophysical properties of these compounds when compared to their PAH analogues containing only non-polar C=C bonds. This fact opens up new opportunities for creating improved optoelectronic devices [7–15].

The introduction of substituents is known to have a substantial influence on the photophysical properties of PAHs. However, although some particular examples of the impact of substituents on the behaviour of BN-PAHs have been reported [16–21], systematic studies are not usually performed, probably due to the difficulties associated with their synthesis and the lack of general methods for their functionalization [22].

**Figure 1:**
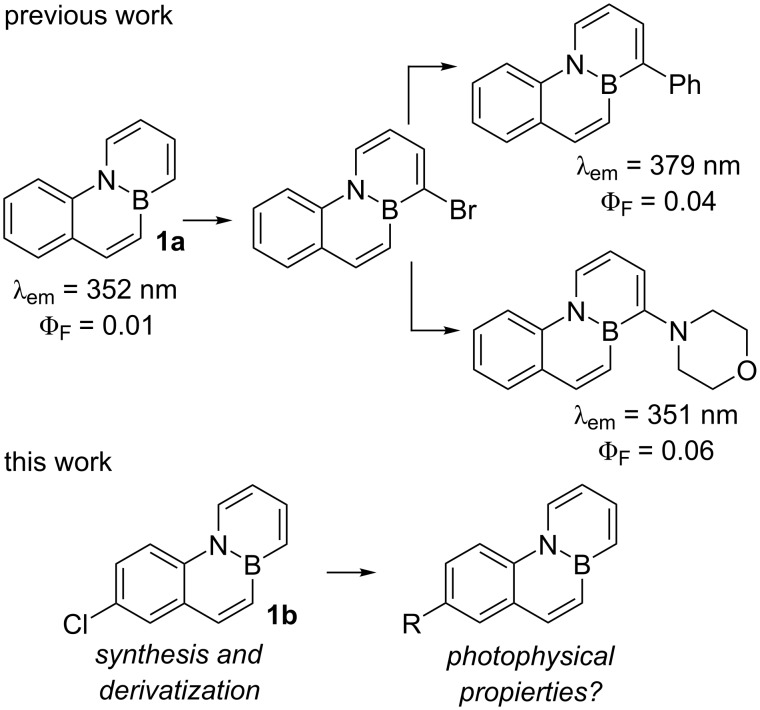
BN-phenanthrene **1a** and synthesis of substituted derivatives proposed in this work.

We have recently designed an efficient synthesis for one of the simplest BN-PAHs, namely BN-phenanthrene **1a** [23]. We are interested in evaluating the reactivity [24] and properties of **1a** in greater detail as this could provide valuable information that leads to a better understanding of the behaviour of BN-aromatics. Interestingly, **1a** turned out to be weakly fluorescent [23], in contrast to other BN-phenanthrene isomers described previously [25–26]. The presence of aryl or amino substituents at C1, which can be introduced via bromination and subsequent palladium-catalyzed cross coupling, does not have a significant impact on the fluorescence of these compounds (Figure 1) [23]. In order to gain a deeper understanding of the photophysical properties of the BN-phenanthrene core, we decided to evaluate the influence of substituents located in other positions. We were particularly interested in the effect of alkynyl substituents, as their presence in PAHs is known to alter the fluorescence properties thereof markedly [27–29]. In this regard, we have recently described a methodology for the synthesis of a chloro-substituted BN-benzo[*c*]phenanthrene and its subsequent derivatization via palladium-catalyzed cross-coupling reactions [30], and we envisioned that this reaction could be used to prepare C7 substituted BN-phenanthrenes (Figure 1).

Herein we report the synthesis of chloro-substituted BN-phenanthrene **1b**, its derivatization via palladium-catalyzed cross-coupling reactions and the significant effect of the substituents on the fluorescence properties of the compounds prepared.

## Results and Discussion

**Scheme 1:**
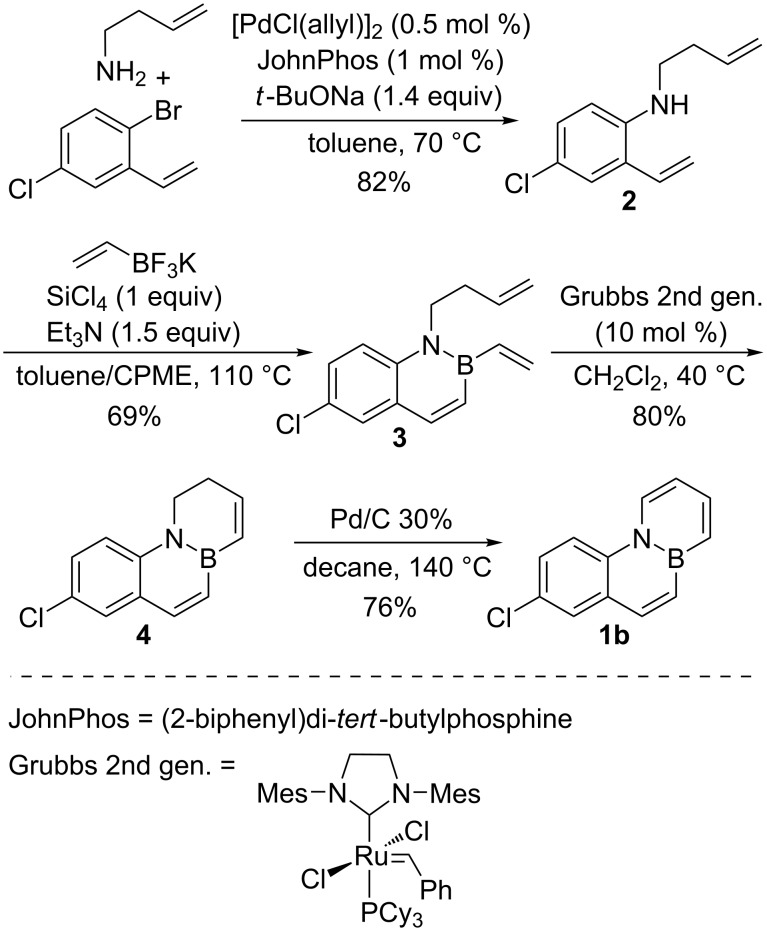
Synthesis of Cl-substituted BN-phenanthrene **1b**.

Our first aim was to synthesise the Cl-substituted BN-phenanthrene **1b** (Scheme 1), following a synthetic sequence analogous to that described previously by us for preparation of the parent BN-phenanthrene **1a** [23]. Thus, an initial Buchwald–Hartwig amination between 2-bromo-5-chlorostyrene and 3-butenylamine was the initial step. This coupling was performed at 70 °C, as a higher yield was obtained at this temperature (71% at 80 °C, 24 h vs 82% at 70 °C, 48 h). Substrate **2** was then cyclized with vinyl trifluoroborate. The optimal conditions to obtain **3** were found to be heating at 110 °C for 72 h. Lower temperatures and/or shorter times led to incomplete conversions in the borylative cyclization of **2**. Ring-closing metathesis of **3** proceeded efficiently in the presence of 10 mol % of the second-generation Grubbs catalyst to yield dihydro-BN-phenanthrene **4**, which was oxidized to **1b** under the conditions previously optimized for the synthesis of the parent BN-phenanthrene **1a** [23].

**Scheme 2:**
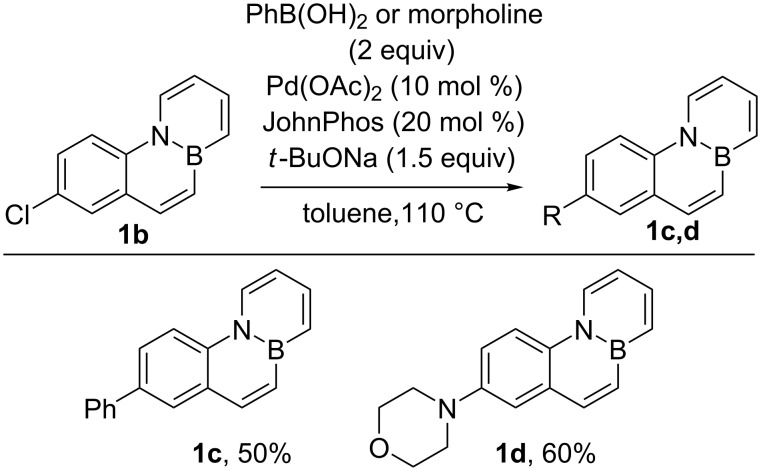
Palladium-catalyzed cross-couplings of Cl-substituted BN-phenanthrene **1b**.

Next, we explored the preparation of various substituted BN-phenanthrenes by means of palladium-catalyzed cross-coupling reactions of **1b**, under conditions optimized for a related BN-benzo[*c*]phenanthrene [30]. Gratifyingly, Suzuki coupling and Buchwald–Hartwig amination yielded the corresponding aryl- and amino-substituted BN-phenanthrenes **1c** and **1d** in good yields (Scheme 2).

**Scheme 3:**
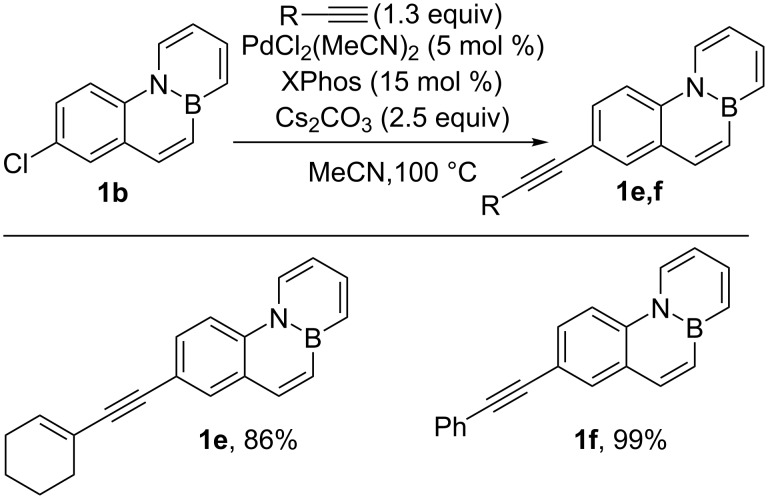
Pd-catalyzed Sonogashira reactions of Cl-substituted BN-phenanthrene **1b**.

Moreover, Sonogashira couplings efficiently proceed to provide alkynyl-substituted BN-phenanthrenes **1e** and **1f** in excellent yields (Scheme 3). These results confirm the value of palladium-catalyzed cross-coupling reactions of chloro-substituted BN-arenes as a useful tool for the preparation of derivatives functionalized with a range of substituents of a different nature.

**Figure 2:**
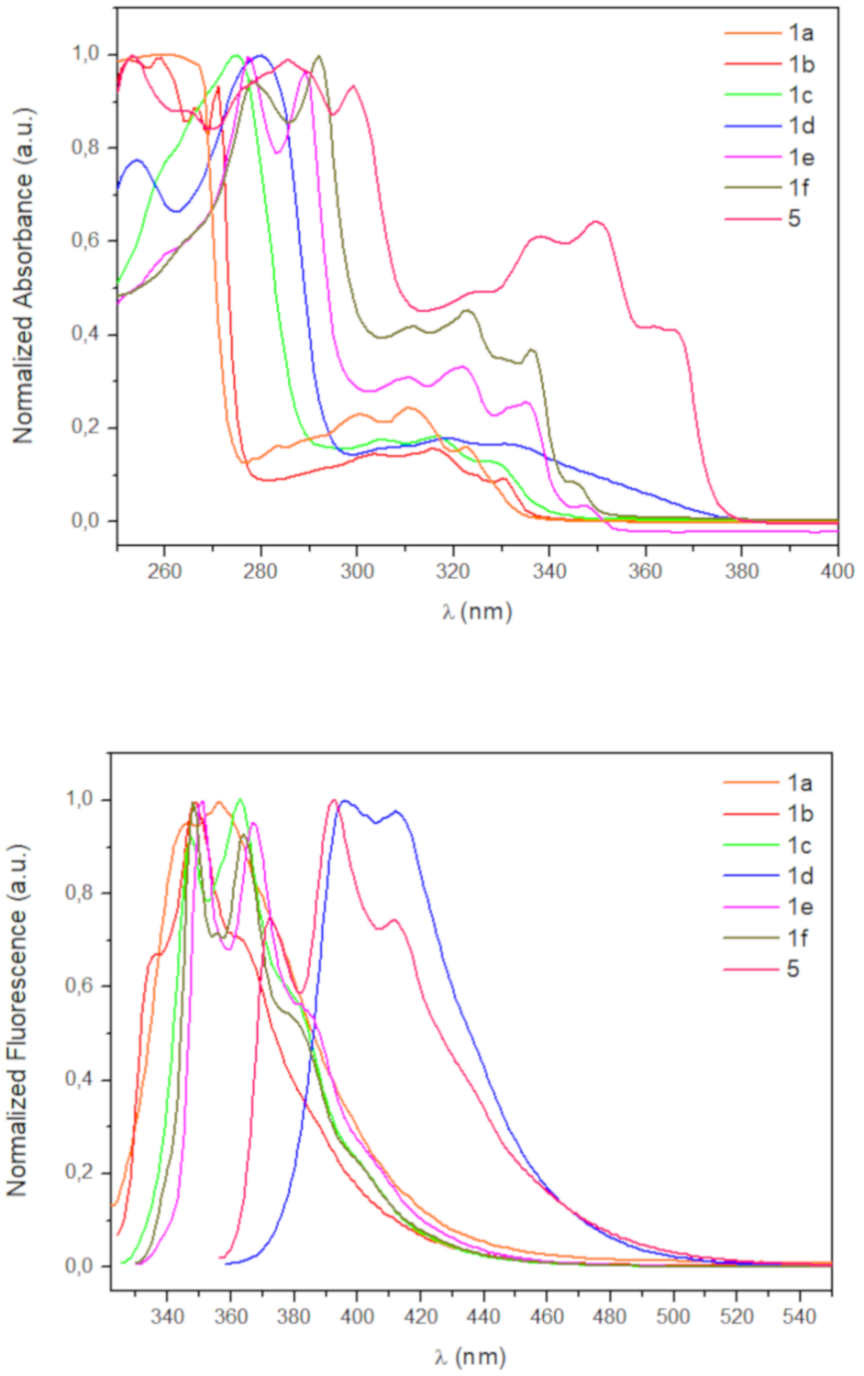
UV–vis absorption (top) and emission (bottom) spectra for BN-phenanthrenes **1** and **5** in cyclohexane (≈0.02 mM).

**Figure 3:**
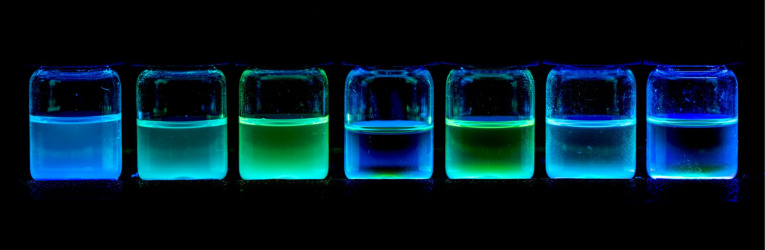
Solutions of **1a**–**f** and **5** (from left to right) under UV irradiation.

Once we had developed a useful method for the synthesis of the functionalized BN-phenanthrenes **1**, we focussed on our initial goal of evaluating the influence of different substituents on the photophysical properties thereof. The absorption and emission spectra of the parent BN-phenanthrene **1a** and the derivatives prepared in this work are shown in Figure 2. 1-(Phenylethynyl)-4a-aza-10a-boraphenanthrene (**5**), which was previously prepared in our group by bromination of **1a** and subsequent coupling [23], was also included in this comparative study. The absorption and emission data for these compounds are summarized in Table 1, and a picture of their solutions under UV irradiation are shown in Figure 3.

**Table 1:** UV–vis and fluorescence data for BN-phenanthrenes **1a**–**f** and **5**.^a^

Compound	ε (M^−1^cm^−1^)	λ_abs max_ (nm)	λ_em_ (nm)	Φ_f_^b^

**1a**	5488	310	356	0.01
**1b**	7715	316	349	0.03
**1c**	7482	316	363	0.17
**1d**	4331	319	395	0.19
**1e**	18283	322	351	0.44
**1f**	19392	323	348	0.65
**5**	15851	350	392	0.45

^a^All experiments were performed in cyclohexane solution (≈0.01–0.02 mM). The excitation wavelength match the absorption maxima for each compound. ^b^Quantum yields reported relative to 9,10-diphenylanthracene (Φ_f_ = 0.93).

The emission maxima were not significantly affected by the presence of substituents, except for 7-amino substituted BN-phenanthrene **1d** and 1-alkynyl substituted BN-phenanthrene **5**, whose emission maxima are slightly red-shifted (395 and 392 nm vs 356 nm for unsubstituted **1a**). In contrast, 7-alkynyl-substituted BN-phenanthrenes **1e** and **1f** show emission maxima analogous to that of the parent BN-phenanthrene **1a**. With regard to the fluorescence quantum yield, phenyl and morpholine substituents at C7 (**1c** and **1d**) provide a significant increase when compared to the parent compound (0.17 and 0.19 vs 0.01). This enhancement is higher than that observed when these same substituents are located at C1 (see Figure 1) [23]. More interestingly, the presence of alkynyl substituents at C7 gives rise to a marked increase in the fluorescence quantum yield (0.44 and 0.65), particularly when the triple bond is bonded to a phenyl ring (**1f**). A similar increase (Φ_f_ = 0.45) is observed when the alkynyl group is attached to C1 (**5**), thus indicating that the positive influence of the triple bond on the fluorescence quantum yield of BN-phenanthrene seems to be a general effect, irrespective of its position. It should be noted that the introduction of ethynyl groups into the all-carbon phenanthrene skeleton results in a slight increase in the fluorescence quantum yields compared to that of phenanthrene [27]. However, the effect observed here for BN-phenanthrenes is much more pronounced [31]. We have also evaluated the emission of **1f** in different solvents. The emission maxima are almost unaffected by the nature of the solvent [32], whereas the fluorescence quantum yield decreases in more polar solvents (0.29 in CH_2_Cl_2_, 0.19 in THF, 0.39 in 1,4-dioxane, 0.26 in MeCN)

## Conclusion

We have successfully prepared a chloro-substituted BN-phenanthrene derivative that serves as a useful intermediate for the synthesis of a range of BN-phenanthrenes substituted with groups of diverse nature at C-7. This efficient post-functionalization methodology allows the influence of substituents on the photophysical properties of the BN-phenanthrene core to be studied. Substituted derivatives show an improved quantum yield with respect to the parent BN-phenanthrene, an effect that is particularly noteworthy for alkynyl substituents. A BN-phenanthrene bearing the triple bond at C-1 shows a similar increase in the quantum yield, thus suggesting that the influence of the alkynyl substituent is not limited to a particular position. We consider that this discovery may have important implications in the discovery of novel BN-arenes with improved properties. Further studies into the effect of alkynyl substituents on the fluorescence of BN-arenes are currently ongoing in our laboratories.

## Supporting Information

File 1 Experimental details and NMR spectra for all new compounds.
