# Supplementary material for: Remarkable effect of alkynyl substituents on the fluorescence properties of a BN-phenanthrene
Source: Beilstein J Org Chem. 2019 Jun 6;15:1257–61. doi: 10.3762/bjoc.15.122 (PMC6604742; doi:10.3762/bjoc.15.122)

BEILSTEIN JOURNAL OF ORGANIC CHEMISTRY

Supporting Information

for

Remarkable effect of alkynyl substituents on the fluorescence properties of a BN-phenanthrene

Alberto Abengózar, David Sucunza, Patricia García-García and Juan J. Vaquero

Beilstein J. Org. Chem. **2019,** *15,* 1257–1261. doi:10.3762/bjoc.15.122

Experimental details and NMR spectra for all new compounds

License and Terms: This is a supporting information file under the terms of the Creative Commons Attribution License (http://creativecommons.org/licenses/by/4.0). Please note that the reuse, redistribution and reproduction in particular requires that the authors and source are credited.
The license is subject to the *Beilstein Journal of Organic Chemistry* terms and conditions: (https://www.beilstein-journals.org/bjoc)

Table of contents

General experimental details ---S2

Experimental procedures and data ---S3

Synthesis of 7-chloro-4a-aza-10a-boraphenanthrene (**1b**) -------------------------------------S3

Palladium-catalyzed cross coupling reactions of 7-chloro-4a-aza-10a-boraphenanthrene (**1b**)--S6

UV–vis and fluorescence data for **1f** in different solvents--- S11

Copies of ^{1}H, ^{13}C and ^{11}B NMR spectra for novel compounds and selected gCOSY, TOCSY, gHSQC and gHMBC spectra ---S12

GENERAL EXPERIMENTAL DETAILS

Reagents were acquired from commercial sources and were used without further purification. Dry solvents, where necessary, were dried using an MBRAUN MB-SPS-800 apparatus. In general, reactions were carried out under an argon atmosphere using oven-dried glassware with magnetic stirring and dry solvents. Reactions were monitored by using analytical TLC plates (Merck; silica gel 60 F254, 0.25 mm), and compounds were visualized with UV radiation. Silica gel grade 60 (70-230 mesh, Merck) was used for column chromatography. All melting points were determined in open capillary tubes using a Stuart Scientific SMP3 melting point apparatus. IR spectra were obtained using a Perkin–Elmer FTIR spectrum 2000 spectrophotometer. ^{1}H, ^{13}C and ^{11}B NMR spectra were recorded using either a Varian Mercury VX-300, Varian Unity 300 or Varian Unity 500 MHz spectrometer at room temperature. Chemical shifts are given in ppm (δ) downfield from TMS, with calibration on the residual protio-solvent used (δ_H = 7.24 ppm and δ_C = 77.0 ppm for $CDCl_3$). Coupling constants (*J*) are in Hertz (Hz) and signals are described as follows: s, singlet; d, doublet; t, triplet; q, quadruplet; m, multiplet; b, broad; ap, apparent. High-resolution analysis (HRMS) were performed using an Agilent 6210 time of-flight LC/MS. Elemental analysis was performed using a LECO CHNSO-932 instrument.

Absorption spectra were recorded using a UV–vis Uvikon 941 (Kontron Instruments) spectrophotometer. Steady-state fluorescence measurements were carried out using a PTI Quanta Master spectrofluorimeter equipped with a Xenon flash lamp as light source, single concave grating monochromators and Glan-Thompson polarizers in the excitation and emission paths. Detection was allowed by a photomultiplier cooled by a Peltier system. Slit widths were selected at 6 nm for both excitation and emission paths and polarizers were fixed at the "magic angle" condition. Right angle geometry and rectangular 10 mm path cells were used for the fluorescence measurements. Fluorescence quamtum yields were determined using as the standard 9,10-diphenylanthracene in solution of cyclohexane[1]

The starting material 1-bromo-4-chloro-2-vinylbenzene was prepared according to published procedures.[2]

---

[1] (a) A. M. Brouwer, *Pure Appl. Chem.*, **2011**, *83*, 2213; (b) F. Mendicuti, W. L. Mattice, *Polym. Bull.*, **1989**, *22*, 557.

[2] A. Abengózar, P. García-García, D. Sucunza, A. Pérez-Redondo, J. J. Vaquero, *Chem. Commun.*, **2018**, *54*, 2467.

EXPERIMENTAL PROCEDURES AND DATA

Synthesis of 7-chloro-4a-aza-10a-boraphenanthrene (1b)

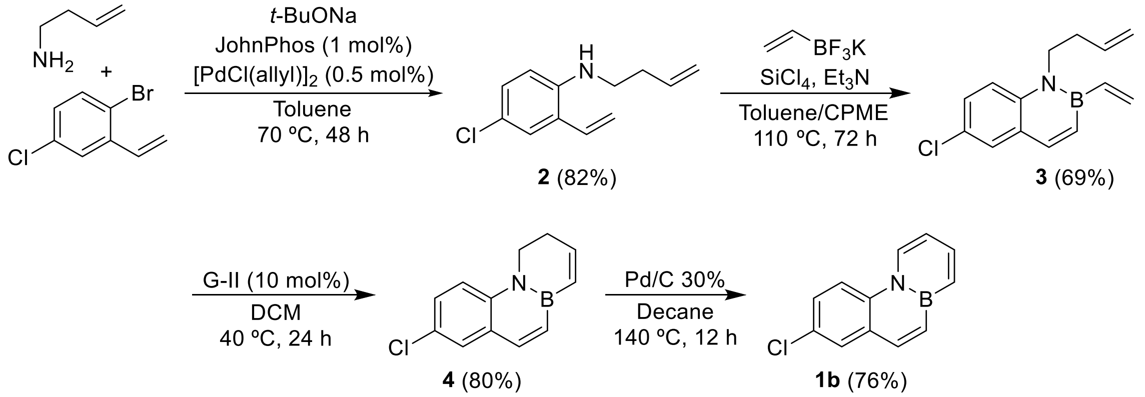

Preparation of *N*-(but-3-en-1-yl)-4-chloro-2-vinylaniline (2).

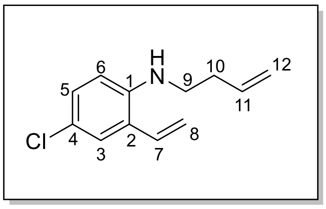

[PdCl(allyl)]$_2$ (6.0 mg, 0.017 mmol, 0.5 mol %), JohnPhos (10 mg, 0.035 mmol, 1.0 mol%), and *t*-BuONa (481 mg, 4.86 mmol, 1.4 equiv) were added to an oven-dried vial equipped with a stir bar. The vial was sealed with a cap lined with a disposable Teflon septum, evacuated under vacuum, and purged with argon three times. Toluene (6 mL) was then added, followed by 1-bromo-4-chloro-2-vinylbenzene (755 mg, 3.47 mmol, 1.0 equiv) and 3-butenylamine (410 μL, 4.17 mmol, 1.2 equiv). The reaction mixture was heated to 70 °C for 48 h, then the crude mixture was cooled to room temperature, diluted with Et$_2$O (10 mL) and filtered through a pad of Celite®. The solvent was removed under reduced pressure and the remaining oil was purified by flash column chromatography (1% EtOAc/Hexane) to give **2** (588 mg, 2.83 mmol, 82%) as a yellow oil.

IR (NaCl) $\tilde{\upsilon}_{max}$ (cm^{-1}) 3423 (NH), 3081, 2922, 2850, 1597, 1500, 1419, 1319, 993, 917, 805.

^{1}H-NMR (300 MHz, CDCl$_3$) δ (ppm) 7.20 (d, *J* = 2.5 Hz, 1H, H-3), 7.10 (dd, *J* = 8.7, 2.5 Hz, 1H, H-5), 6.63 (dd, J_{trans} = 17.3 Hz, J_{cis} = 11.0 Hz, 1H, H-7), 6.54 (d, *J* = 8.7 Hz, 1H, H-6), 5.82 (ddt, J_{trans} = 17.1 Hz, J_{cis} = 10.2 Hz, *J* = 6.7 Hz, 1H, H-11), 5.59 (dd, J_{trans} = 17.3 Hz, J_{gem} = 1.4 Hz, 1H, H-8), 5.33 (dd, J_{cis} = 11.0 Hz, J_{gem} = 1.4 Hz, 1H, H-8), 5.16 (ap dq, J_{trans} = 17.1 Hz, *J* = 1.4 Hz, 1H, H-12), 5.14–5.10 (m, 1H, H-12), 3.85 (br s, 1H, N*H*), 3.16 (t, *J* = 6.7 Hz, 2H, H-9), 2.40 (ap qt, *J* = 6.7, 1.4 Hz, 2H, H-10).

^{13}C-NMR (75 MHz, CDCl$_3$) δ (ppm) 143.7 (C-1), 135.5 (C-11), 131.6 (C-7), 128.4 (C-5), 127.0 (C-3), 125.6 (C-4), 121.9 (C-2), 117.4 (C-6), 117.3 (C-12), 111.9 (C-8), 42.8 (C-9), 33.4 (C-10).

HRMS (APCI) *m/z* calculated for $C_{12}H_{15}ClN$ [M+H]$^+$: 208.0888; found [M+H]$^+$: 208.0886.

Preparation of 1-(but-3-en-1-yl)-6-chloro-2-vinyl-1-aza-2-boranaphthalene (3).

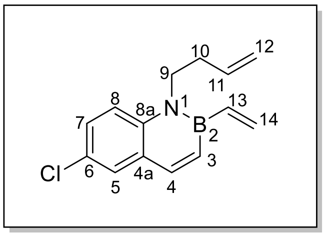

Potassium vinyltrifluoroborate (328 mg, 2.33 mmol, 1.0 equiv) was added to an oven-dried vial equipped with a stir bar. The vial was sealed with a cap lined with a disposable Teflon septum, evacuated under vacuum, and purged with argon three times. CMPE (5 mL) and toluene (5 mL) were then added, followed by amine **2** (580 mg, 2.79 mmol, 1.2 equiv), $SiCl_4$ (270 μL, 2.33 mmol, 1.0 equiv) and Et_3N (487 μL, 3.50 mmol, 1.5 equiv) under argon. The reaction mixture was heated to 110 °C for 72 h, then the crude mixture was cooled to room temperature, diluted with Et_2O (10 mL) and filtered through a pad of silica gel. The solvent was removed under reduced pressure and the remaining residue was purified by flash column chromatography (hexane) to give **3** (394 mg, 1.62 mmol, 69%) as a light-yellow oil.

IR (NaCl) $\tilde{\upsilon}_{max}$ (cm^{-1}) 3061, 2924, 2853, 1605, 1544, 1477, 1415, 1387, 1283, 1209, 1001, 952, 918, 884, 799.

^{1}H-NMR (500 MHz, $CDCl_3$) δ (ppm) 7.83 (d, *J* = 11.5 Hz, 1H, H-4), 7.58 (d, *J* = 2.1 Hz, 1H, H-5), 7.44 (d, *J* = 9.1 Hz, 1H, H-8), 7.40 (dd, *J* = 9.1, 2.1 Hz, 1H, H-7), 7.06 (d, *J* = 11.5 Hz, 1H, H-3), 6.69 (dd, J_{trans} = 19.4 Hz, J_{cis} = 13.5 Hz, 1H, H-13), 6.22 (dd, J_{trans} = 19.4 Hz, J_{gem} = 3.7 Hz, 1H, H-14), 6.09 (dd, J_{cis} = 13.5 Hz, J_{gem} = 3.7 Hz, 1H, H-14), 5.87 (ddt, J_{trans} = 17.1 Hz, J_{cis} = 10.2 Hz, *J* = 6.8 Hz, 1H, H-11), 5.11 (ap dq, J_{trans} = 17.1 Hz, *J* = 1.6 Hz, 1H, H-12), 5.11–5.06 (m, 1H, H-12), 4.20–4.14 (m, 2H, H-9), 2.49 (ap qt, *J* = 6.8, 1.6 Hz, 2H, H-10).

^{13}C-NMR (125 MHz, $CDCl_3$) δ (ppm) 143.4 (C-4), 139.4 (C-8a), 137.6 (C-13*), 134.6 (C-11), 133.8 (C-14), 129.8 (C-3*), 129.3 (C-5), 128.3 (C-7), 128.2 (C-4a), 125.7 (C-6), 117.0 (C-12), 116.2 (C-8), 46.3 (C-9), 34.2 (C-10). *Carbon not observed in ^{13}C-NMR, assigned by gHSQC.

^{11}B-NMR (160 MHz, $CDCl_3$) δ (ppm) 33.83.

HRMS (EI) *m/z* calculated for $C_{14}H_{15}BClN$ $[M]^+$: 243.0981; found $[M]^+$: 243.0981.

Preparation of 7-chloro-3,4-dihydro-4a-aza-10a-boraphenanthrene (4).

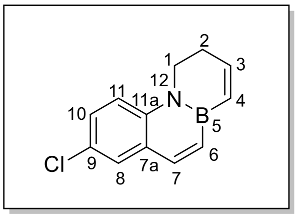

Grubbs' second-generation ruthenium catalyst (136 mg, 0.160 mmol, 10 mol %) in dichloromethane (3 mL) was added to a solution of diene **3** (390 mg, 1.60 mmol, 1.0 equiv) in dichloromethane (16 mL) under argon and the reaction mixture was heated to reflux for 24 h. The crude mixture was then cooled to room temperature,

diluted with dichloromethane (20 mL) and filtered through a pad of silica gel. The solvent was removed under reduced pressure and the remaining residue was purified by flash column chromatography (hexane) to give **4** (277 mg, 1.29 mmol, 80%) as a colourless oil.

IR (NaCl) $\tilde{\upsilon}_{max}$ (cm^{-1}) 3010, 2927, 1606, 1587, 1542, 1392, 1316, 1203, 1171, 882, 801.

^{1}H-NMR (500 MHz, $CDCl_3$) δ (ppm) 7.83 (d, *J* = 11.3 Hz, 1H, H-7), 7.56 (d, *J* = 2.5 Hz, 1H, H-8), 7.47 (d, *J* = 9.1 Hz, 1H, H-11), 7.40 (dd, *J* = 9.1, 2.5 Hz, 1H, H-10), 6.85 (d, *J* = 11.3 Hz, 1H, H-6), 6.76 (dt, *J* = 11.7, 4.1 Hz, 1H, H-3), 6.30 (dt, *J* = 11.7, 1.6 Hz, 1H, H-4), 3.98 (t, *J* = 7.3 Hz, 2H, H-1), 2.56 (tdd, *J* = 7.3, 4.1, 1.6 Hz, 2H, H-2).

^{13}C-NMR (125 MHz, $CDCl_3$) δ (ppm) 144.0 (C-7), 142.7 (C-3), 140.3 (C-11a), 130.3 (C-6*), 129.4 (C-4*), 129.2 (C-8), 128.2 (C-10), 127.9 (C-7a), 125.1 (C-9), 114.9 (C-11), 42.4 (C-1), 28.2 (C-2). *Carbon not observed in ^{13}C-NMR, assigned by gHSQC.

^{11}B-NMR (160 MHz, $CDCl_3$) δ (ppm) 30.93.

MS (DIP-IE) *m/z* (relative intensity, %): 215 (M^+, 100).

Elemental analysis calculated for $C_{12}H_{11}BClN$ (215.49 g/mol): C, 66.89; H, 5.15; N, 6.50. Found: C, 67.28; H, 5.13; N, 6.35.

Preparation of 7-chloro-4a-aza-10a-boraphenanthrene (1b).

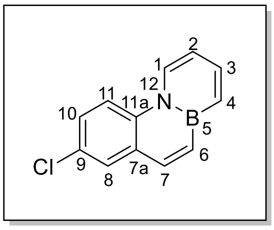

Pd/C 30% (100 mg, 40% w/w) was added to a solution of BN-phenanthrene derivative **4** (250 mg, 1.16 mmol, 1.0 equiv.) in decane (14 mL) and the reaction mixture heated to 140 °C for 12 h. The crude mixture was then cooled to room temperature, diluted with dichloromethane (10 mL) and filtered through a pad of Celite®. The solvent was removed under reduced pressure and the remaining residue was purified by flash column chromatography (Hexane) to give **1b** (187 mg, 0.876 mmol, 76%) as a light-yellow oil.

IR (NaCl) $\tilde{\upsilon}_{max}$ (cm^{-1}) 3014, 2926, 2861, 1616, 1595, 1468, 1395, 1276, 881, 791, 729.

^{1}H-NMR (500 MHz, $CDCl_3$) δ (ppm) 8.56 (d, *J* = 7.5 Hz, 1H, H-1), 8.10 (d, *J* = 9.2 Hz, 1H, H-11), 7.86 (d, *J* = 11.5 Hz, 1H, H-7), 7.77 (dd, *J* = 10.9, 6.2 Hz, 1H, H-3), 7.74 (d, *J* = 2.5 Hz, 1H, H-8), 7.52 (dd, *J* = 9.2, 2.5 Hz, 1H, H-10), 7.38 (dd, *J* = 10.9, 1.9 Hz, 1H, H-4), 7.36 (d, *J* = 11.5 Hz, 1H, H-6), 6.78 (ddd, *J* = 7.5, 6.2, 1.9 Hz, 1H, H-2).

^{13}C-NMR (125 MHz, $CDCl_3$) δ (ppm) 140.5 (C-3), 139.6 (C-7), 136.2 (C-11a), 132.2 (C-6*), 132.0 (C-4*), 129.8 (C-8), 129.4 (C-7a), 129.0 (C-9), 128.0 (C-1), 127.9 (C-10), 116.1 (C-11), 114.2 (C-2). *Carbon not observed in ^{13}C-NMR, assigned by gHSQC.

^{11}B-NMR (160 MHz, $CDCl_3$) δ (ppm) 28.86.

HRMS (EI) *m/z* calculated for $C_{12}H_9BClN$ $[M]^+$: 213.0511; found $[M]^+$: 213.0516.

Palladium-Catalyzed cross coupling reactions of 7-chloro-4a-aza-10a-boraphenanthrene (1b)

Suzuki and Buchwald-Hartwig Reactions

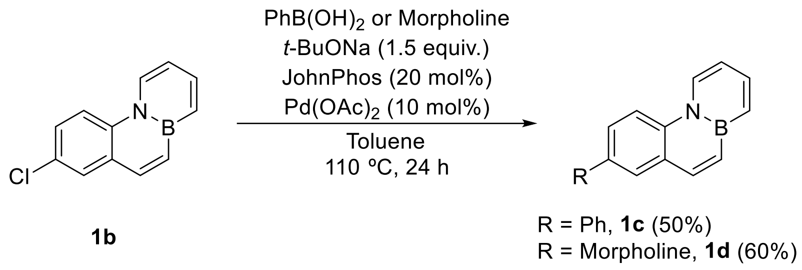

Preparation of 7-phenyl-4a-aza-10a-boraphenanthrene (1c).

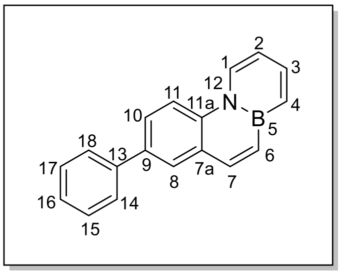

$Pd(OAc)_2$ (4.3 mg, 0.019 mmol, 10 mol%), JohnPhos (12 mg, 0.038 mmol, 20 mol%), *t*-BuONa (28 mg, 0.29 mmol, 1.5 equiv), phenylboronic acid (49 mg, 0.38 mmol, 2.0 equiv.) and BN-phenanthrene **1b** (40 mg, 0.19 mmol, 1.0 equiv.) were added to an oven-dried vial equipped with a stir bar. The vial was sealed with a cap lined with a disposable Teflon septum, evacuated and purged with argon three times. Toluene (2 mL) was then added and the reaction mixture heated to 110 °C for 24 h. The crude mixture was subsequently cooled to room temperature, and diluted with CH_2Cl_2 (10 mL) and water (10 mL). The layers were then separated and the aqueous layer extracted with CH_2Cl_2 (2 x 10 mL). The combined organic layers were dried over anhydrous Na_2SO_4, filtered and concentrated under reduced pressure. The remaining residue was purified by flash column chromatography (Hexane) to give **1c** (24 mg, 0.095 mmol, 50%) as a white solid.

M. p.: 124–126 °C.

IR (KBr) $\tilde{\upsilon}_{max}$ (cm^{-1}) 3025, 1614, 1594, 1518, 1455, 1394, 1265, 895, 801, 765, 734.

^{1}H-NMR (500 MHz, $CDCl_3$) δ (ppm) 8.69 (d, *J* = 7.4 Hz, 1H, H-1), 8.27 (d, *J* = 8.9 Hz, 1H, H-11), 8.04 (d, *J* = 11.4 Hz, 1H, H-7), 8.00 (d, *J* = 2.3 Hz, 1H, H-8), 7.84 (dd, *J* = 8.9, 2.3 Hz, 1H, H-10), 7.78

(ddd, J = 11.0, 6.3, 0.8 Hz, 1H, H-3), 7.72–7.70 (m, 2H, H-14, H-18), 7.50–7.47 (m, 2H, H-15, H-17), 7.41–7.36 (m, 2H, H-4, H-16), 7.36 (d, J = 11.4 Hz, 1H, H-6), 6.80 (ddd, J = 7.4, 6.3, 1.7 Hz, 1H, H-2).

^{13}C-NMR (125 MHz, CDCl$_3$) δ (ppm) 140.9 (C-7), 140.3 (C-3), 140.2 (C-13), 137.1 (C-11a), 136.4 (C-9), 132.0 (C-4*), 131.5 (C-6*), 129.0 (C-8), 128.9 (2C, C-15, C-17), 128.5 (C-7a), 128.2 (C-1), 127.3 (C-16), 127.1 (2C, C-14, C-18), 126.8 (C-10), 115.1 (C-11), 113.9 (C-2). *Carbon not observed in ^{13}C-NMR, assigned by gHSQC.

^{11}B-NMR (160 MHz, CDCl$_3$) δ (ppm) 28.82.

HRMS (EI) *m/z* calculated for $C_{18}H_{14}BN$ [M]$^+$: 255.1214; found [M]$^+$: 255.1218.

Preparation of 7-morpholino-4a-aza-10a-boraphenanthrene (1d).

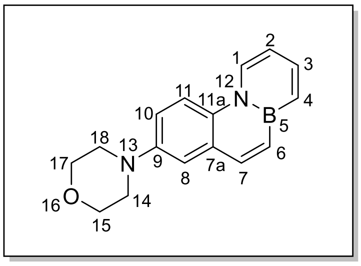

Pd(OAc)$_2$ (4.3 mg, 0.019 mmol, 10 mol%), JohnPhos (12 mg, 0.038 mmol, 20 mol%), *t*-BuONa (28 mg, 0.29 mmol, 1.5 equiv) and BN-phenanthrene **1b** (40 mg, 0.19 mmol, 1.0 equiv) were added to an oven-dried vial equipped with a stir bar. The vial was sealed with a cap lined with a disposable Teflon septum, evacuated under vacuum, and purged with argon three times. Toluene (2 mL) was then added, followed by morpholine (34 μL, 0.38 mmol, 2.0 equiv), and the reaction mixture was heated to 110 °C for 24 h. The crude mixture was subsequently cooled to room temperature, and diluted with CH_2Cl_2 (10 mL) and water (10 mL). The layers were separated, and the aqueous layer was extracted with CH_2Cl_2 (2 x 10 mL). The combined organic layers were dried over anhydrous Na_2SO_4, filtered and concentrated under reduced pressure. The remaining residue was purified by flash column chromatography (20% EtOAc/hexane) to give **1d** (30 mg, 0.11 mmol, 60%) as a white solid.

M. p.: 124–126 °C.

IR (KBr) $\tilde{\upsilon}_{max}$ (cm^{-1}) 2958, 2852, 1612, 1558, 1516, 1451, 1266, 1123, 973, 792.

^{1}H-NMR (500 MHz, CDCl$_3$) δ (ppm) 8.61 (d, J = 7.4 Hz, 1H, H-1), 8.10 (d, J = 9.3 Hz, 1H, H-11), 7.89 (d, J = 11.4 Hz, 1H, H-7), 7.74 (ddd, J = 10.9, 6.3, 0.7 Hz, 1H, H-3), 7.36 (dd, J = 10.9, 1.6 Hz, 1H, H-4), 7.30 (d, J = 11.4 Hz, 1H, H-6), 7.23 (dd, J = 9.3, 2.9 Hz, 1H, H-10), 7.19 (d, J = 2.9 Hz, 1H, H-8), 6.75 (ddd, J = 7.4, 6.3, 1.6 Hz, 1H, H-2), 3.92–3.90 (m, 4H, H-15, H-17), 3.24–3.22 (m, 4H, H-14, H-18).

^{13}C-NMR (125 MHz, $CDCl_3$) δ (ppm) 147.4 (C-9), 140.6 (C-7), 139.8 (C-3), 131.9 (C-11a), 131.6 (C-4*), 131.5 (C-6*), 129.0 (C-7a), 128.2 (C-1), 117.6 (C-10), 115.6 (C-8), 115.5 (C-11), 113.5 (C-2), 66.9 (2C, C-15, C-17), 49.8 (2C, C-14, C-18). *Carbon not observed in ^{13}C-NMR, assigned by gHSQC.

^{11}B-NMR (160 MHz, $CDCl_3$) δ (ppm) 28.51.

HRMS (EI) *m/z* calculated for $C_{16}H_{17}BN_2O$ $[M]^+$: 264.1428; found $[M]^+$: 264.1428.

Sonogashira Reaction

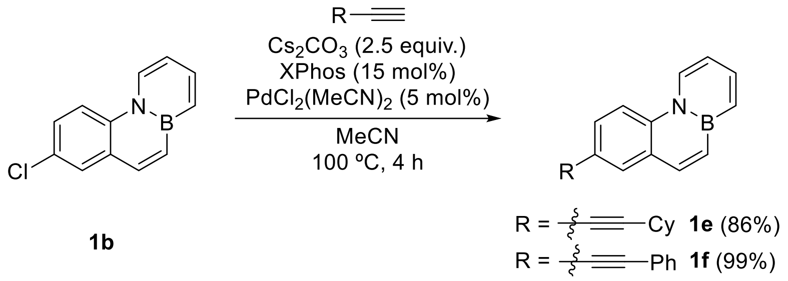

General procedure for the Sonogashira reaction: $PdCl_2(MeCN)_2$ (5 mol %), XPhos (15 mol %), Cs_2CO_3 (2.5 equiv) and BN-phenanthrene **1b** (1.0 equiv) were added to an oven-dried vial equipped with a stir bar. The vial was sealed with a cap lined with a disposable Teflon septum, evacuated under vacuum, and purged with argon three times. Acetonitrile (0.1 M) was then added and the resulting suspension stirred at room temperature for 30 min. The corresponding alkyne (1.3 equiv) was then injected, and the reaction mixture was heated to 100 °C until full consumption of starting material was observed by TLC. The crude mixture was cooled to room temperature and diluted with CH_2Cl_2 and water. The layers were separated, and the aqueous layer was extracted twice with CH_2Cl_2. The combined organic layers were dried over anhydrous Na_2SO_4, filtered and concentrated under reduced pressure. The remaining residue was purified by flash column chromatography to give the corresponding alkyne products.

Preparation of 7-(cyclohex-1-en-1-ylethynyl)-4a-aza-10a-boraphenanthrene (1e).

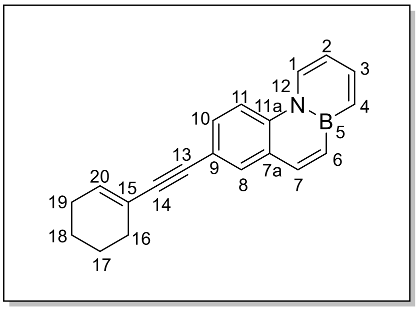

Following the general procedure, $PdCl_2(MeCN)_2$ (2.5 mg, 0.0095 mmol), XPhos (14 mg, 0.029 mmol), Cs_2CO_3 (156 mg, 0.479 mmol) and BN-phenanthrene **1b** (40 mg, 0.19 mmol) were dissolved in acetonitrile (2 mL). Then, 1-ethynylcyclohexene (29 μL, 0.24 mmol) was injected, and the mixture was heated to 100 °C for 4 h. Purification by

flash column chromatography (1% EtOAc/hexane) gave **1e** (46 mg, 0.16 mmol, 86%) as a brown solid.

M. p.: 75–77 °C.

IR (KBr) ῦmax (cm^{-1}) 3027, 2929, 2857, 1618, 1555, 1515, 1470, 1396, 1302, 1265, 796, 731.

^{1}H-NMR (500 MHz, CDCl$_3$) δ (ppm) 8.59 (d, *J* = 7.4 Hz, 1H, H-1), 8.12 (d, *J* = 8.9 Hz, 1H, H-11), 7.90 (d, *J* = 11.4 Hz, 1H, H-7), 7.86 (d, *J* = 2.0 Hz, 1H, H-8), 7.75 (dd, *J* = 11.0, 6.2 Hz, 1H, H-3), 7.62 (dd, *J* = 8.9, 2.0 Hz, 1H, H-10), 7.37 (dd, *J* = 11.0, 1.5 Hz, 1H, H-4), 7.32 (d, *J* = 11.4 Hz, 1H, H-6), 6.77 (ddd, *J* = 7.4, 6.2, 1.5 Hz, 1H, H-2), 6.27–6.24 (m, 1H, H-20), 2.29–2.25 (m, 2H, H-16), 2.18–2.14 (m, 2H, H-19), 1.72–1.67 (m, 2H, H-17), 1.65–1.60 (m, 2H, H-18).

^{13}C-NMR (125 MHz, CDCl$_3$) δ (ppm) 140.4 (C-3), 140.3 (C-7), 137.1 (C-11a), 135.4 (C-20), 133.8 (C-8), 131.7 (C-4*), 131.3 (C-6*), 130.8 (C-10), 128.1 (C-7a), 128.0 (C-1), 120.7 (C-15), 119.1 (C-9), 114.7 (C-11), 114.0 (C-2), 91.5 (C-14), 86.2 (C-13), 29.3 (C-16), 25.8 (C-19), 22.4 (C-17), 21.5 (C-18). *Carbon not observed in ^{13}C-NMR, assigned by gHSQC.

^{11}B-NMR (160 MHz, CDCl$_3$) δ (ppm) 29.01.

HRMS (EI) *m/z* calculated for $C_{20}H_{18}BN$ $[M]^+$: 283.1527; found $[M]^+$: 283.1526.

Preparation of 7-(phenylethynyl)-4a-aza-10a-boraphenanthrene (1f).

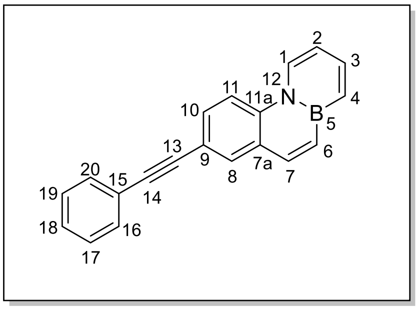

Following the general procedure, PdCl$_2$(MeCN)$_2$ (1.8 mg, 0.0070 mmol), XPhos (10 mg, 0.021 mmol), Cs_2CO_3 (115 mg, 0.353 mmol) and BN-phenanthrene **1b** (30 mg, 0.14 mmol) were dissolved in acetonitrile (1.5 mL). Then, phenylacetylene (20 μL, 0.18 mmol) was injected, and the mixture was heated to 100 °C for 4 h. Purification by flash column chromatography (5% CH_2Cl_2/hexane) gave **1f** (39 mg, 0.14 mmol, 99%) as a white solid.

M. p.: 97–99 °C.

IR (KBr) ῦmax (cm^{-1}) 3027, 1617, 1554, 1452, 1441, 898, 806, 758, 733, 692.

^{1}H-NMR (500 MHz, CDCl$_3$) δ (ppm) 8.62 (d, *J* = 7.5 Hz, 1H, H-1), 8.18 (d, *J* = 8.9 Hz, 1H, H-11), 7.98 (d, *J* = 2.1 Hz, 1H, H-8), 7.94 (d, *J* = 11.4 Hz, 1H, H-7), 7.77 (dd, *J* = 11.0, 6.3 Hz, 1H, H-3), 7.74 (dd, *J* = 8.9, 2.1 Hz, 1H, H-10), 7.58–7.55 (m, 2H, H-16, H-20), 7.40–7.34 (m, 5H, H-4, H-6, H-17, H-18, H-19), 6.79 (ddd, *J* = 7.5, 6.3, 1.7 Hz, 1H, H-2).

^{13}C-NMR (125 MHz, $CDCl_3$) δ (ppm) 140.5 (C-3), 140.3 (C-7), 137.5 (C-11a), 134.1 (C-8), 132.1 (C-4*), 131.61 (2C, C-16, C-20), 131.60 (C-6*), 130.9 (C-10), 128.4 (2C, C-17, C-19), 128.3 (C-18), 128.1 (C-7a), 128.0 (C-1), 123.2 (C-15), 118.5 (C-9), 114.9 (C-11), 114.2 (C-2), 89.7 (C-14), 88.9 (C-13). *Carbon not observed in ^{13}C-NMR, assigned by gHSQC.

^{11}B-NMR (160 MHz, $CDCl_3$) δ (ppm) 29.17.

HRMS (EI) *m/z* calculated for $C_{20}H_{14}BN$ $[M]^+$: 279.1214; found $[M]^+$: 279.1219.

UV–VIS AND FLUORESCENCE DATA FOR 1f IN DIFFERENT SOLVENTS

Solvent	UV/Vis λ_{max} (nm)	Emission[a] λ_{max} (nm)	Φ_F[b] (%)
**Cyclohexane (CH)**	278, 292, 311, **323**, 336, 345	**348**, 364	0.65
**Dichloromethane (DCM)**	280, 293, 313, **324**, 337	352, **366**	0.29
**Tetrahydrofuran (THF)**	278, 293, 312, **324**, 336	350, **365**	0.19
**1,4-Dioxane**	279, 292, 313, **324**, 336	350, **365**	0.39
**Acetonitrile (MeCN)**	280, 291, 312, **322**, 334	351, **365**	0.26

[a]Emission at 323 nm. [b]Quantum yield relative to 9,10-diphenylanthracene (Φ_F = 0.93) at room temperature. All measures were realized with 7-(phenylethynyl)-4a-aza-10a-boraphenanthrene (**1f**) as substrate at a concentration of 10^{-5} M.

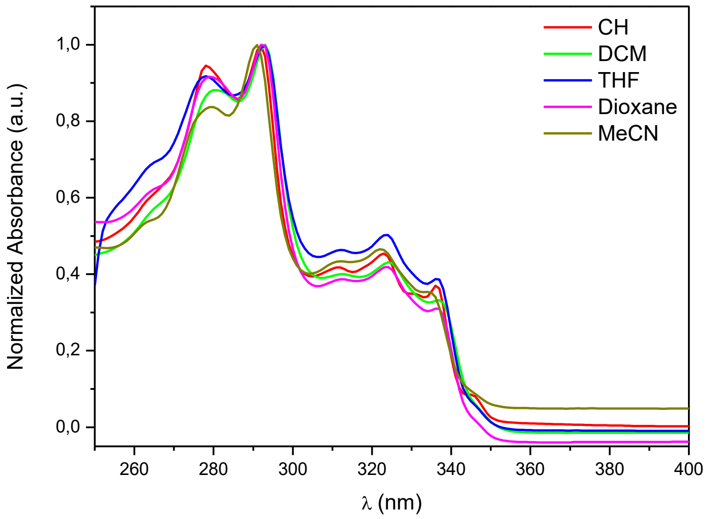

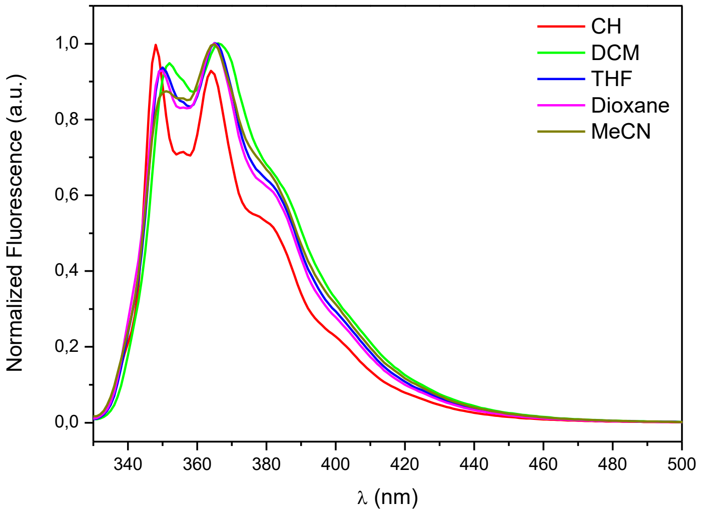

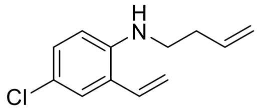

2

^{1}H-NMR (300 MHz, $CDCl_3$)

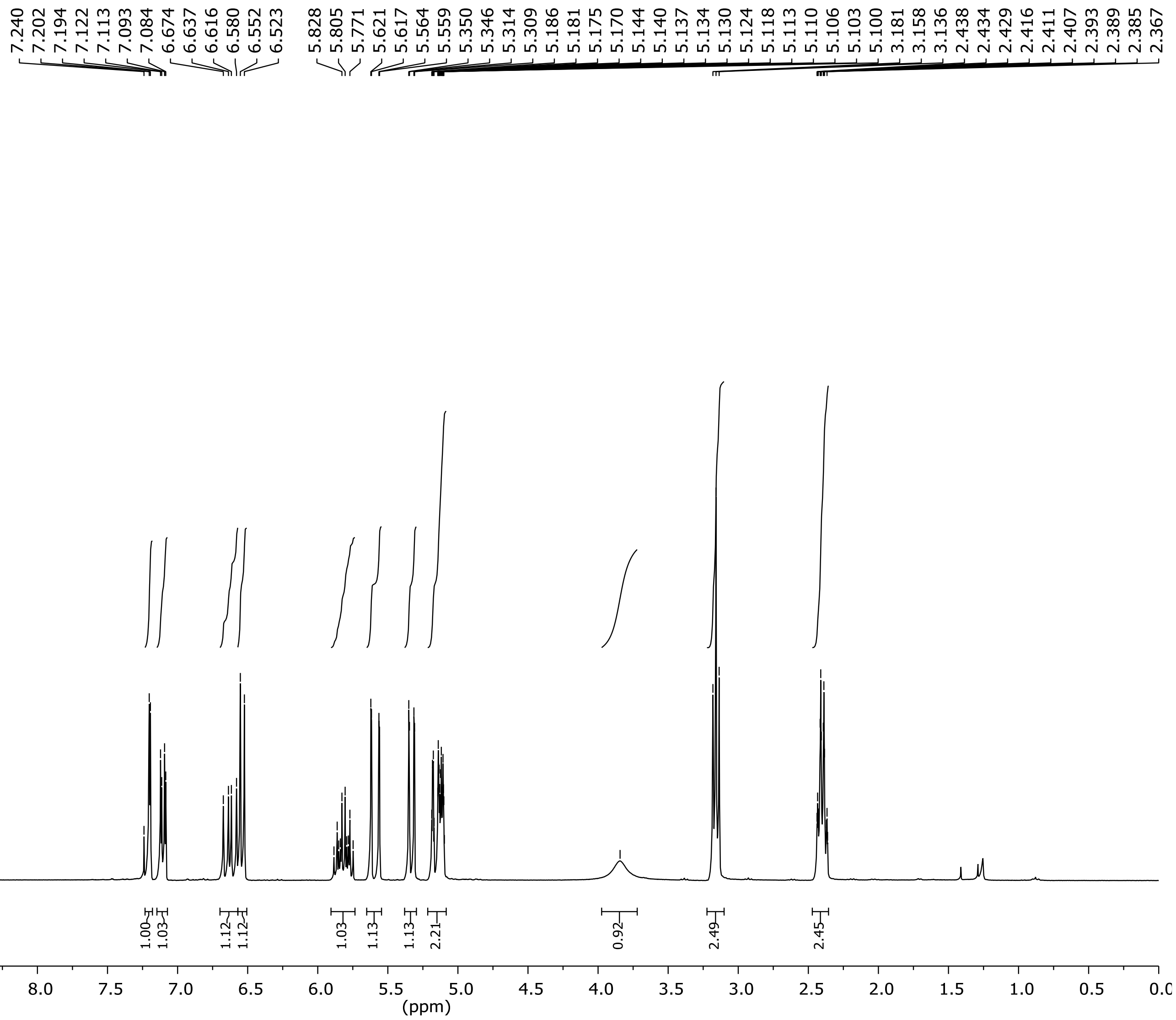

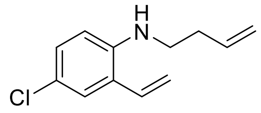

2

^{13}C-NMR (75 MHz, CDCl$_3$)

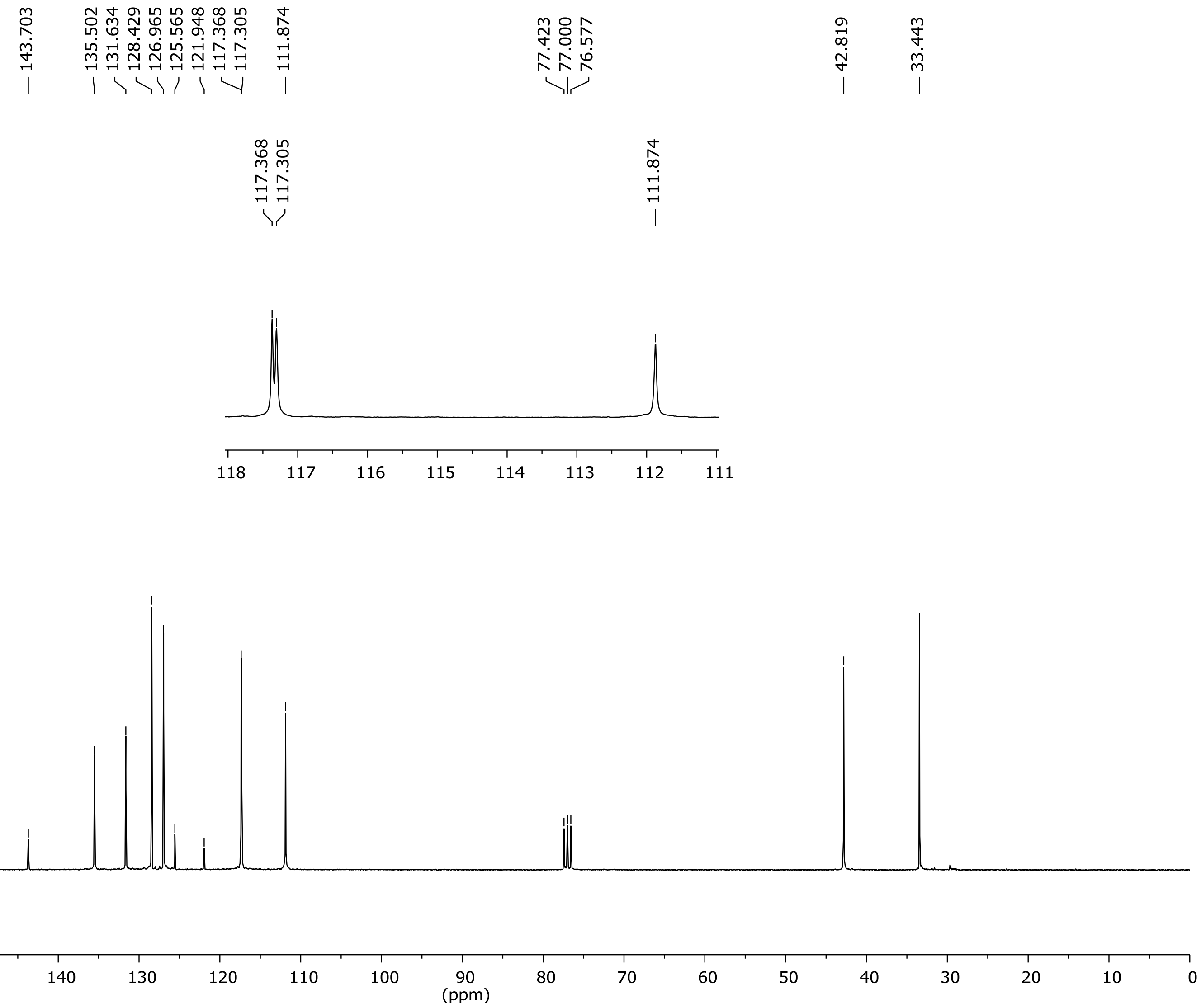

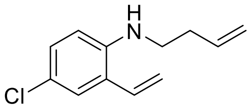

2

gCOSY-NMR ($CDCl_3$)

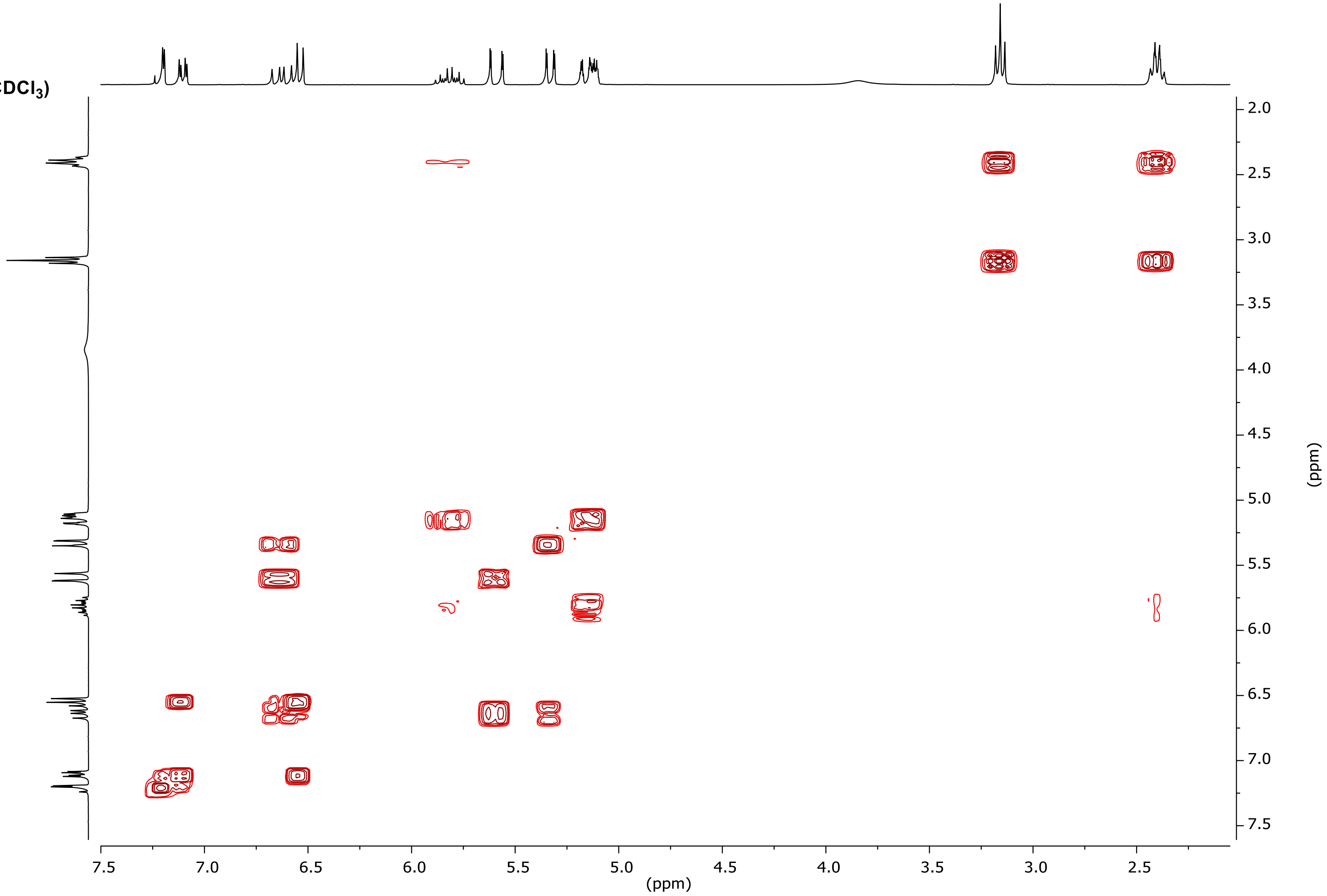

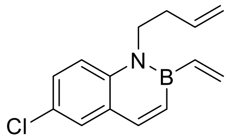

3

^{1}H-NMR (500 MHz, $CDCl_3$)

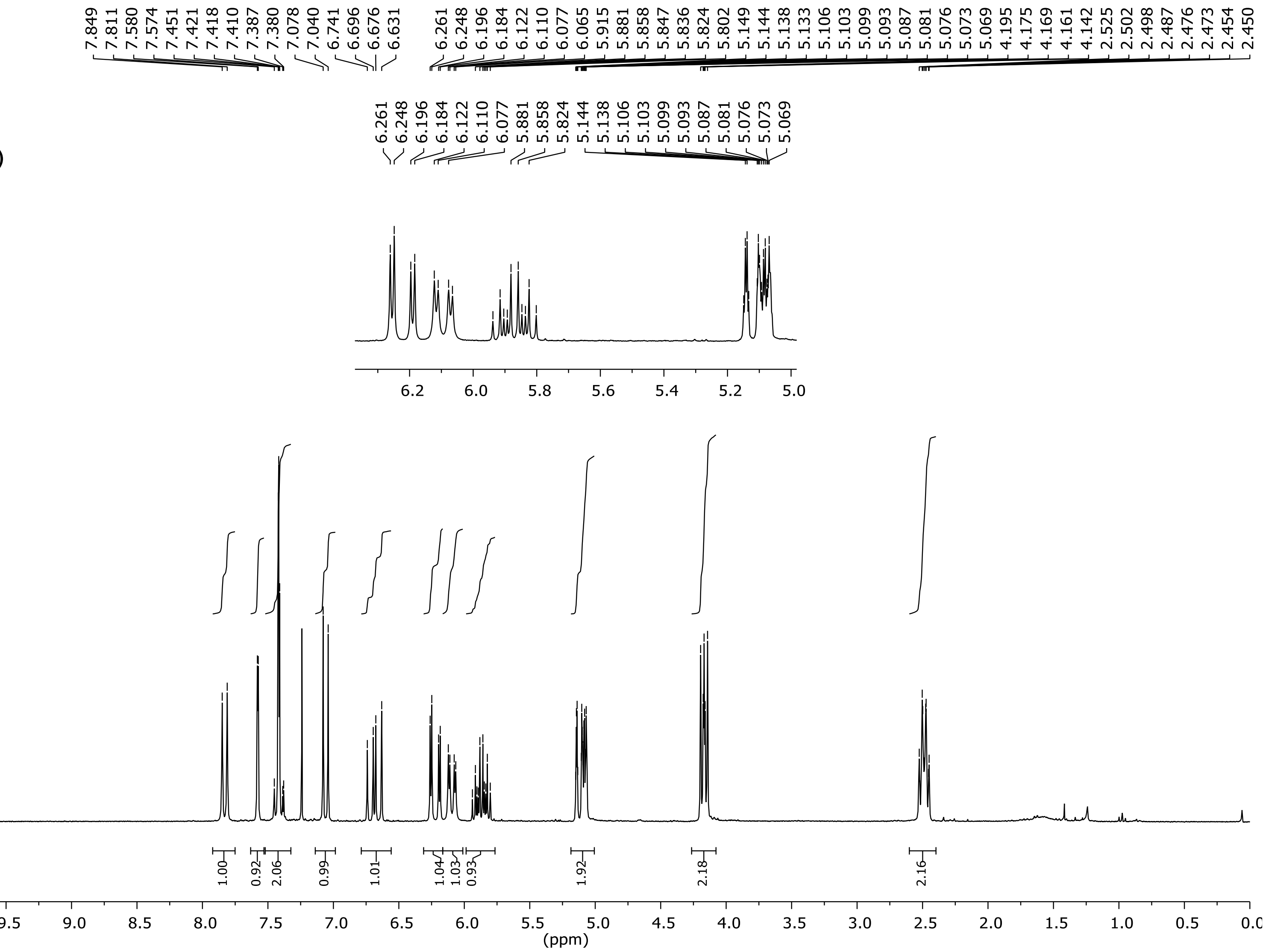

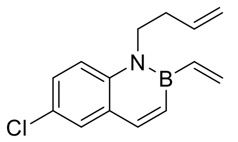

3

^{13}C-NMR (125 MHz, CDCl$_3$)

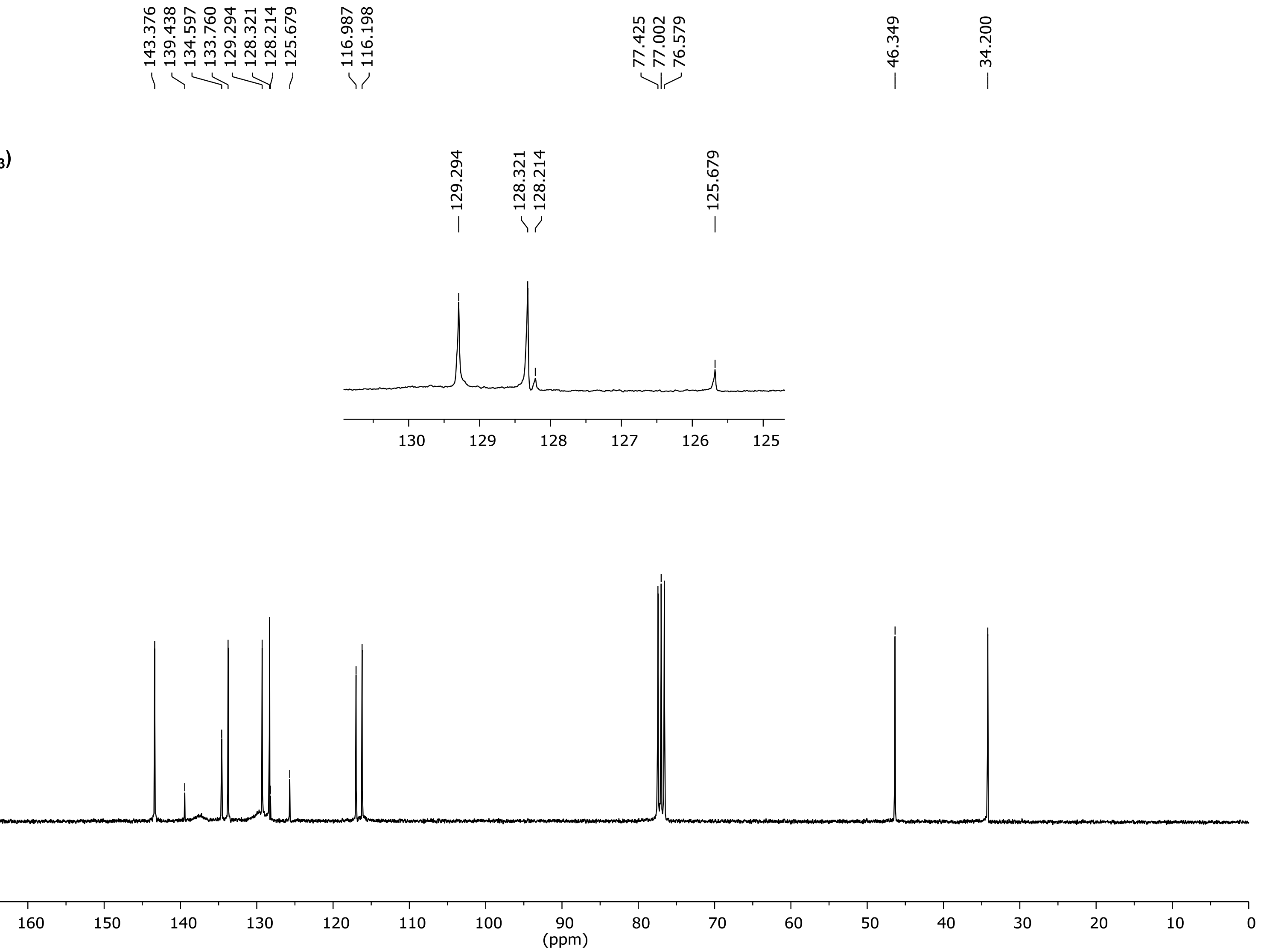

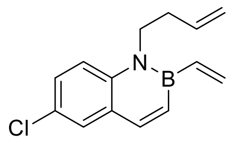

3

^{11}B-NMR (160 MHz, $CDCl_3$)

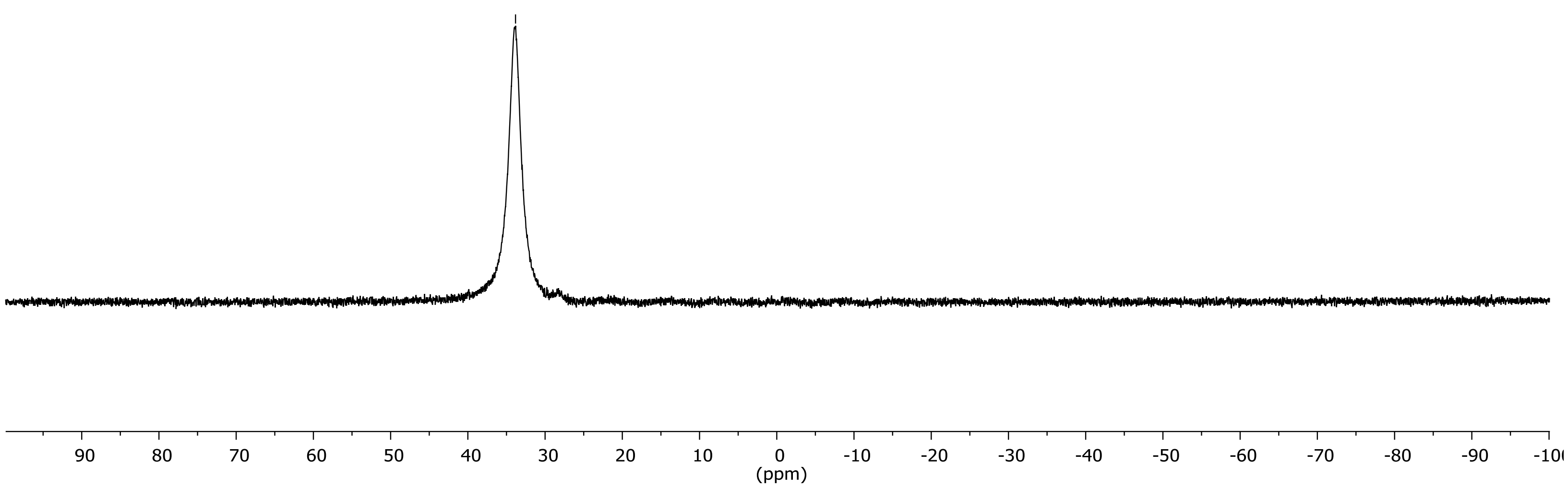

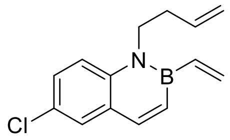

3

gCOSY-NMR ($CDCl_3$)

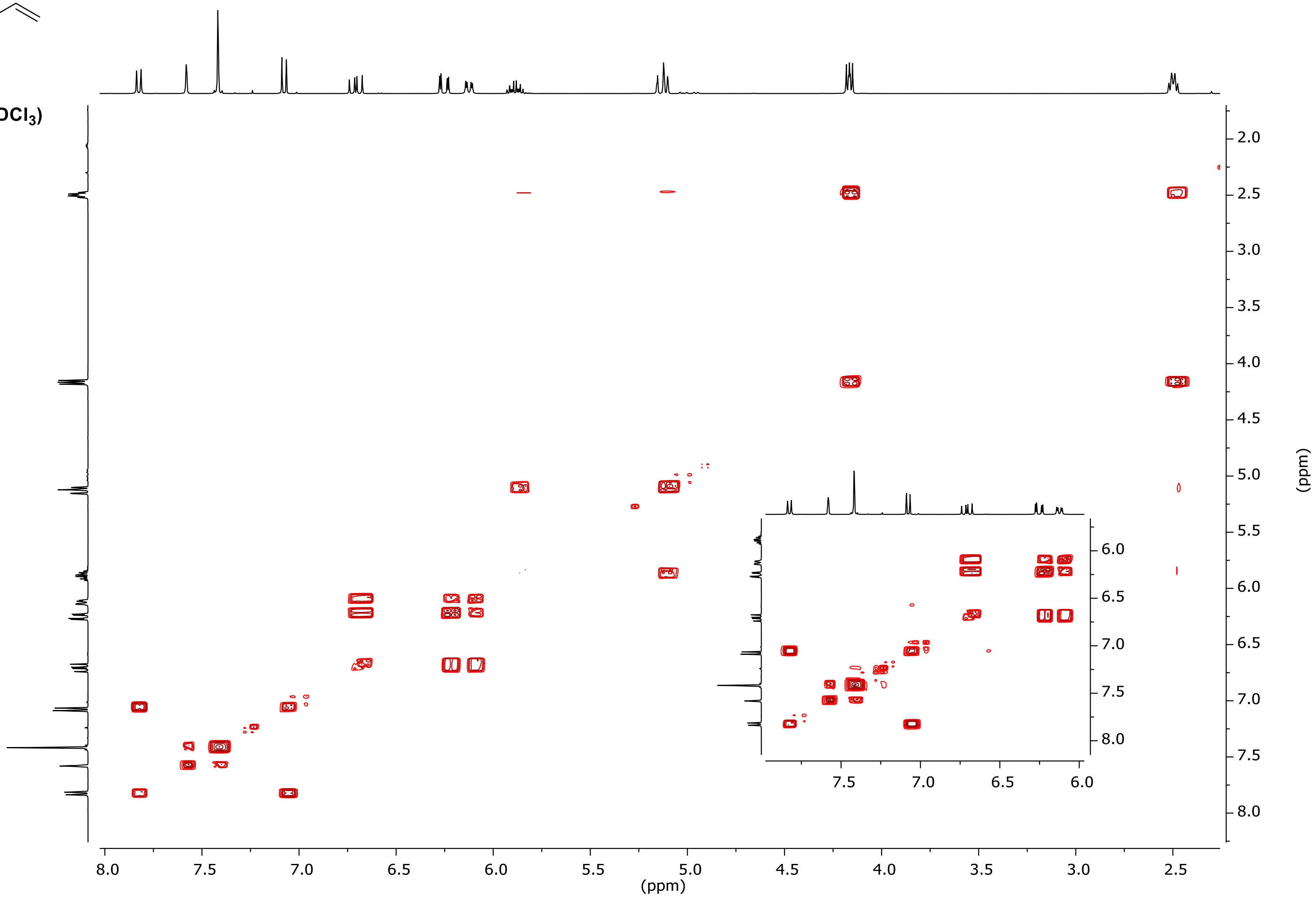

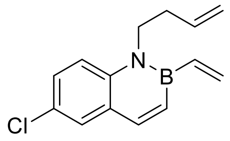

3

gHSQC-NMR ($CDCl_3$)

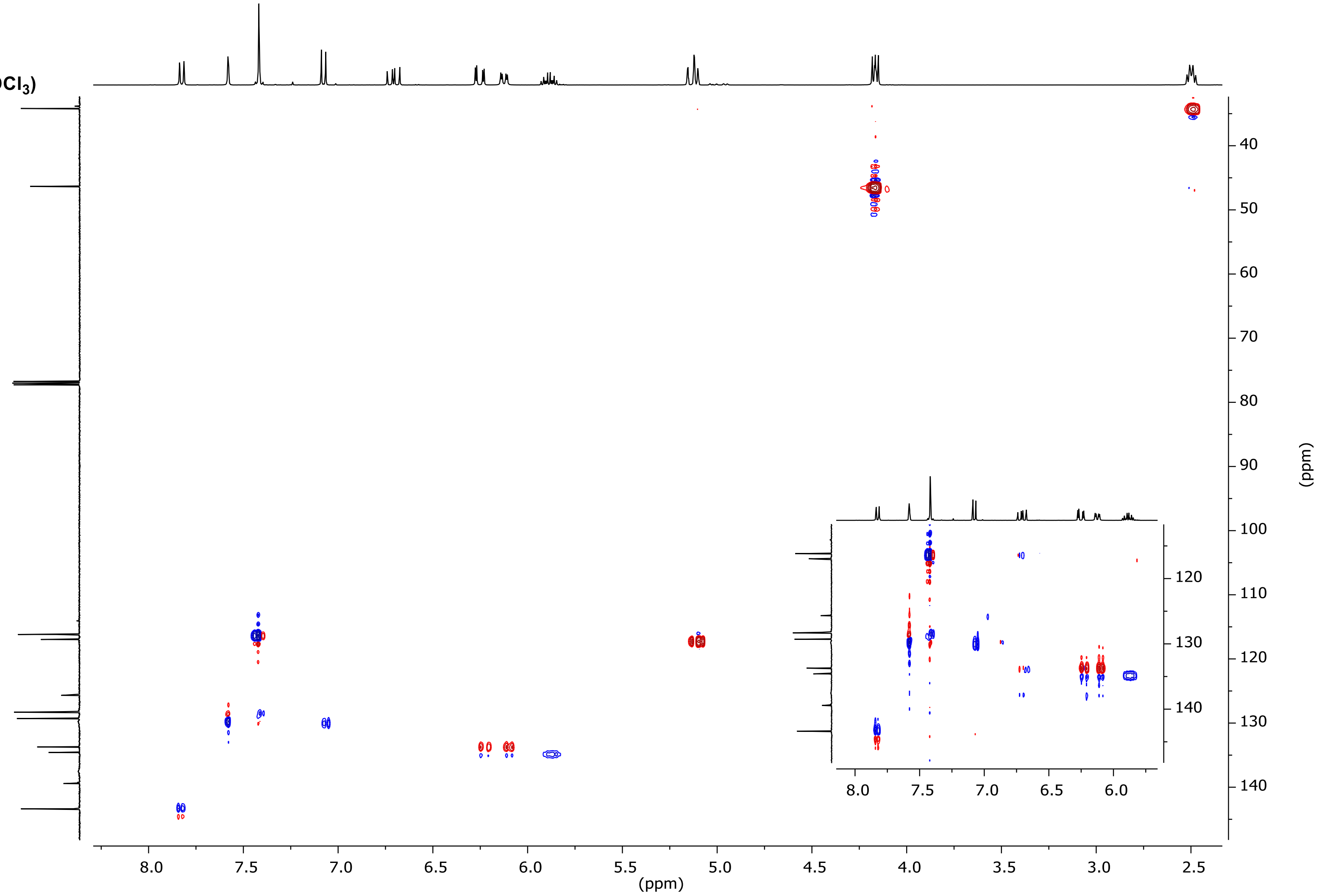

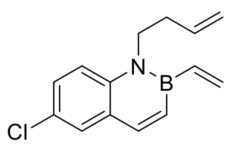

3

gHMBC-NMR ($CDCl_3$)

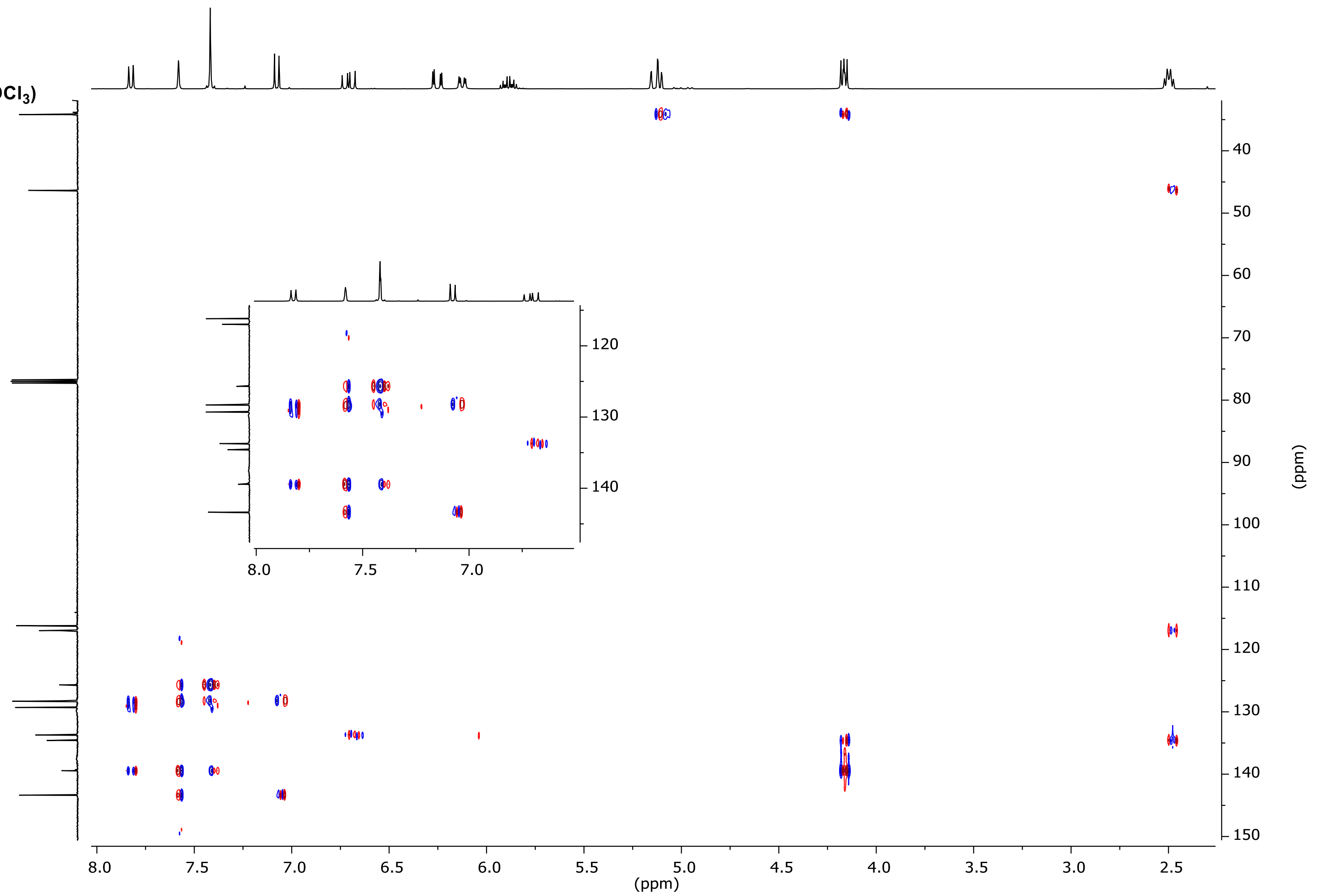

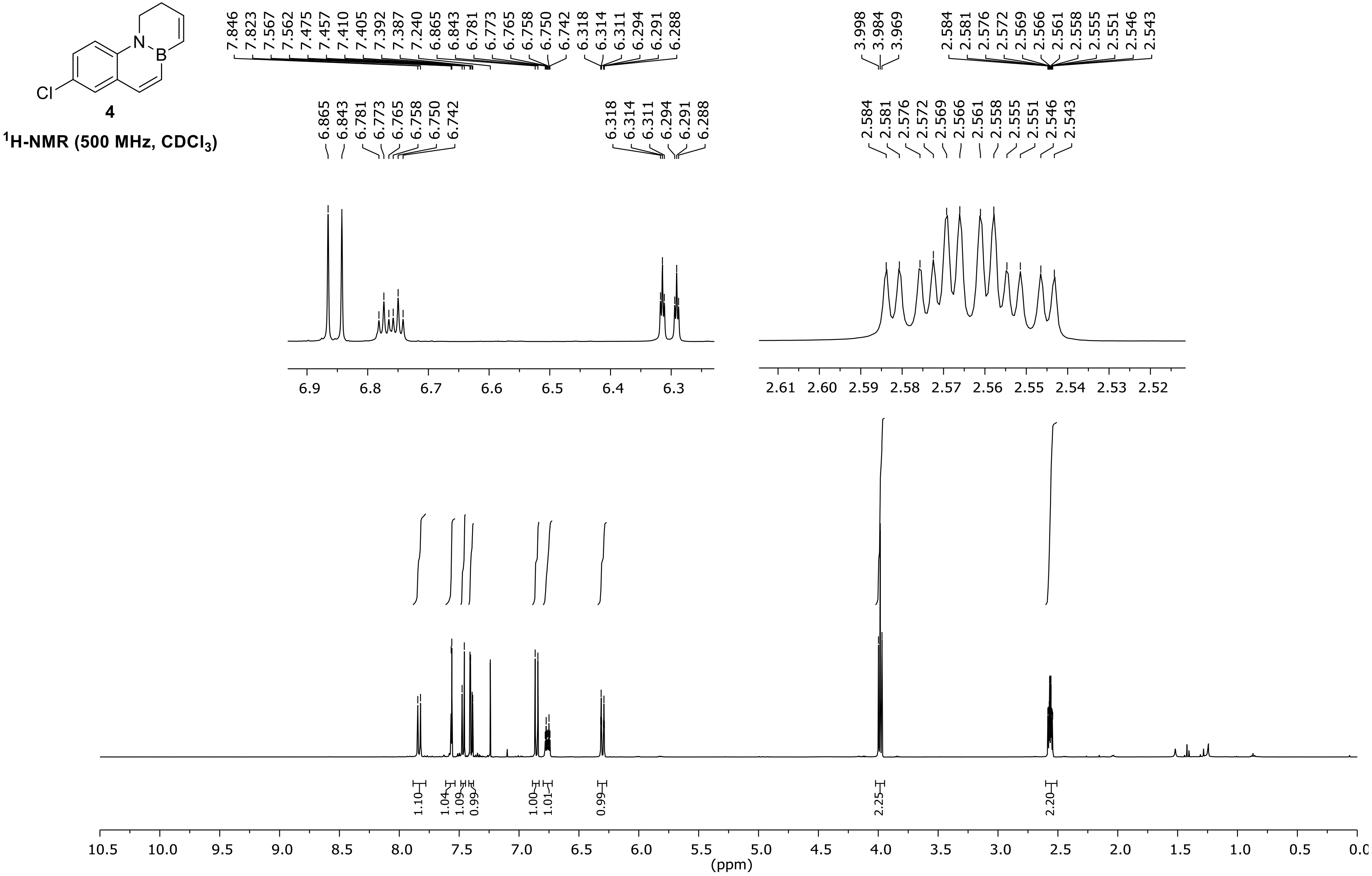
4
^{1}H-NMR (500 MHz, $CDCl_3$)
(ppm)

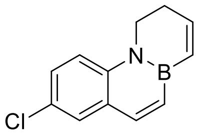

4

^{13}C-NMR (125 MHz, CDCl$_3$)

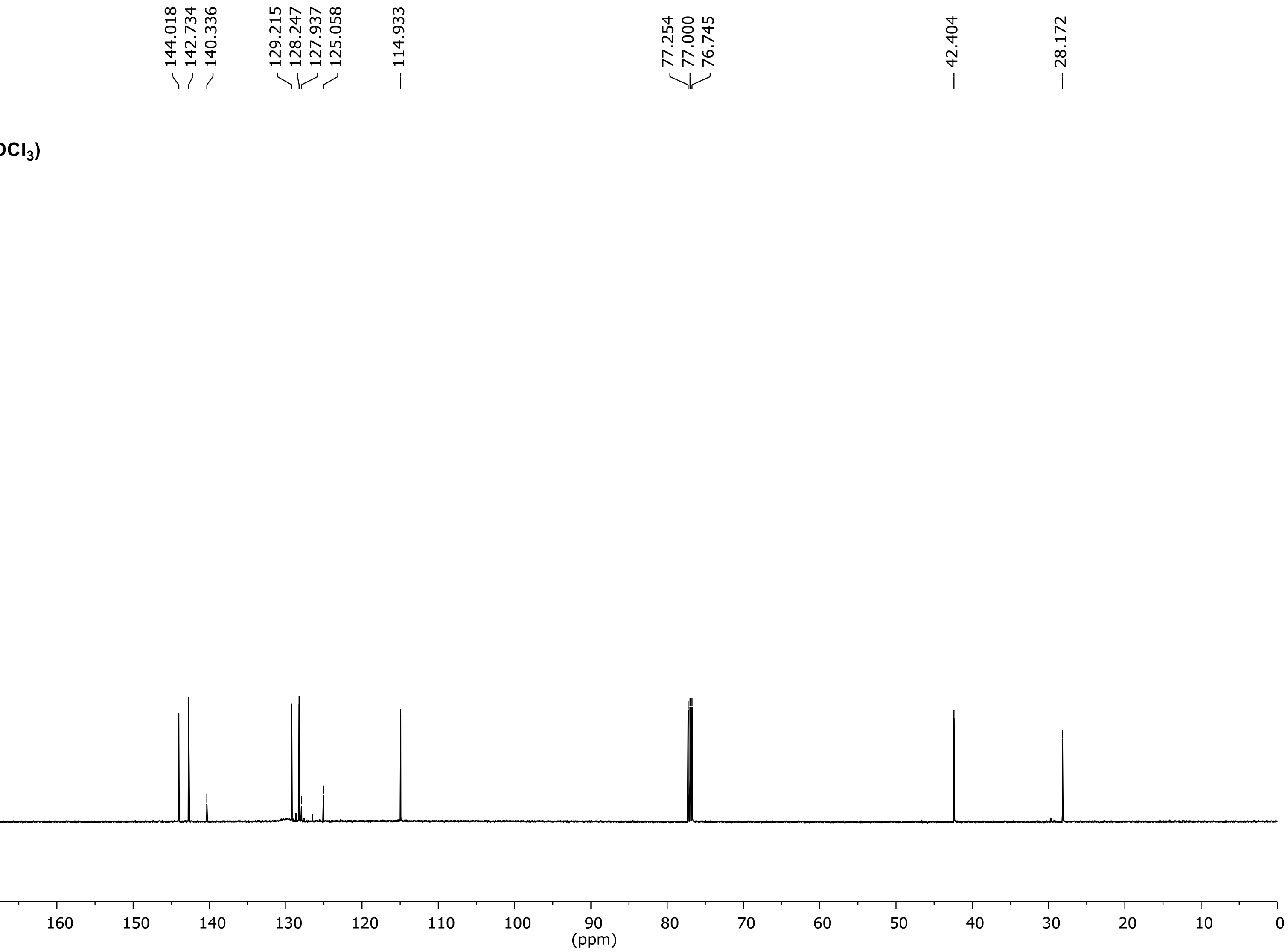

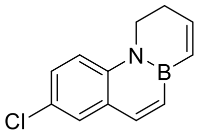

4

^{11}B-NMR (160 MHz, CDCl$_3$)

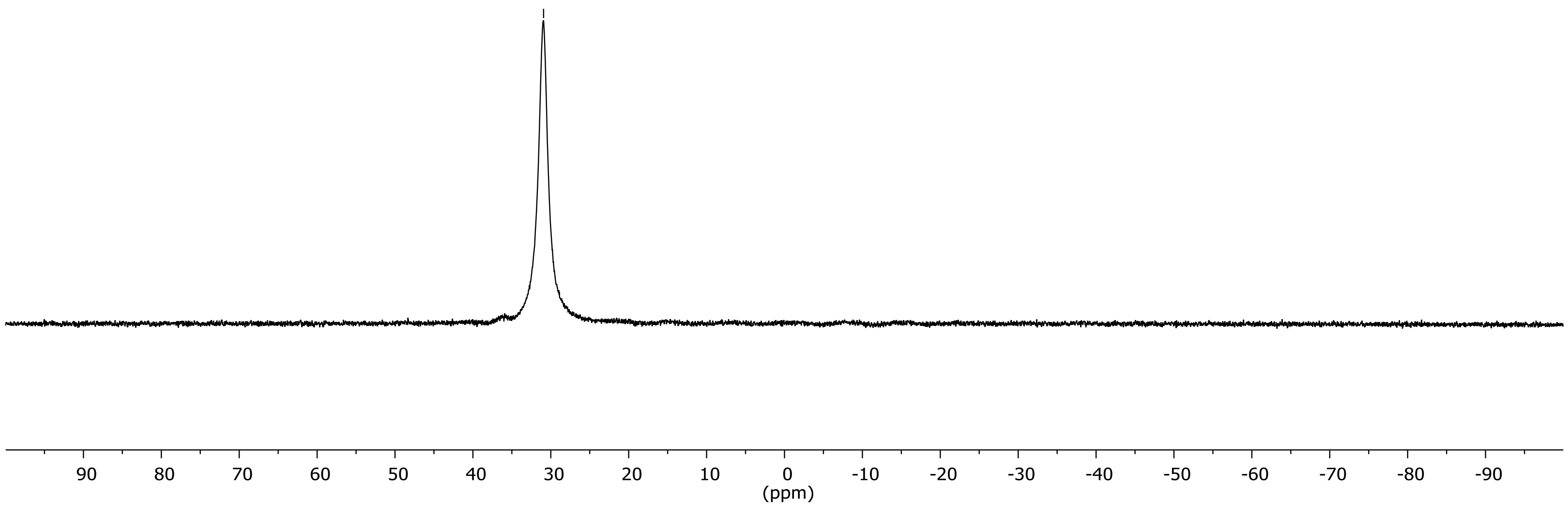

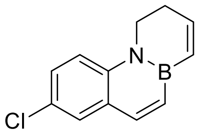

4

gCOSY-NMR ($CDCl_3$)

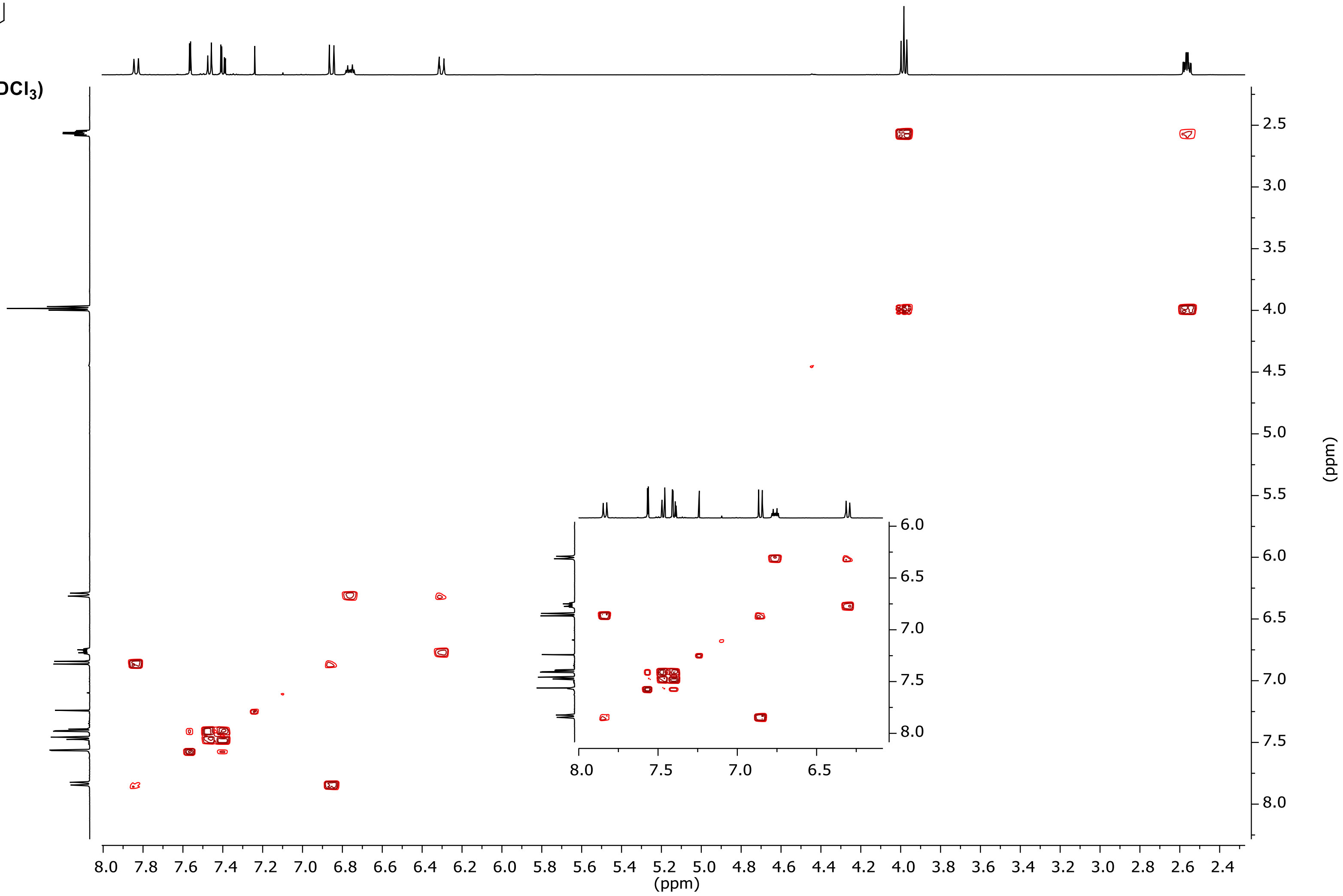

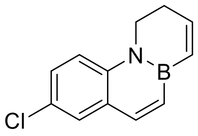

4

gHSQC-NMR ($CDCl_3$)

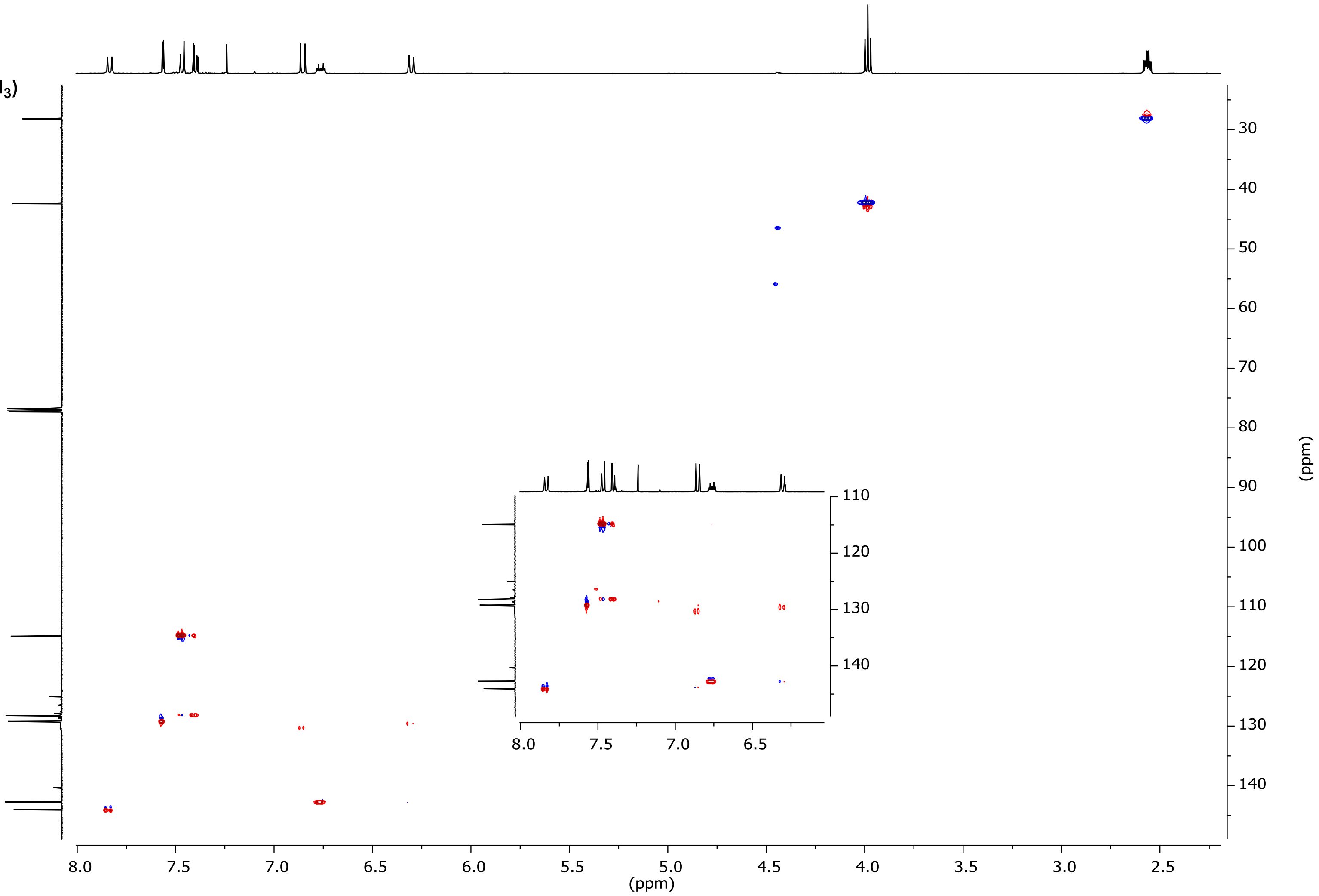

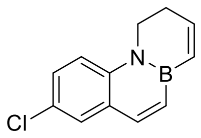

4

gHMBC-NMR ($CDCl_3$)

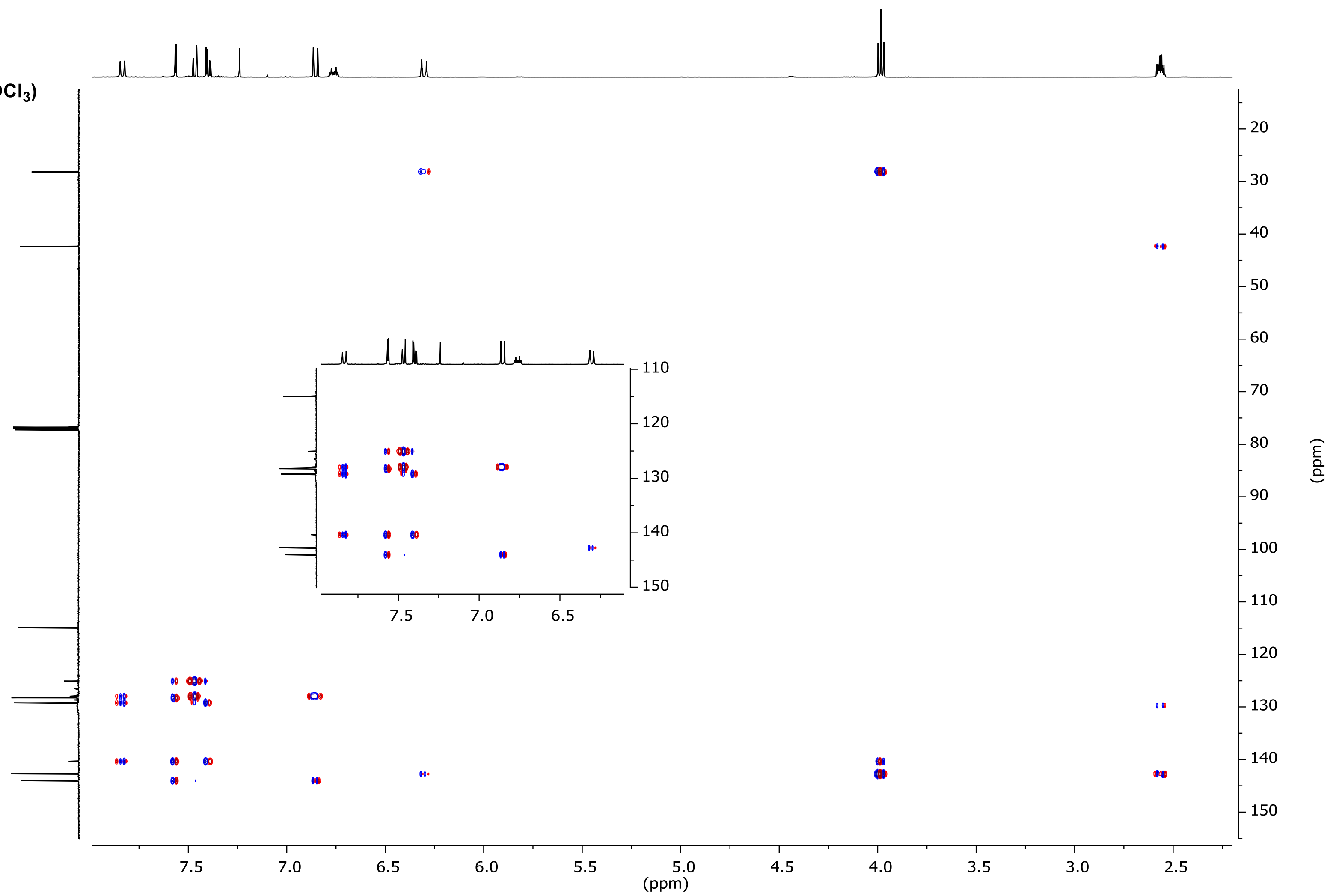

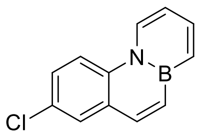

1b

^{1}H-NMR (500 MHz, $CDCl_3$)

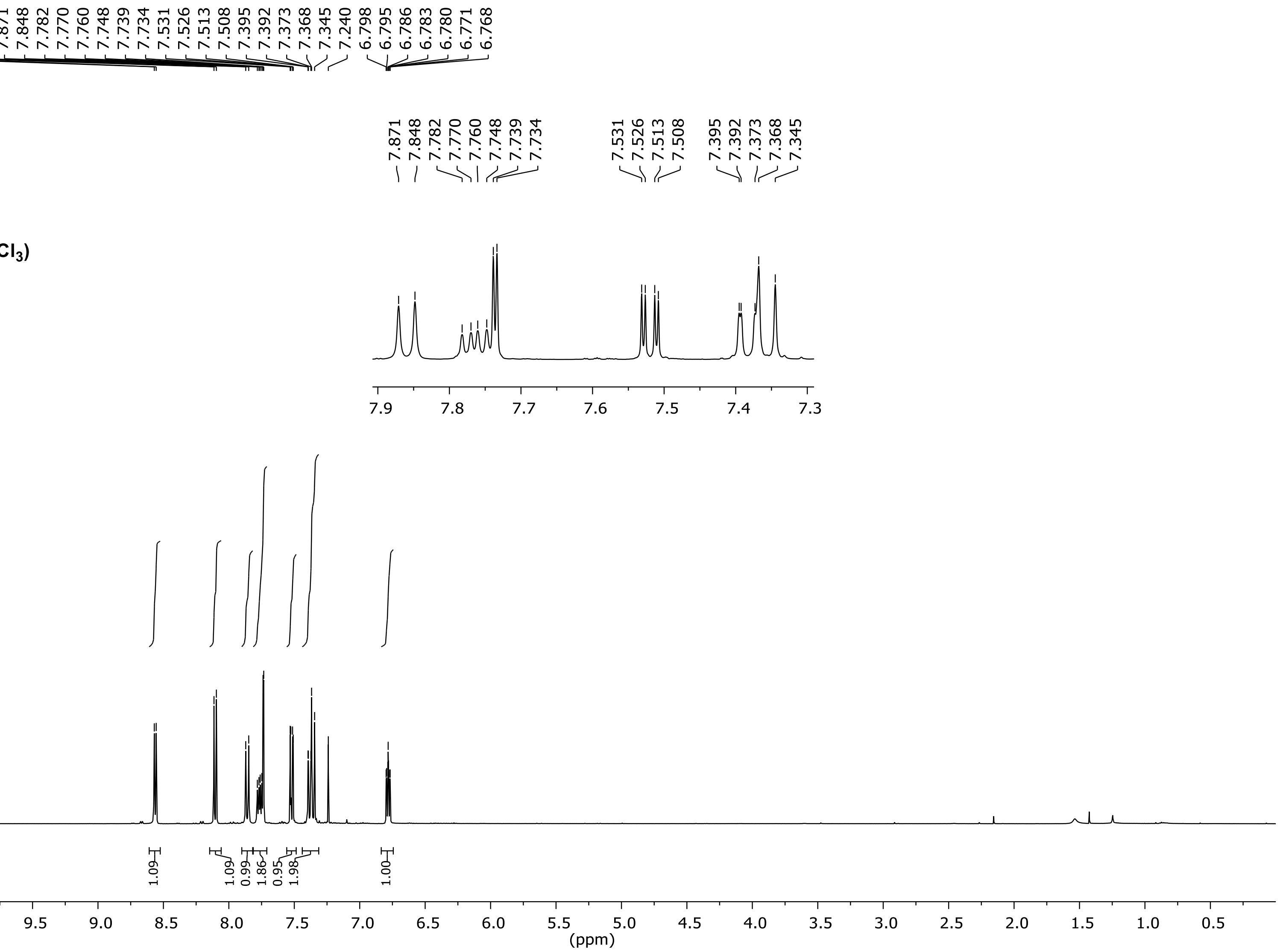

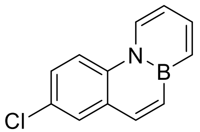

1b

^{13}C-NMR (125 MHz, CDCl$_3$)

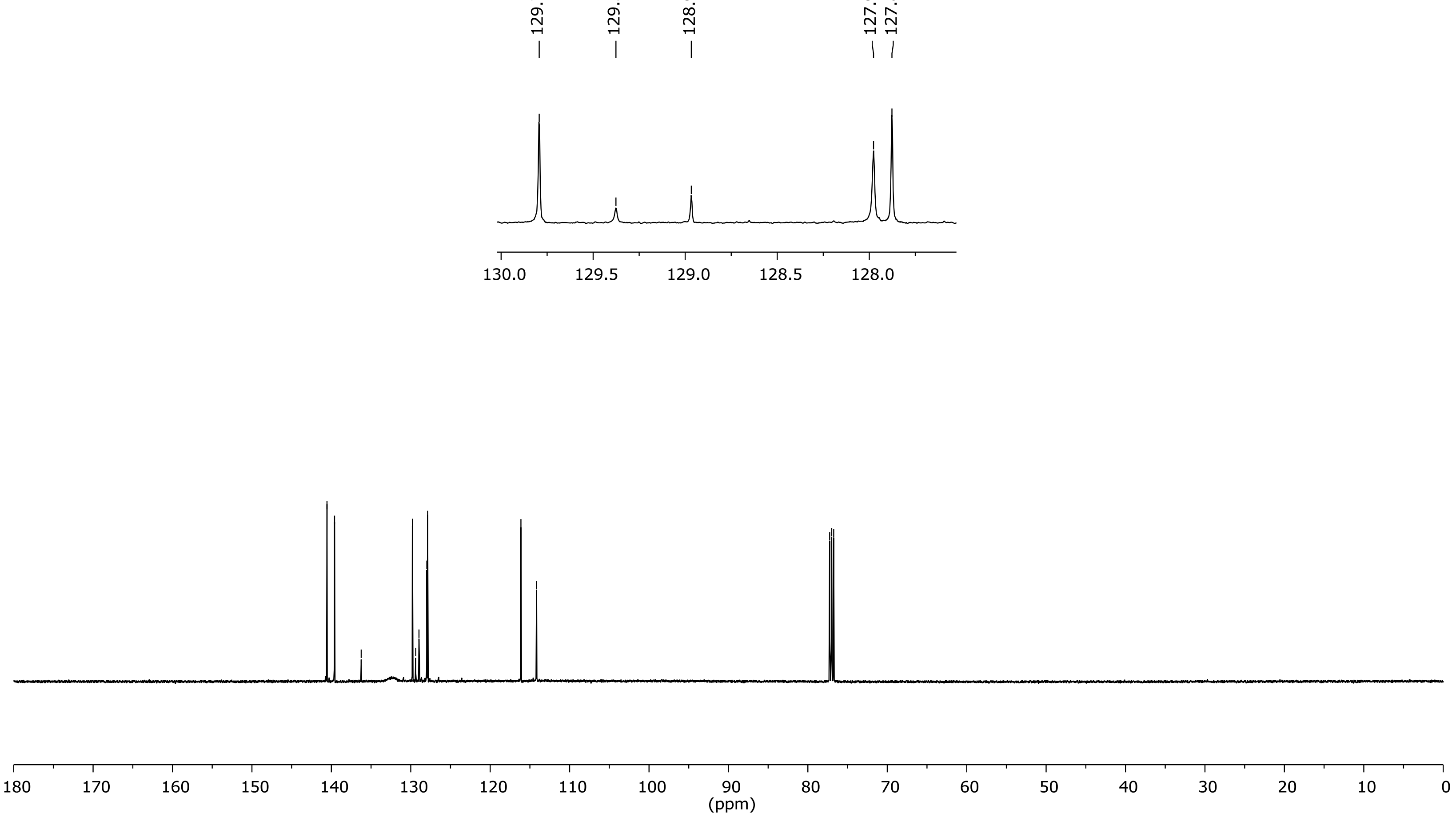

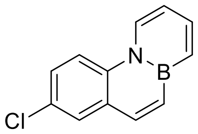

1b

^{11}B-NMR (160 MHz, $CDCl_3$)

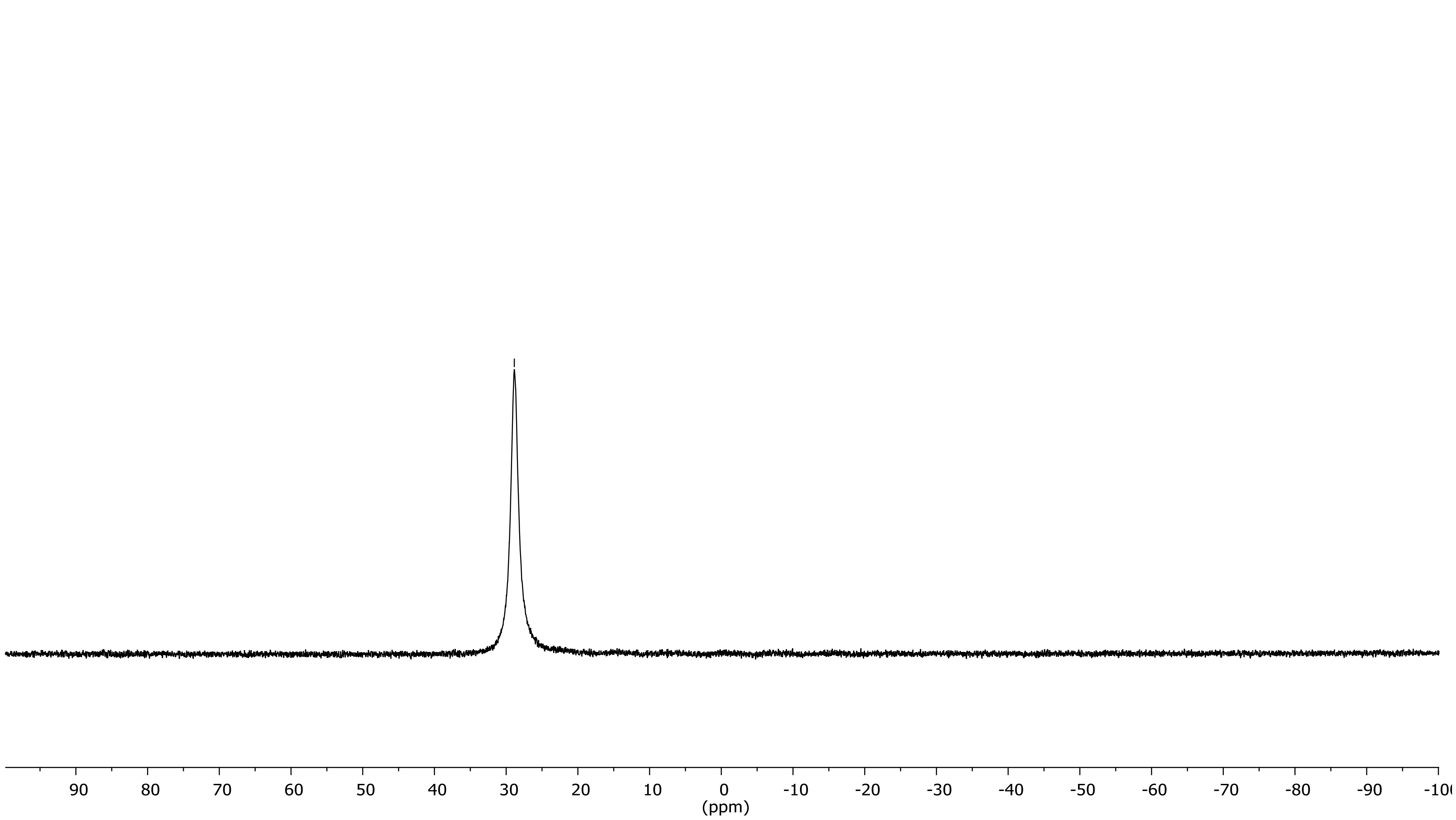

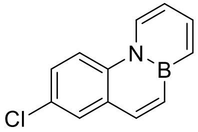

1b

gCOSY-NMR ($CDCl_3$)

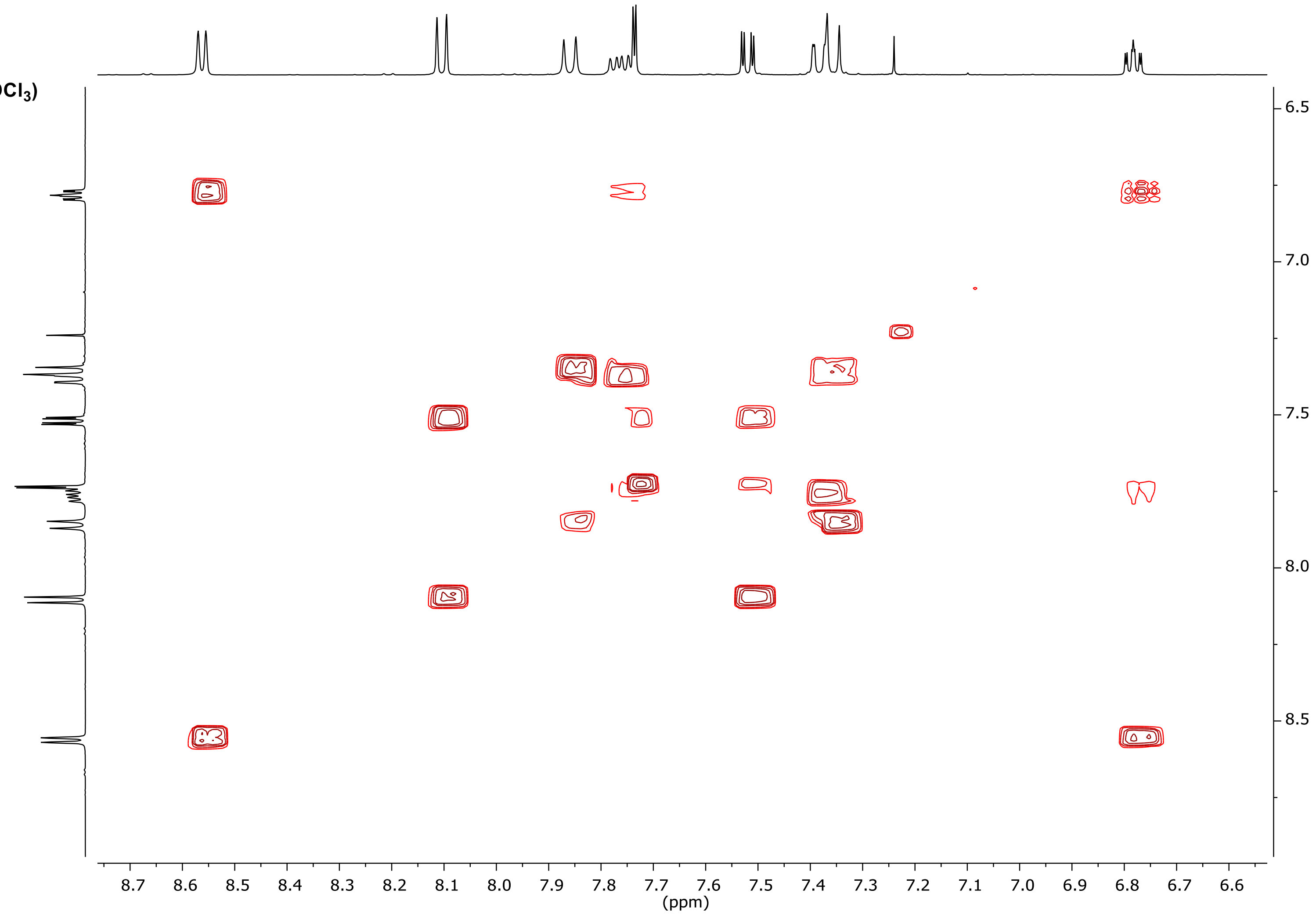

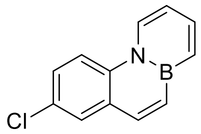

1b

TOCSY-NMR ($CDCl_3$)

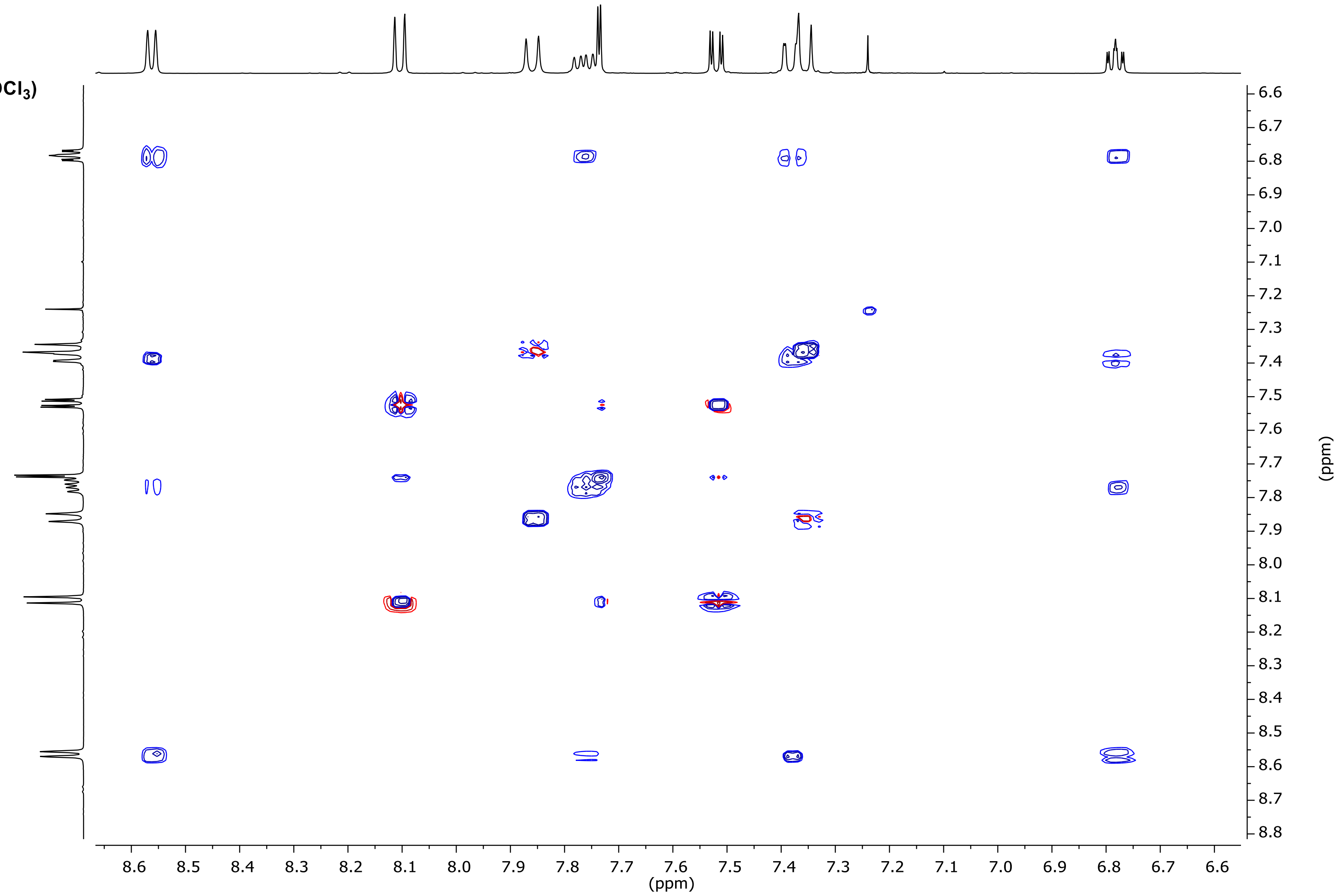

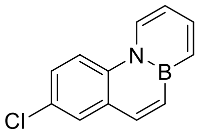

1b

gHSQC-NMR ($CDCl_3$)

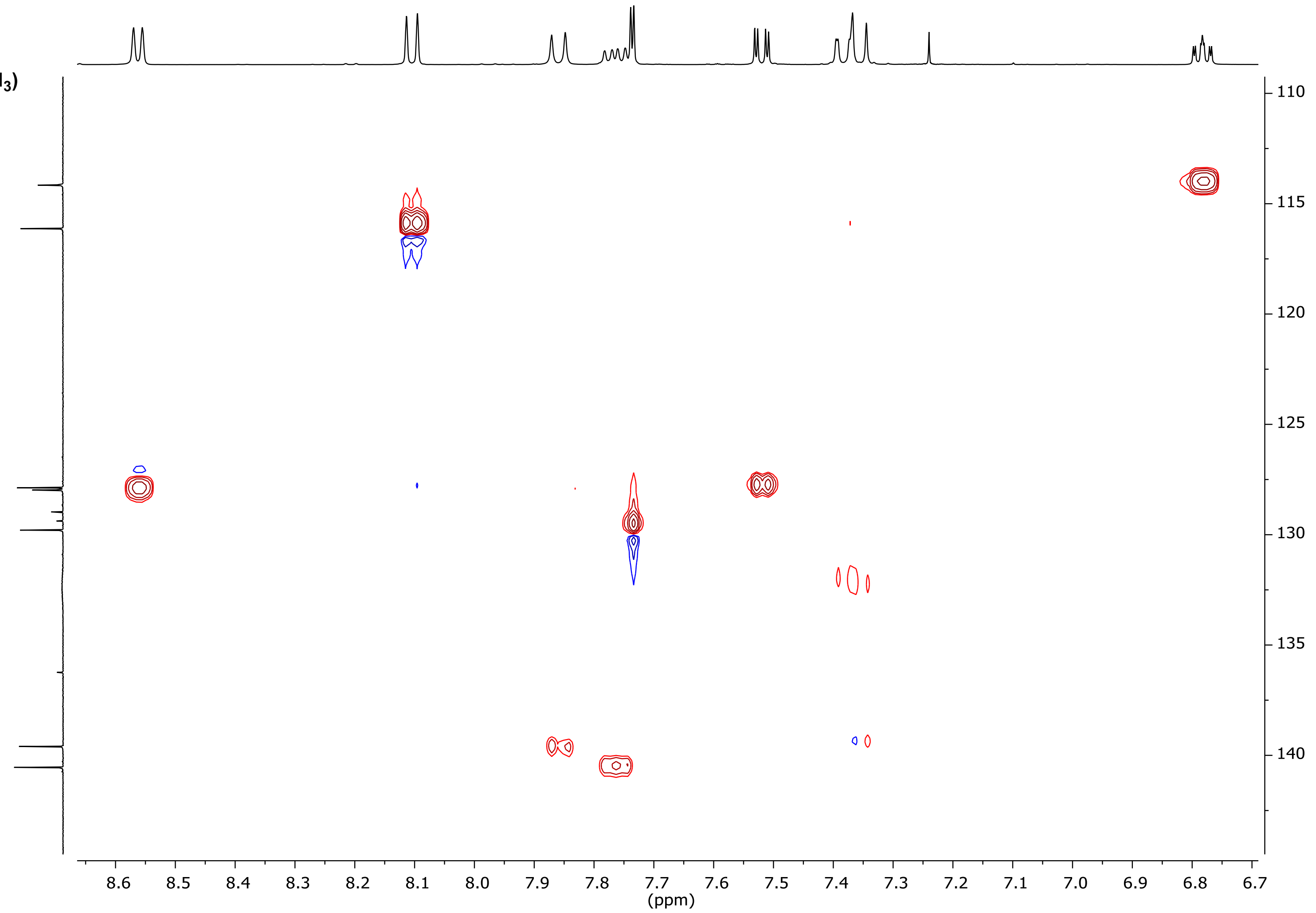

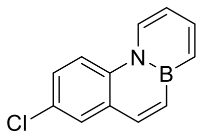

1b

gHMBC-NMR ($CDCl_3$)

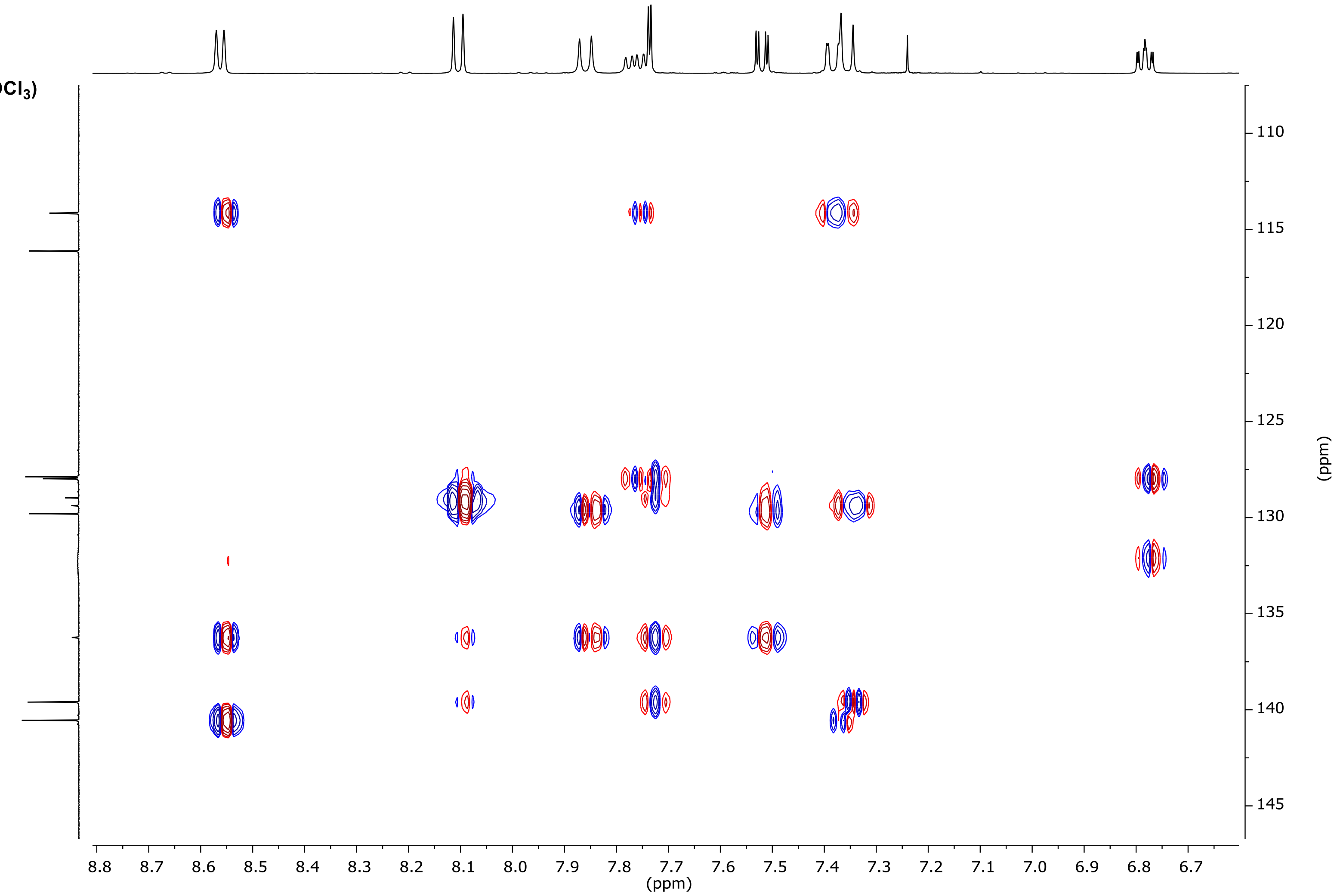

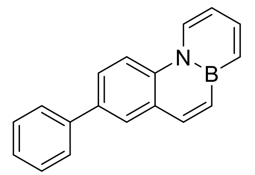

1c

^{1}H-NMR (500 MHz, $CDCl_3$)

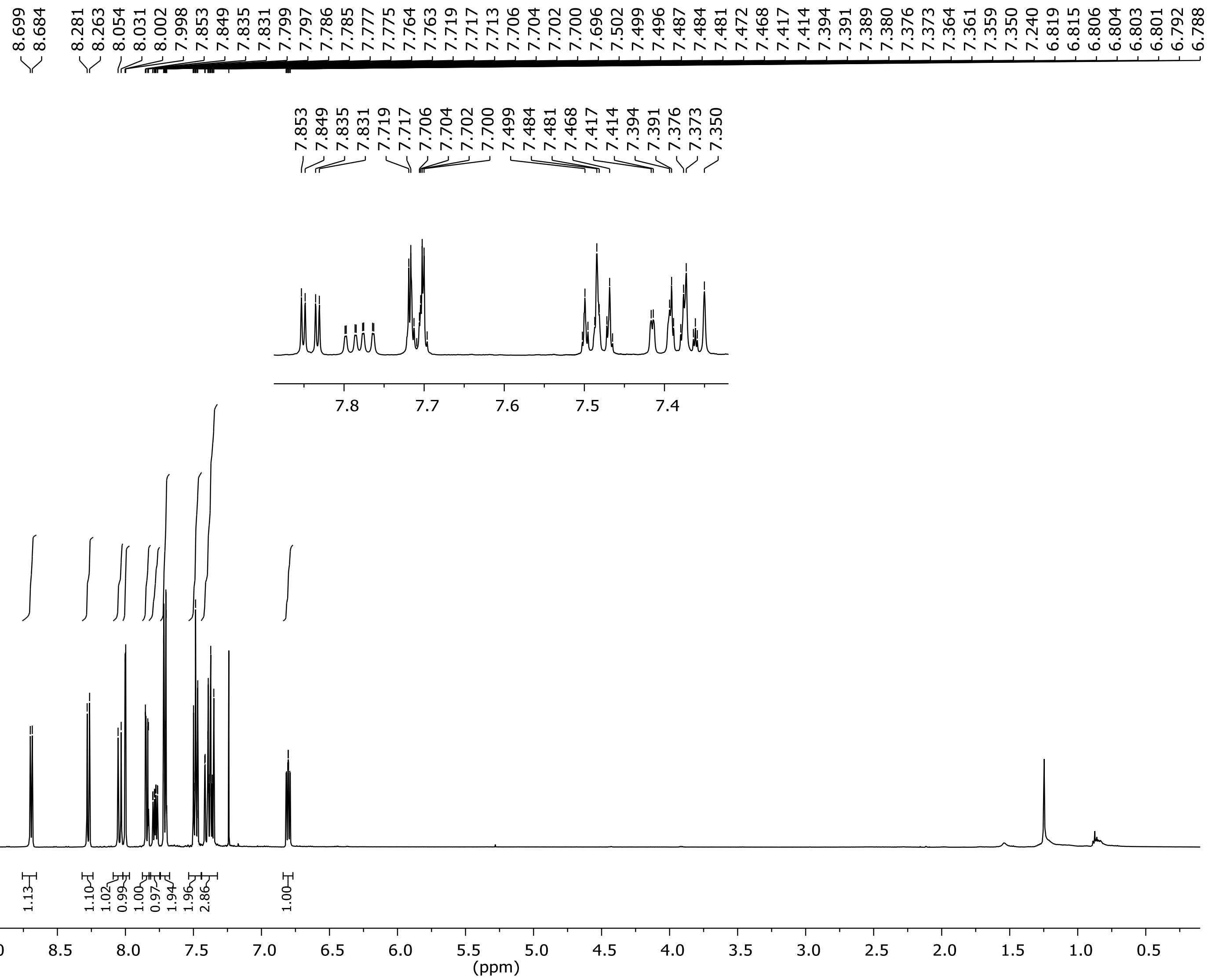

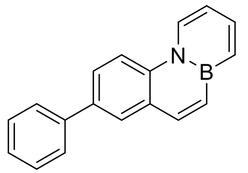

1c

^{13}C-NMR (125 MHz, CDCl$_3$)

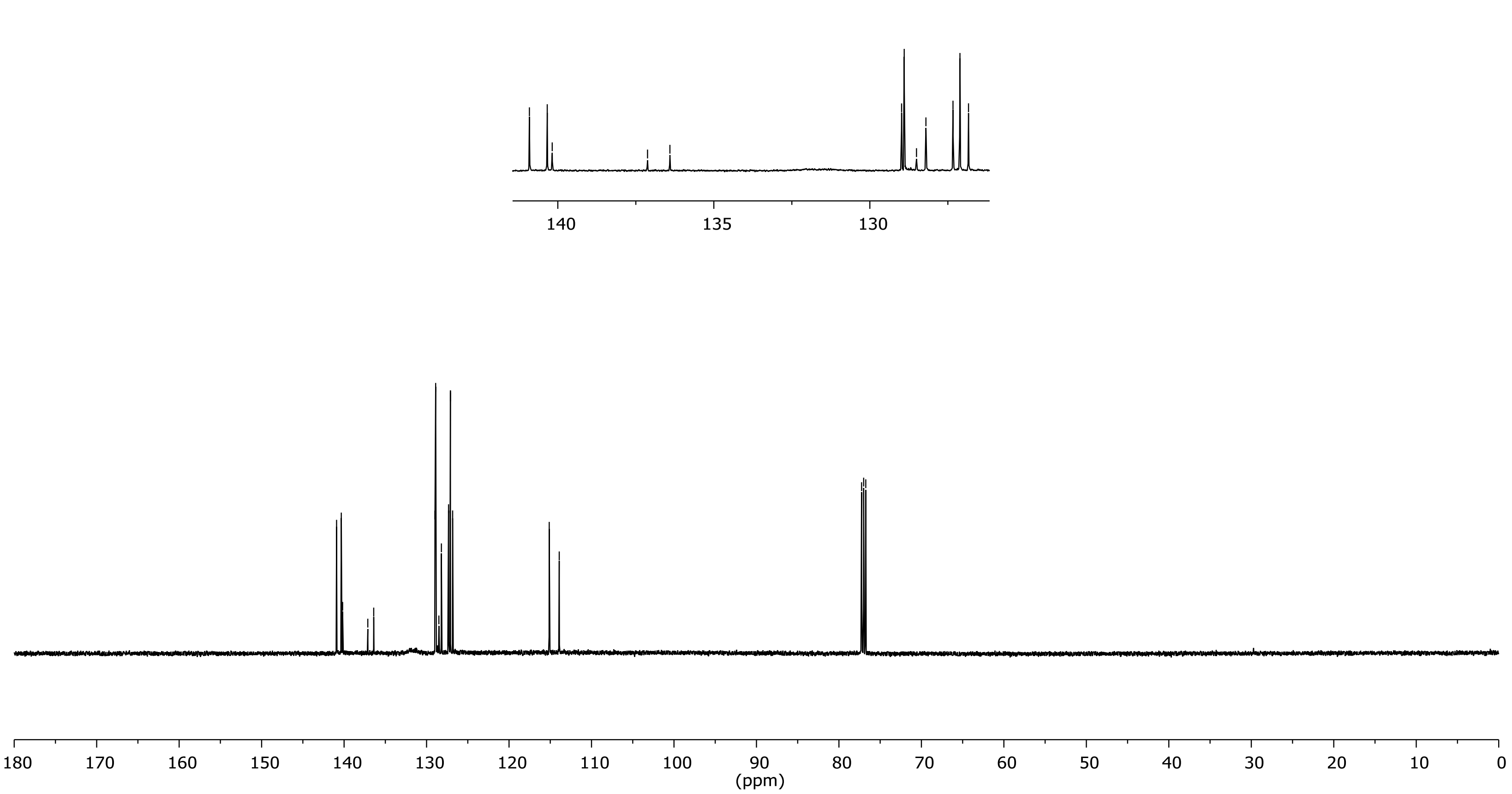

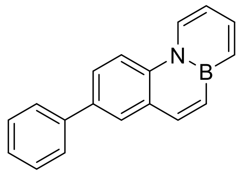

1c

^{11}B-NMR (160 MHz, $CDCl_3$)

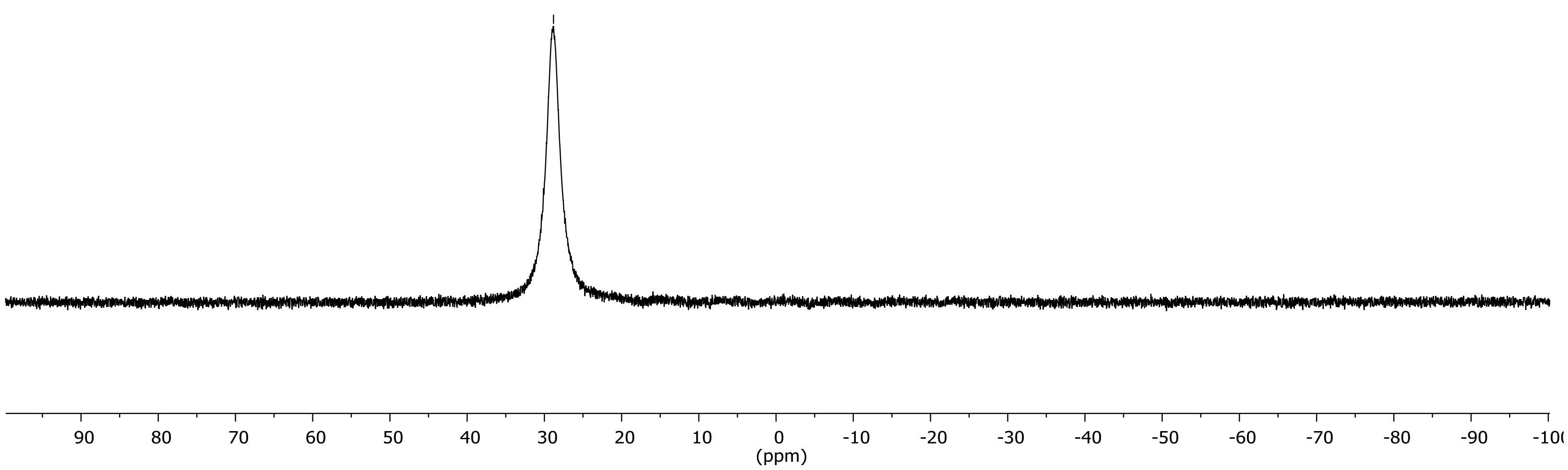

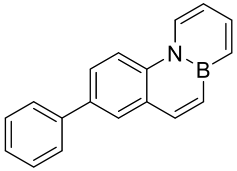

1c

gCOSY-NMR ($CDCl_3$)

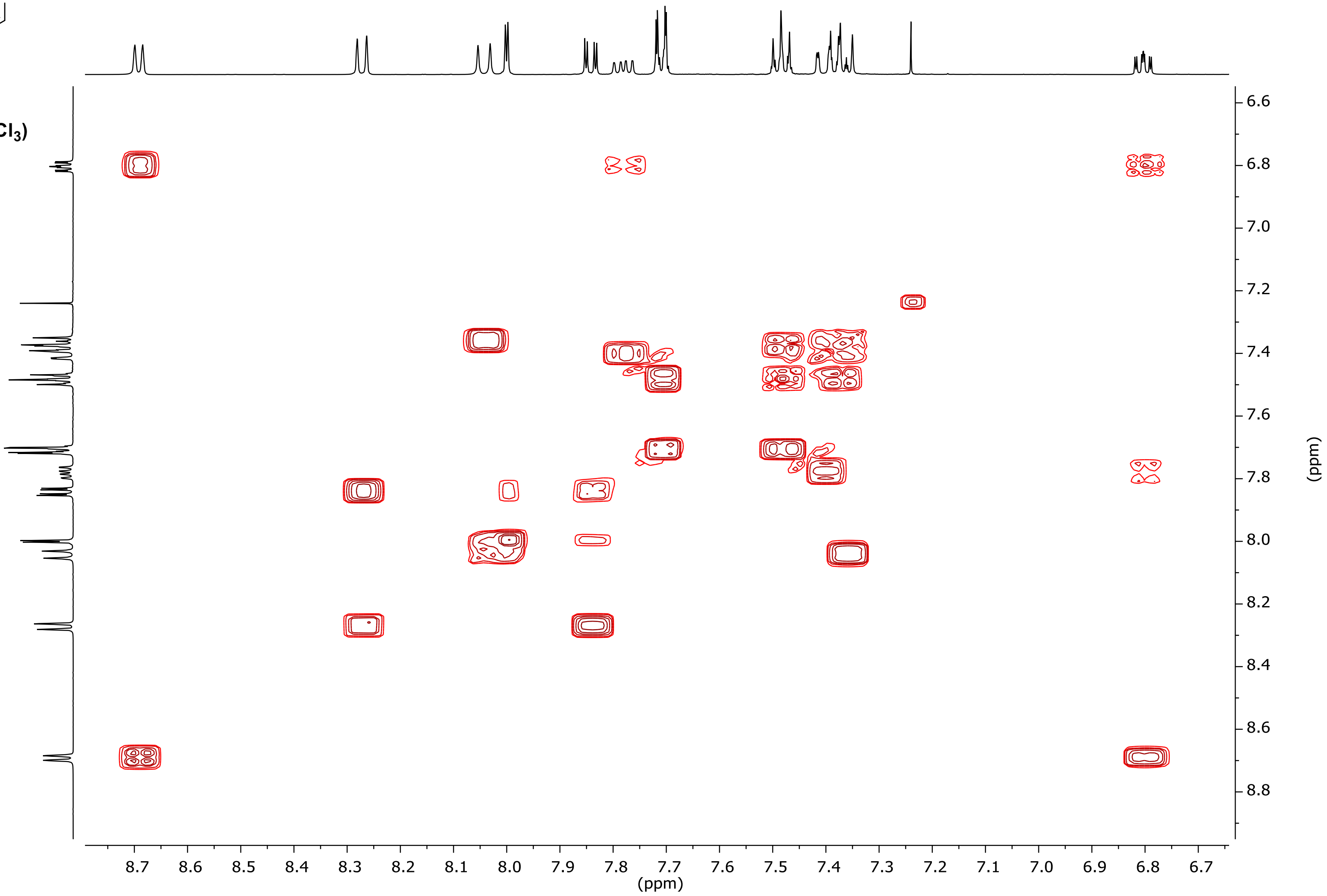

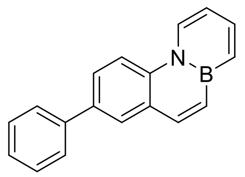

1c

TOCSY-NMR (CDCl$_3$)

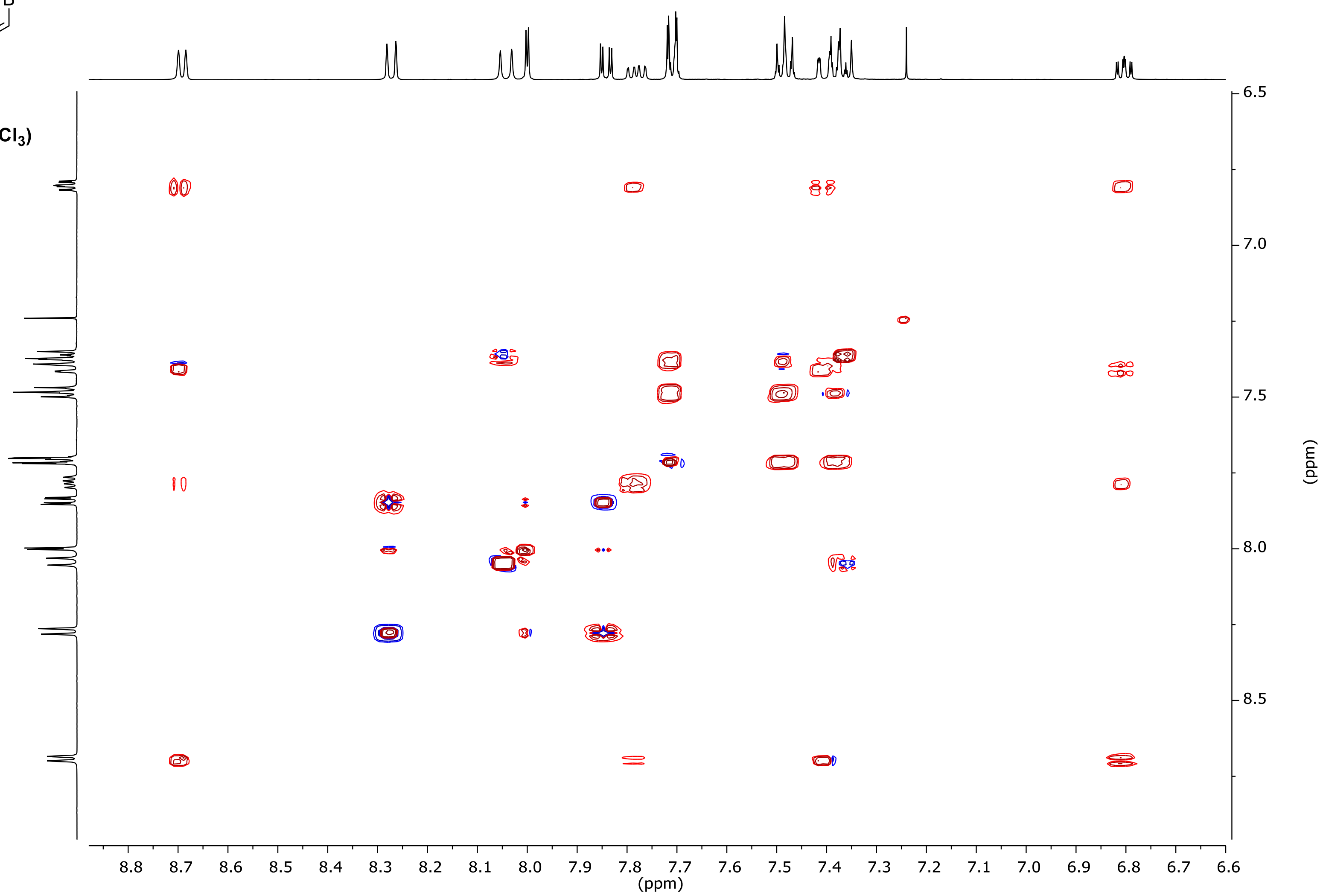

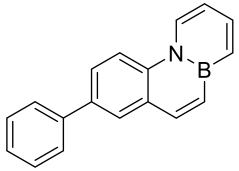

1c

gHSQC-NMR ($CDCl_3$)

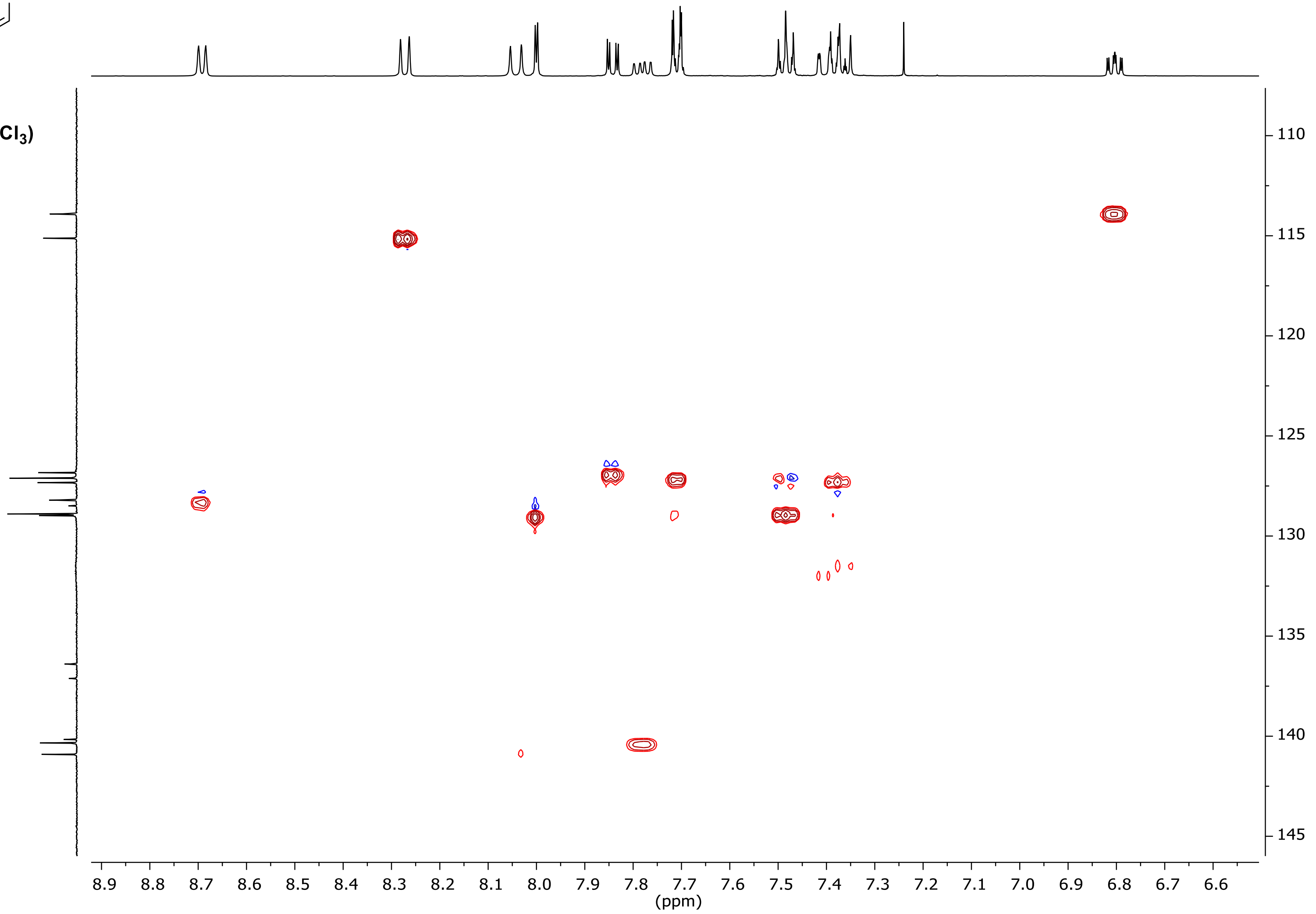

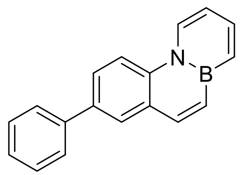

1c

gHMBC-NMR ($CDCl_3$)

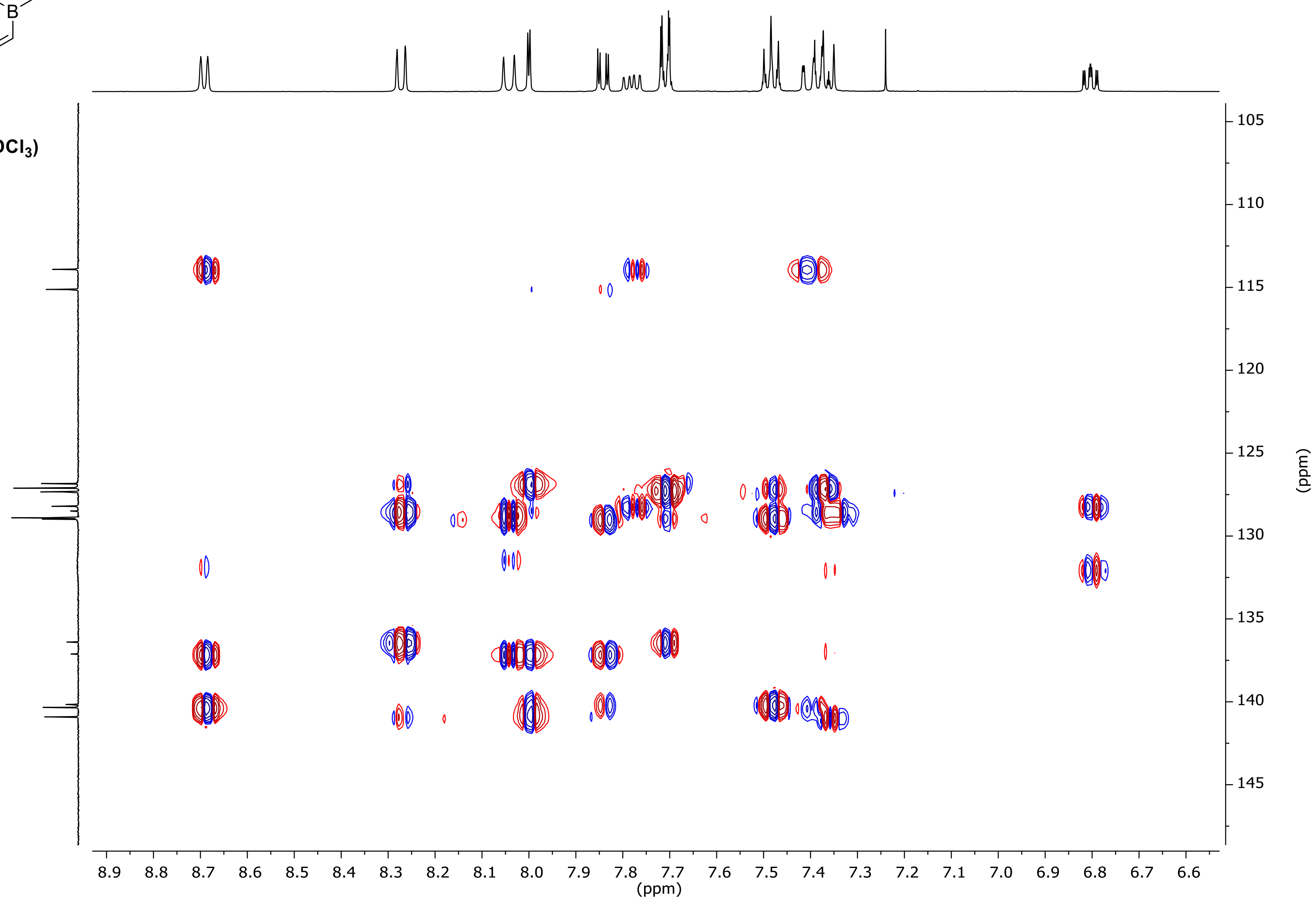

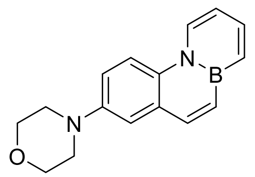

1d

^{1}H-NMR (500 MHz, $CDCl_3$)

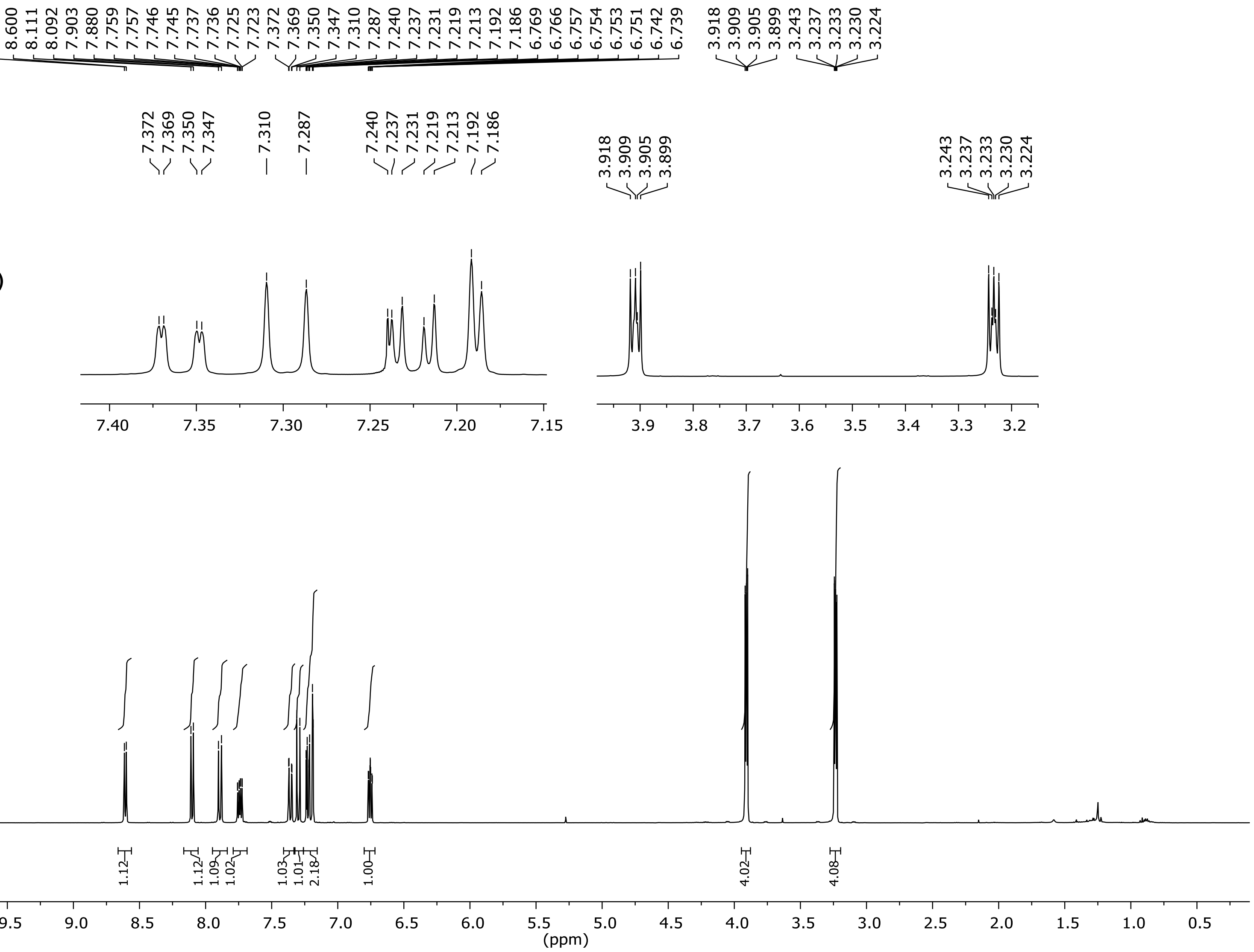

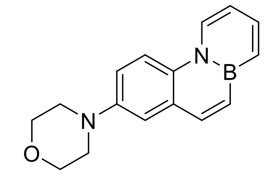

1d

^{13}C-NMR (125 MHz, $CDCl_3$)

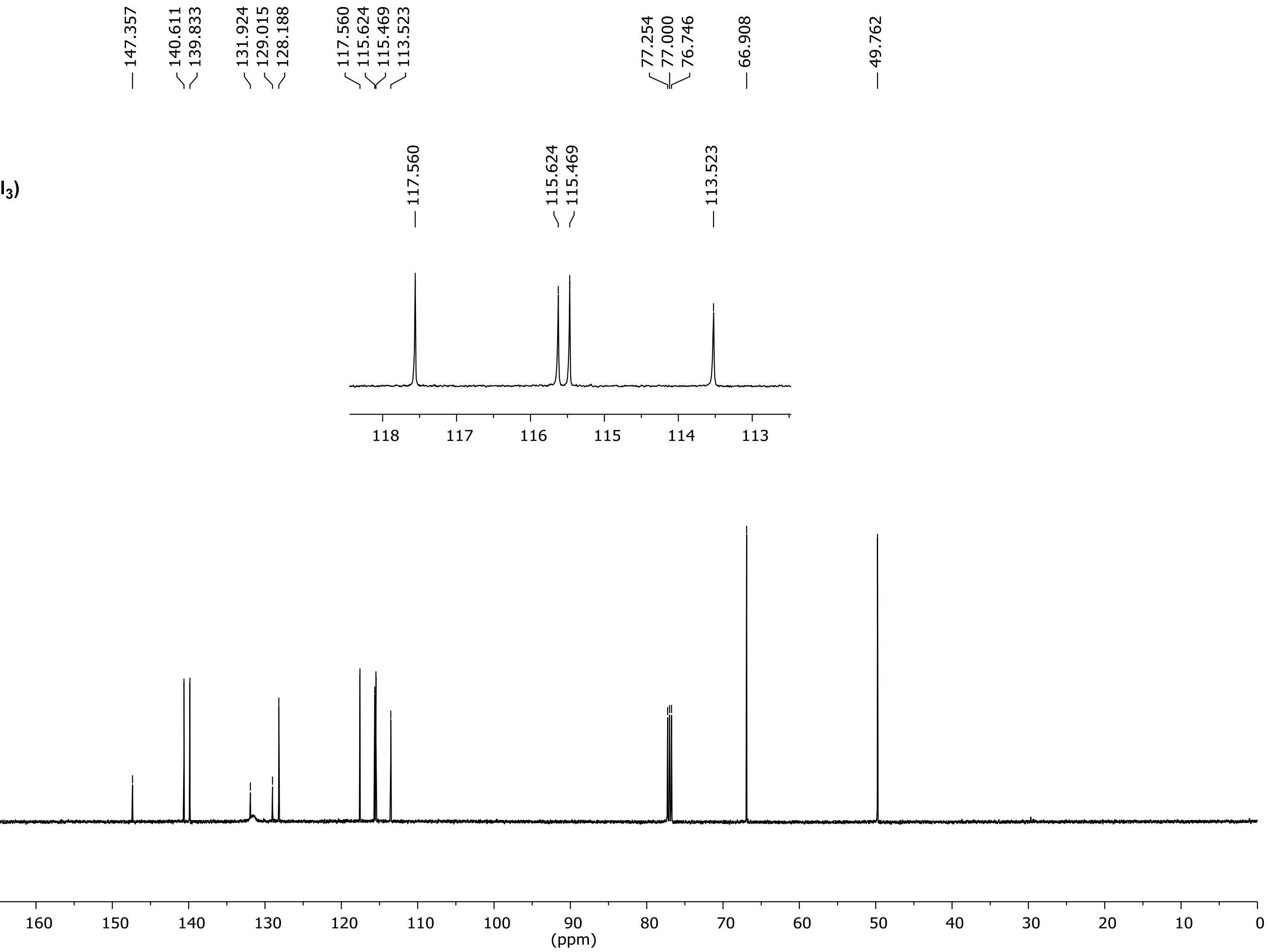

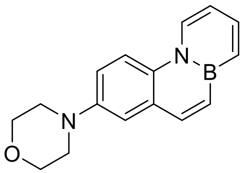

1d

^{11}B-NMR (160 MHz, $CDCl_3$)

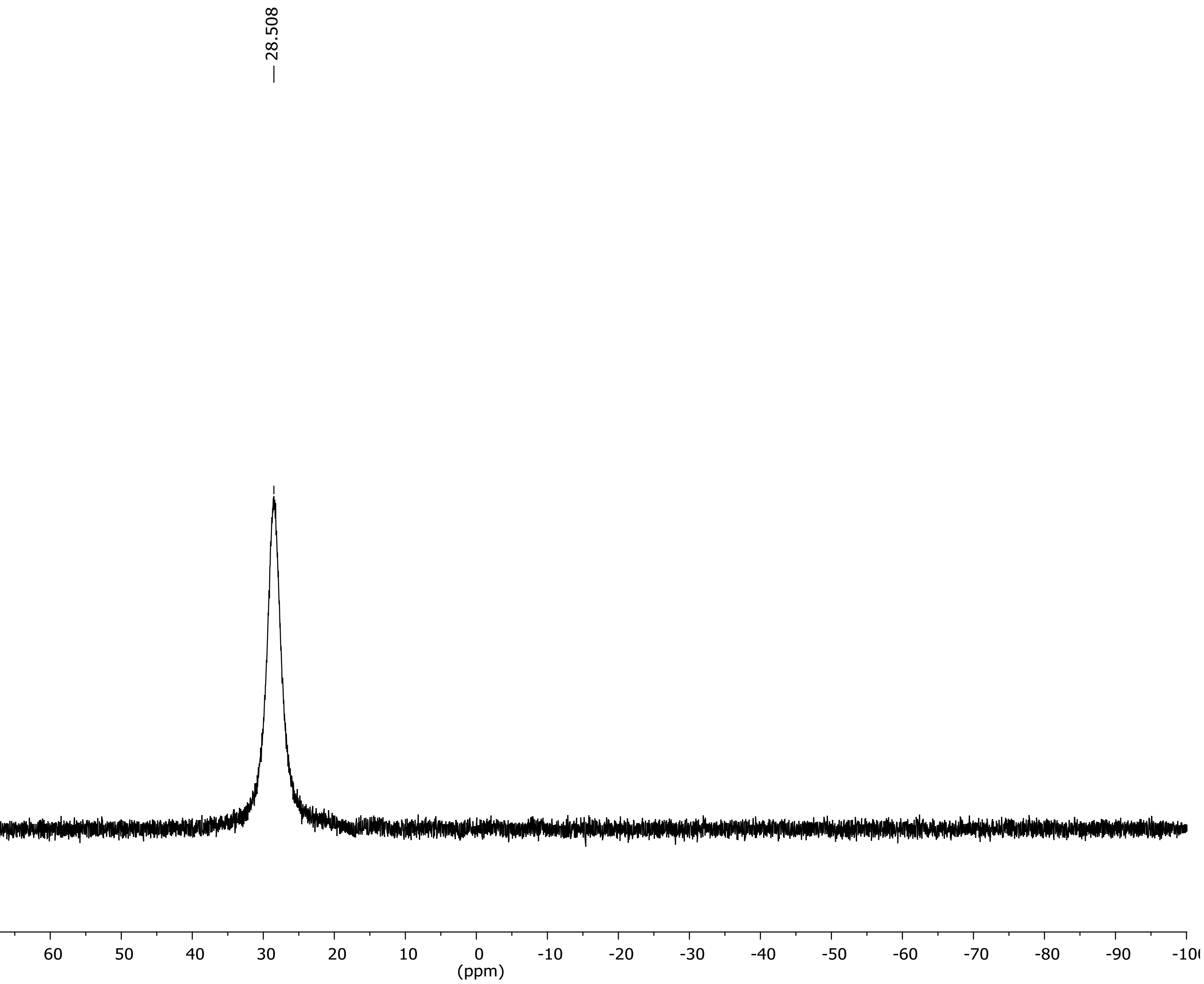

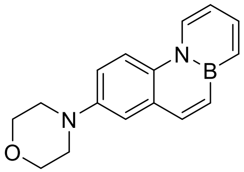

1d

gCOSY-NMR ($CDCl_3$)

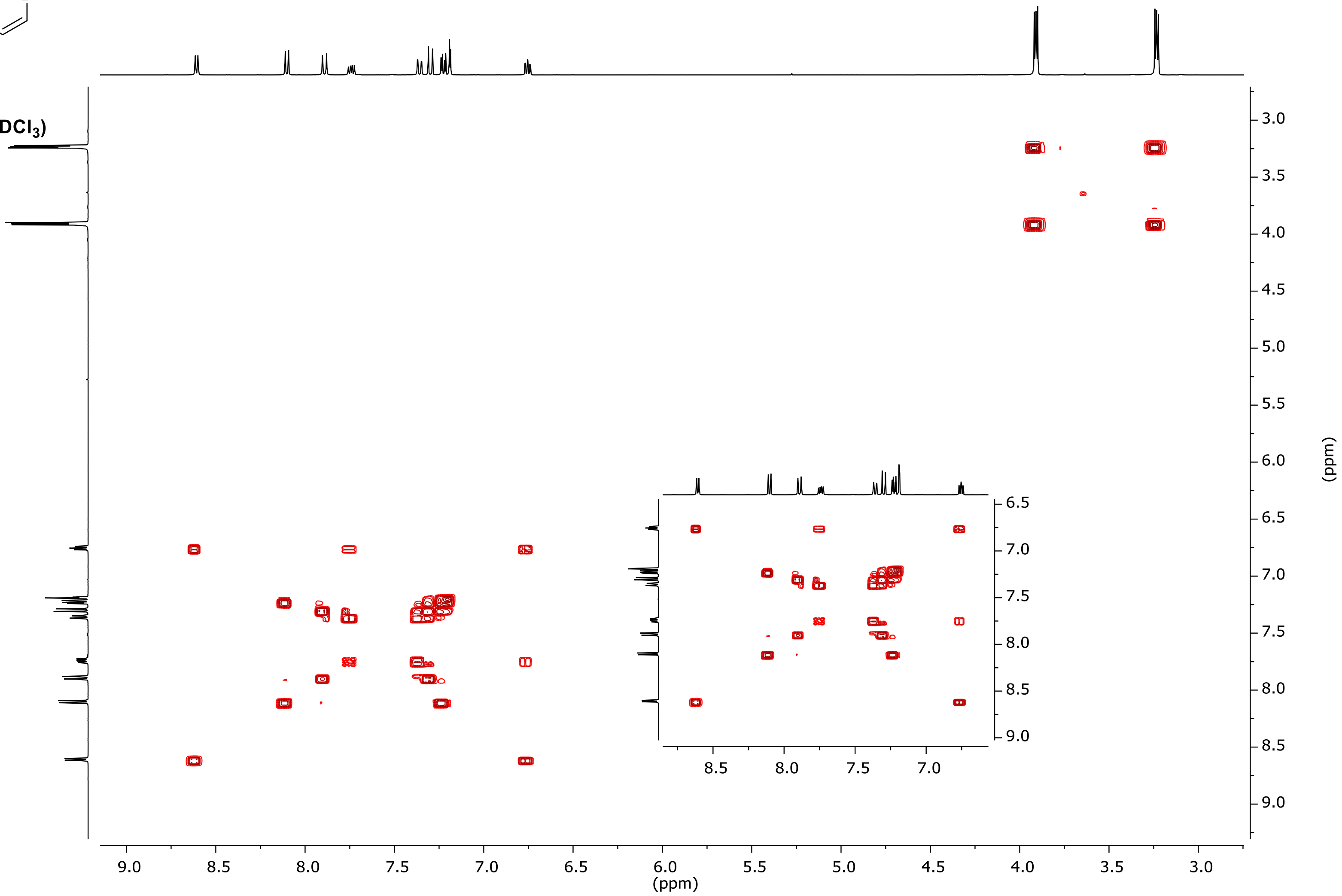

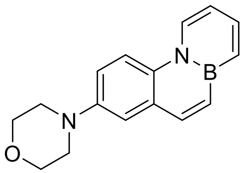

1d

TOCSY-NMR ($CDCl_3$)

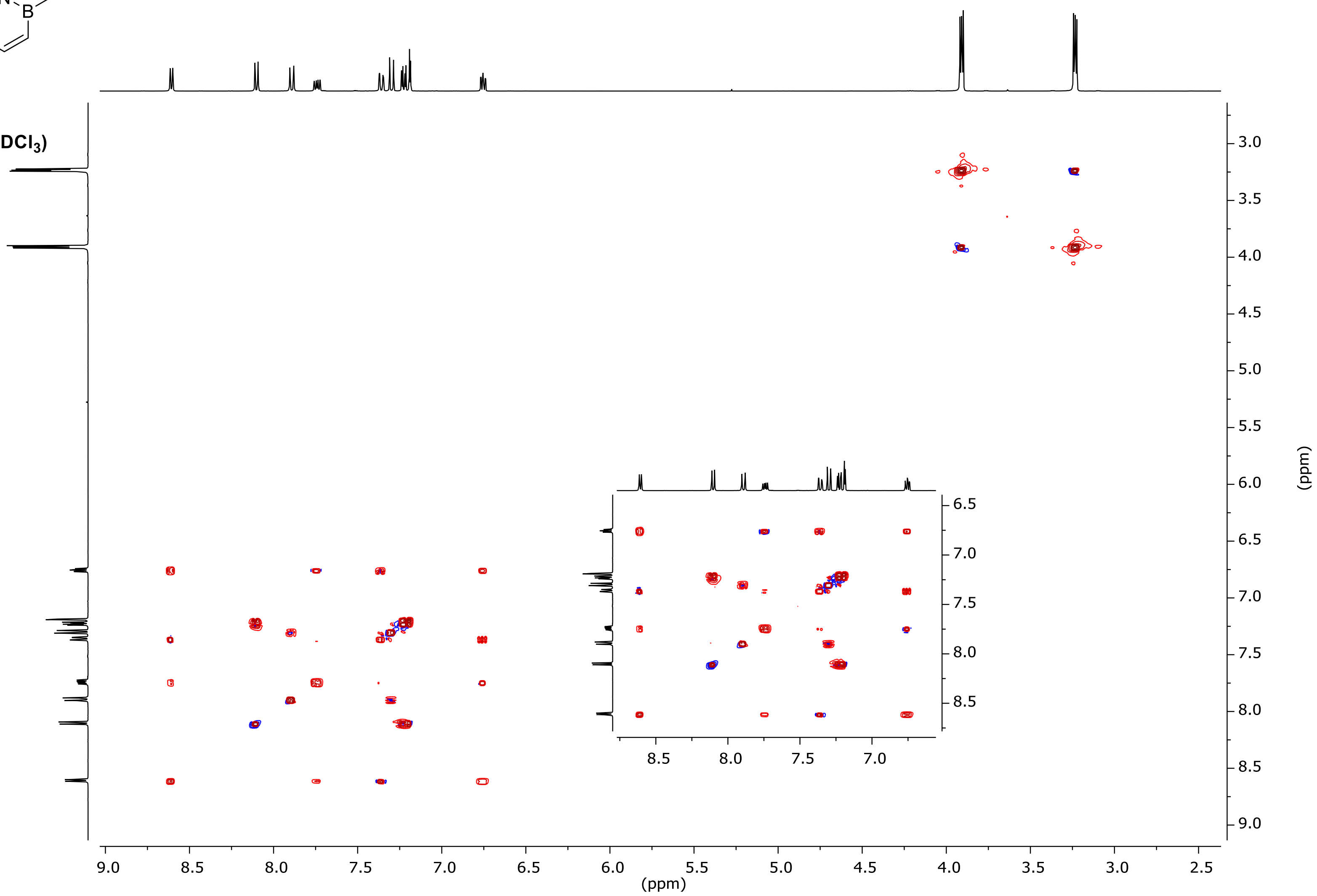

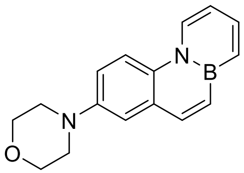

1d

gHSQC-NMR ($CDCl_3$)

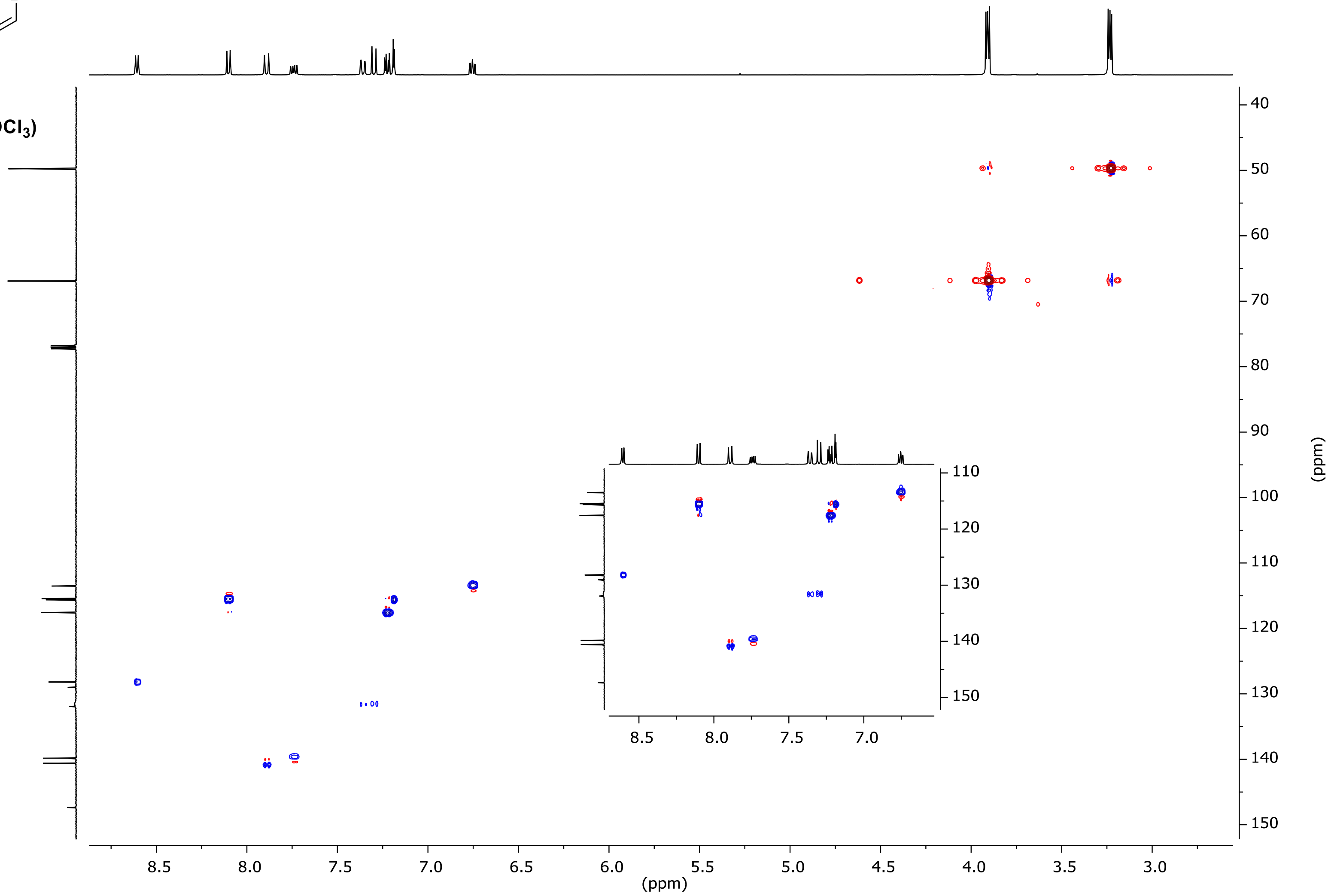

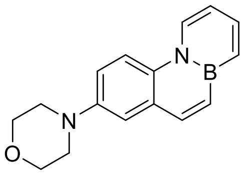

1d

gHMBC-NMR ($CDCl_3$)

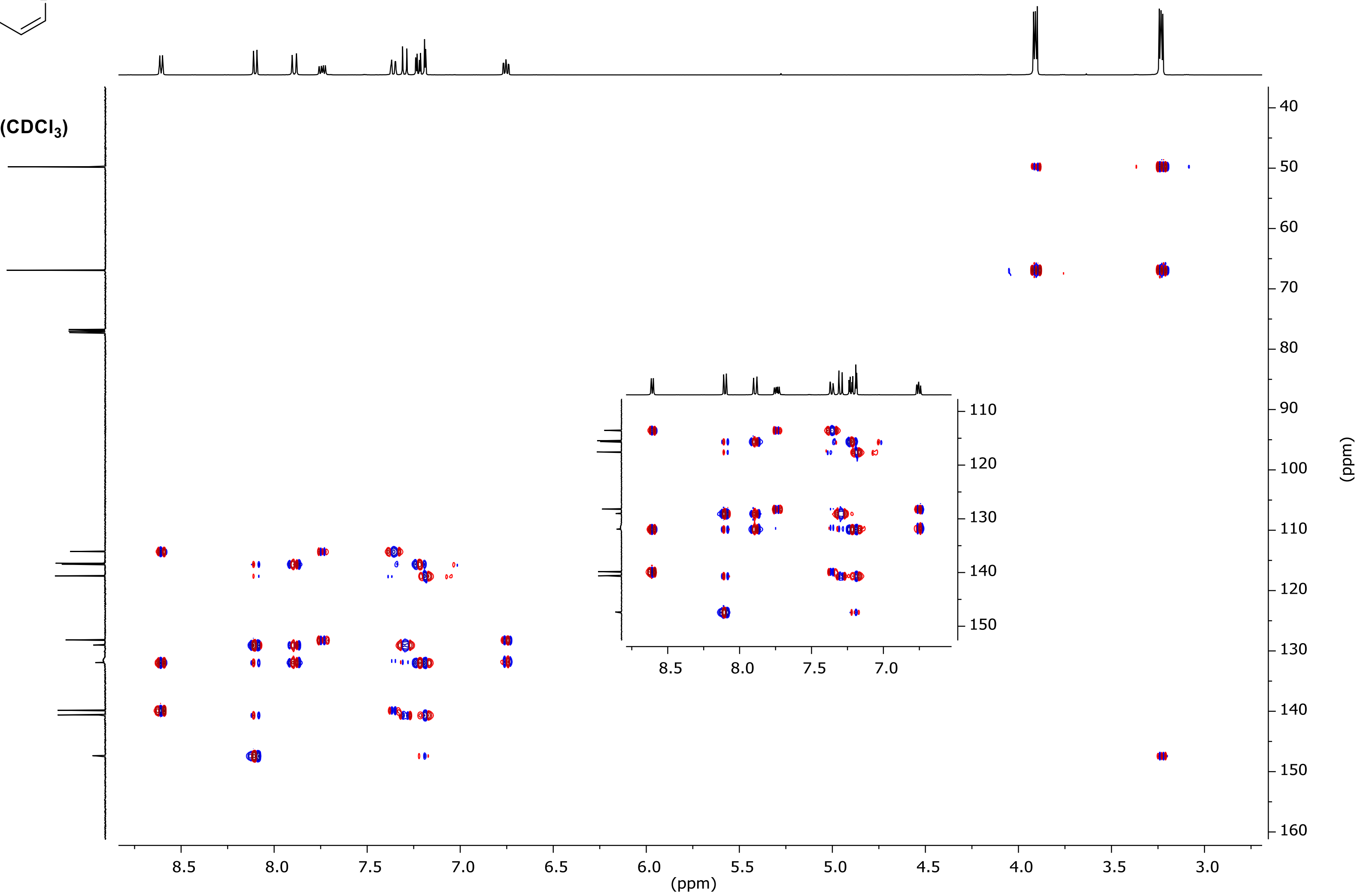

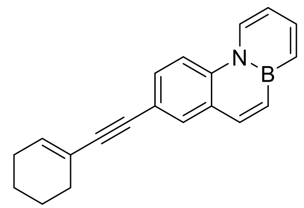

1e

^{1}H-NMR (500 MHz, CDCl$_3$)

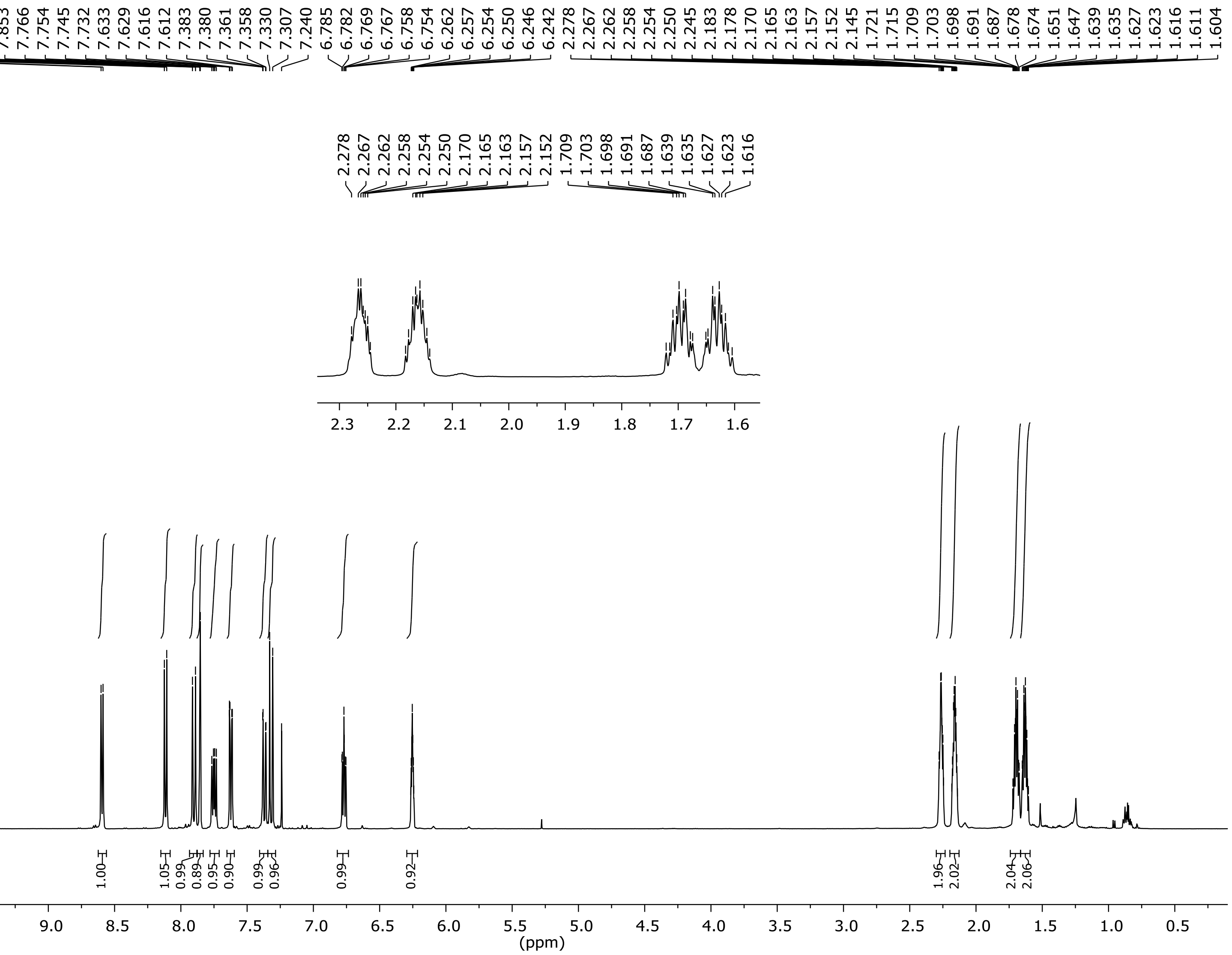

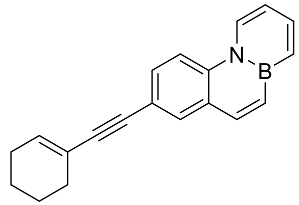

1e

^{13}C-NMR (125 MHz, CDCl$_3$)

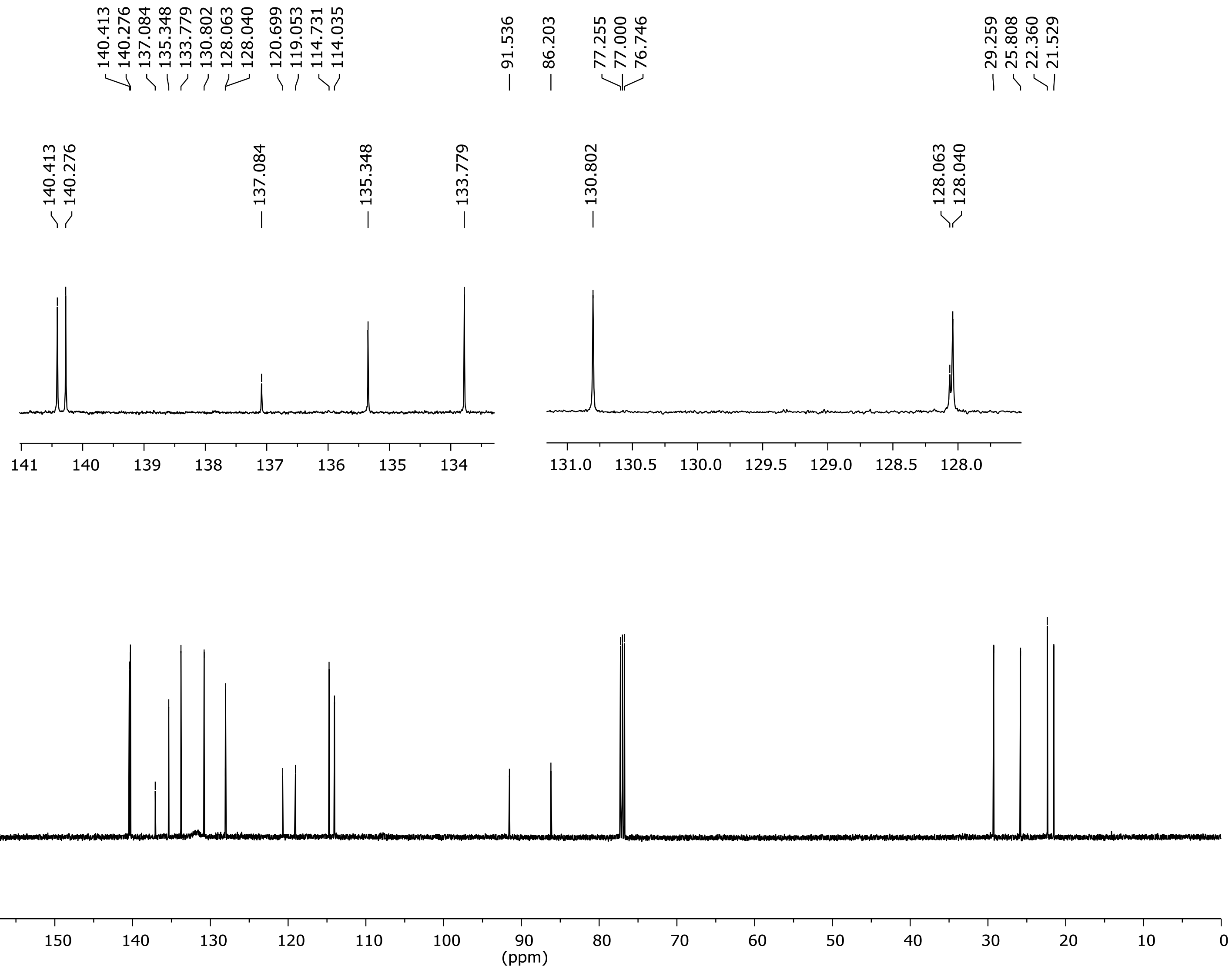

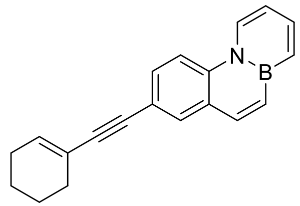

1e

^{11}B-NMR (160 MHz, $CDCl_3$)

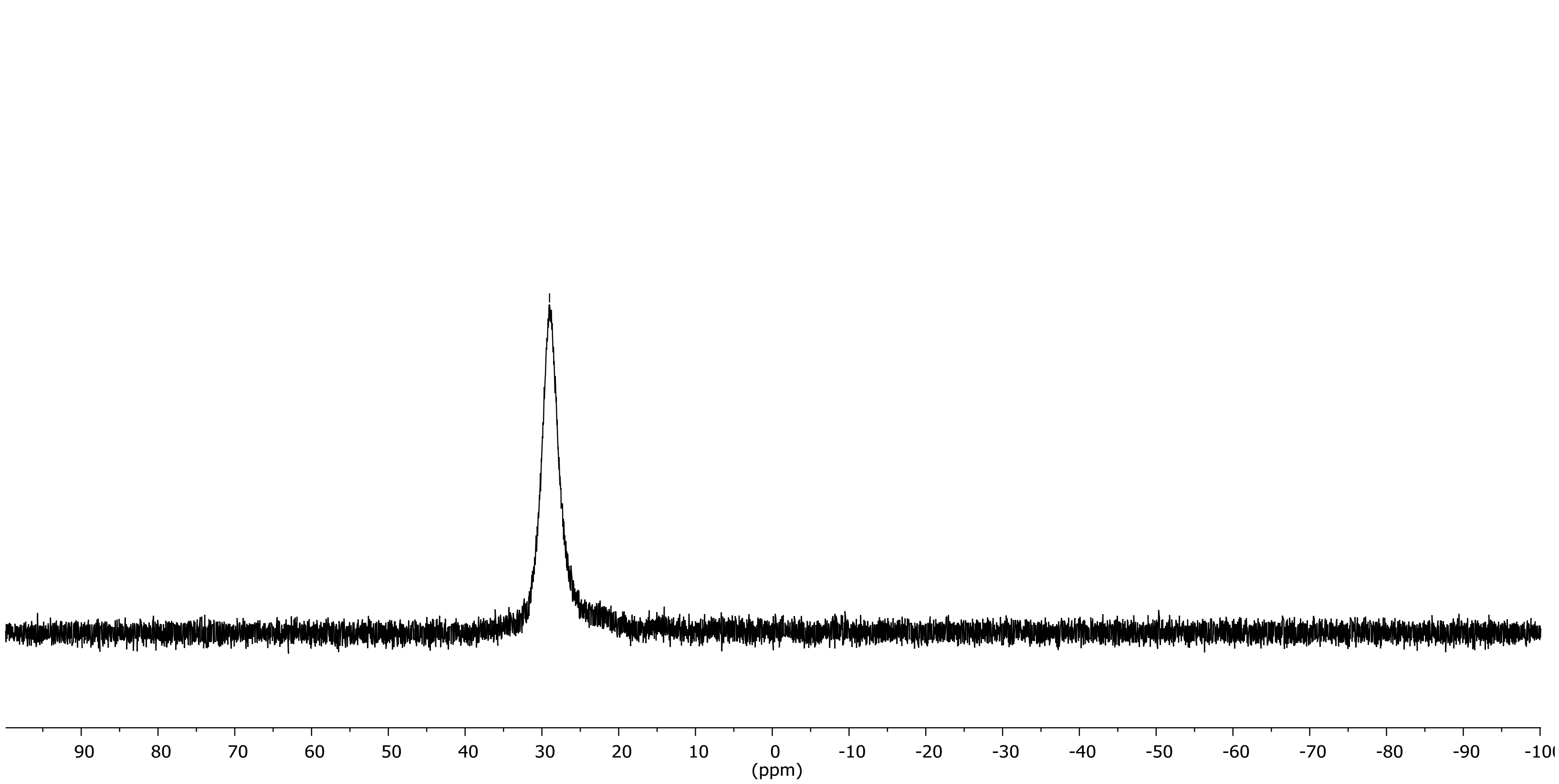

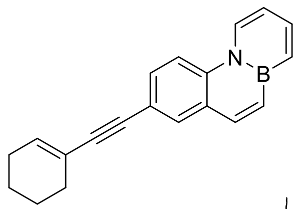

1e

gCOSY-NMR ($CDCl_3$)

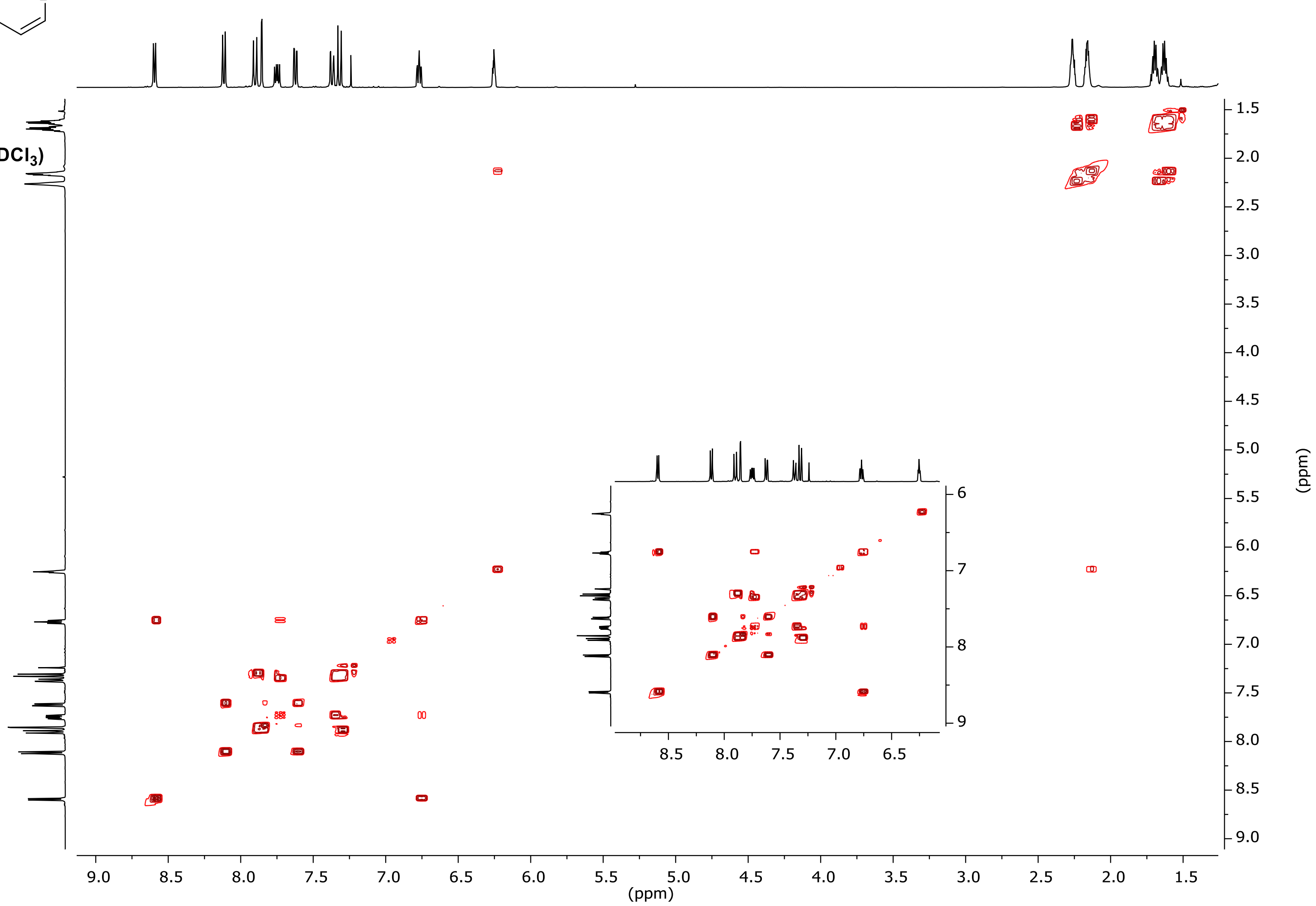

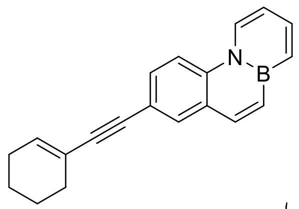

1e

TOCSY-NMR ($CDCl_3$)

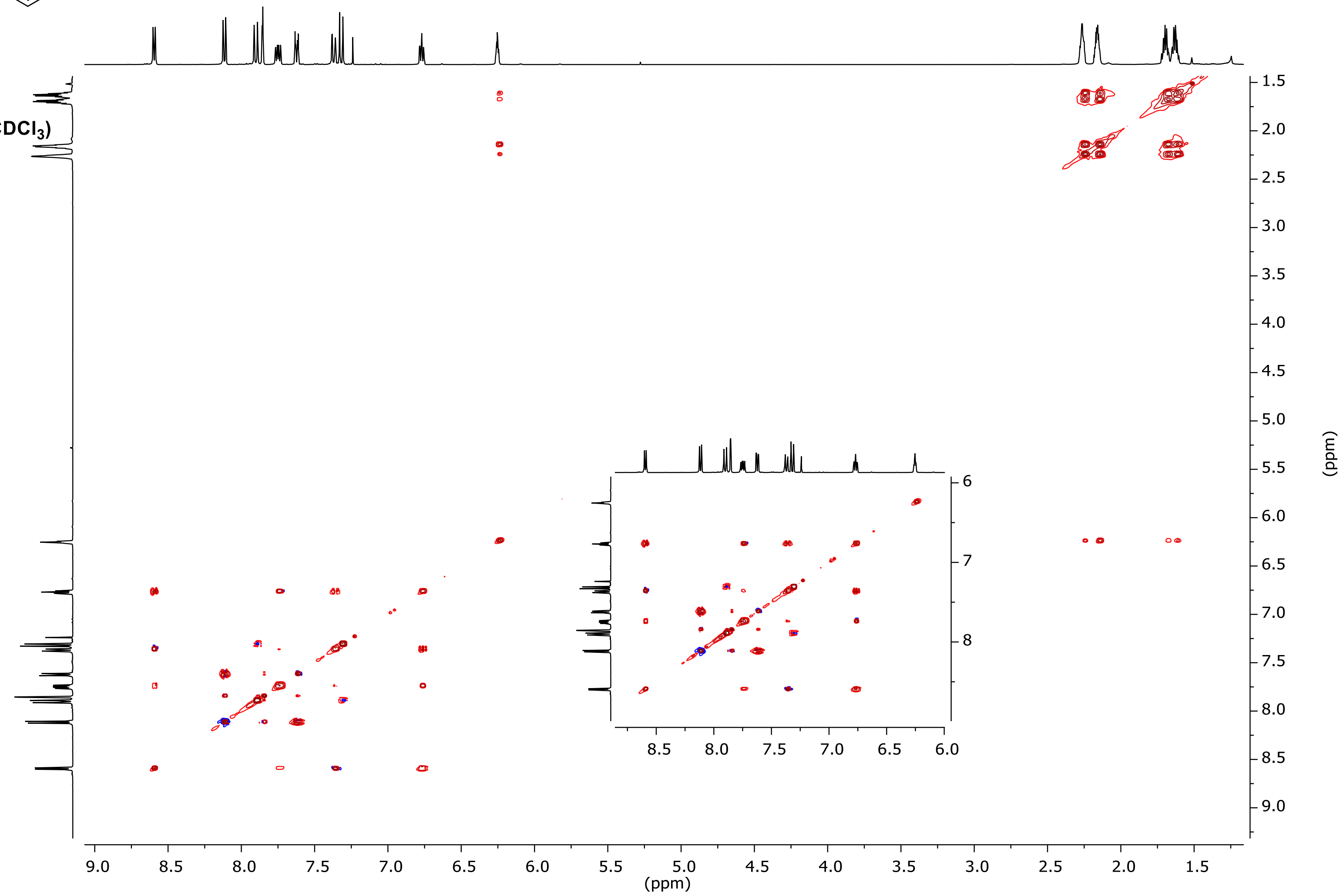

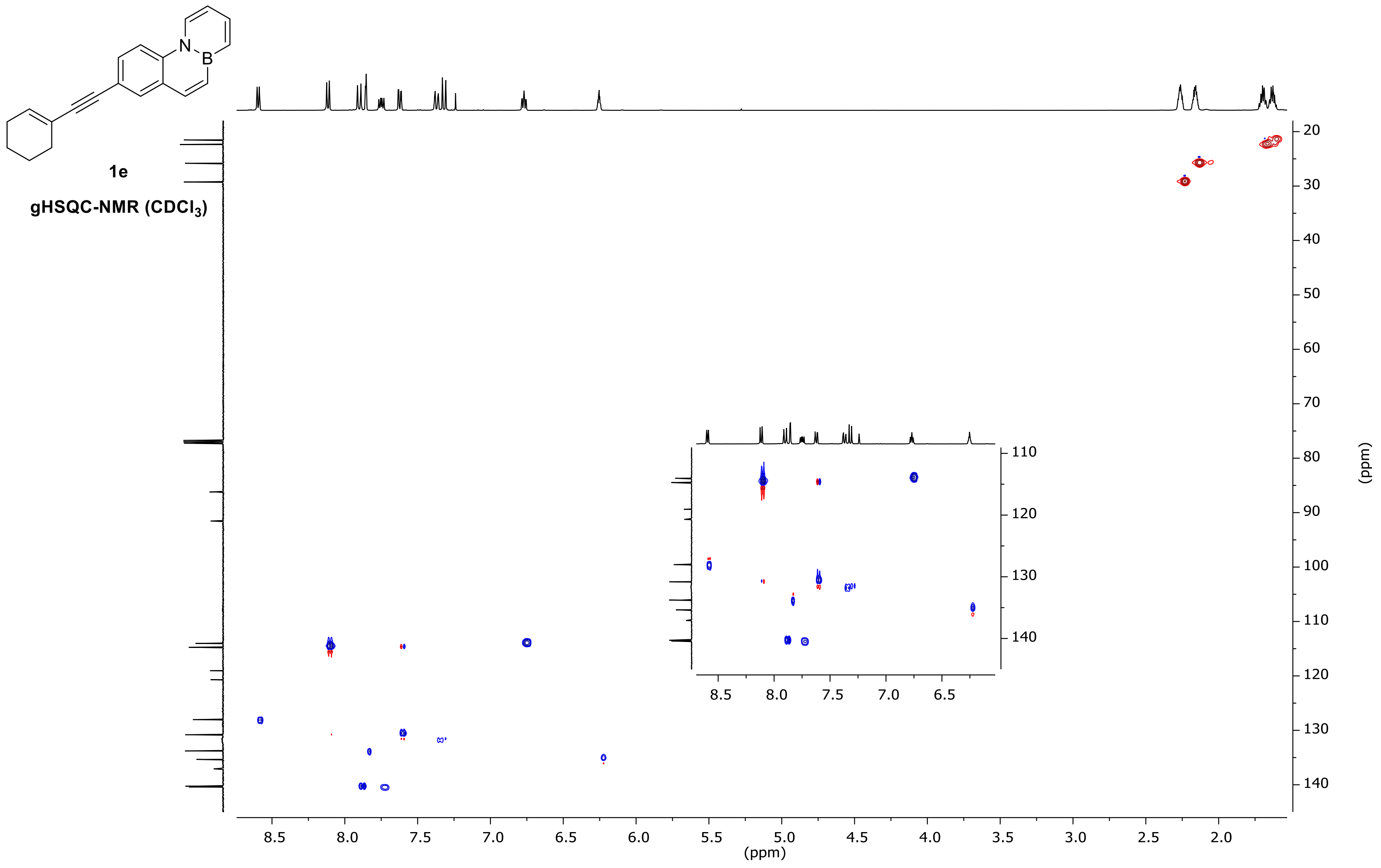
1e
gHSQC-NMR (CDCl3)
(ppm)
(ppm)

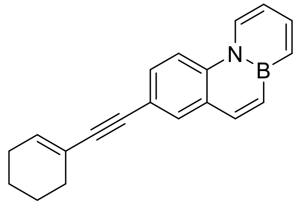

1e

gHMBC-NMR ($CDCl_3$)

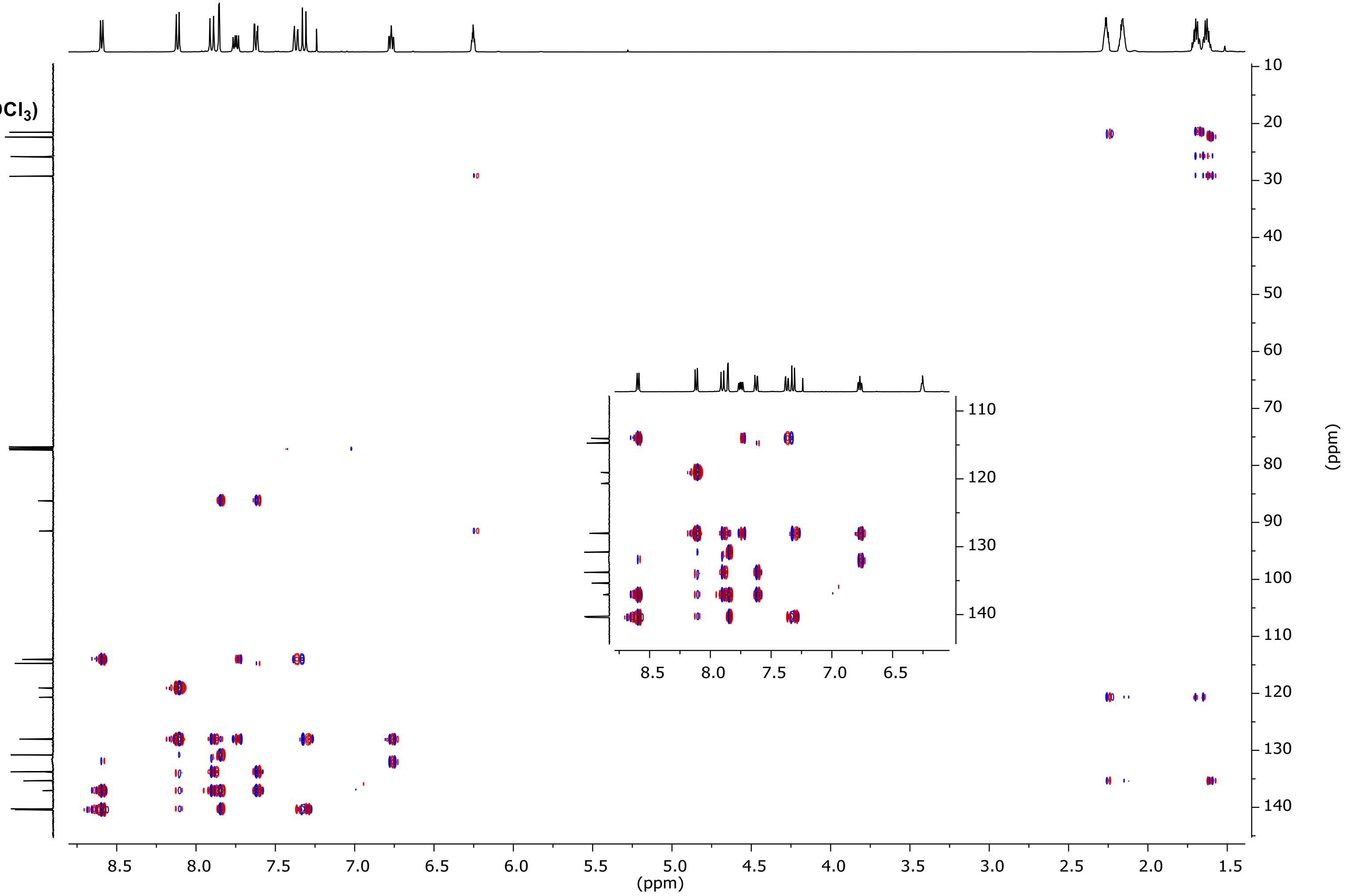

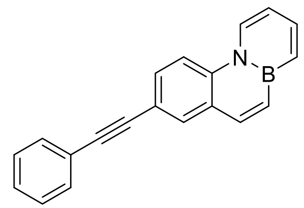

1f

^{1}H-NMR (500 MHz, $CDCl_3$)

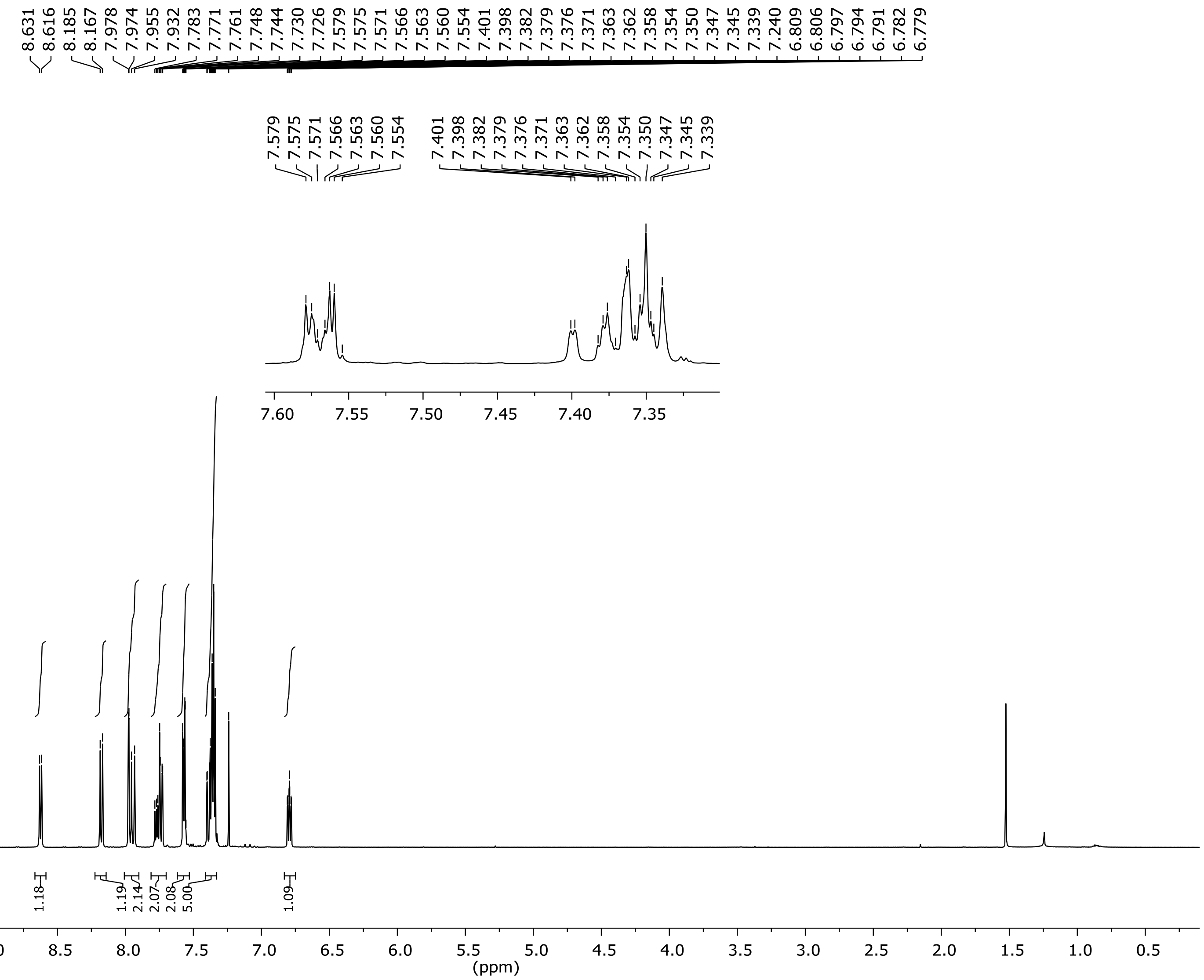

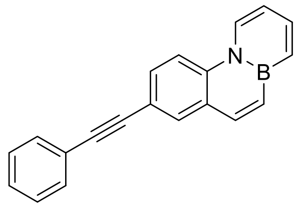

1f

^{13}C-NMR (125 MHz, $CDCl_3$)

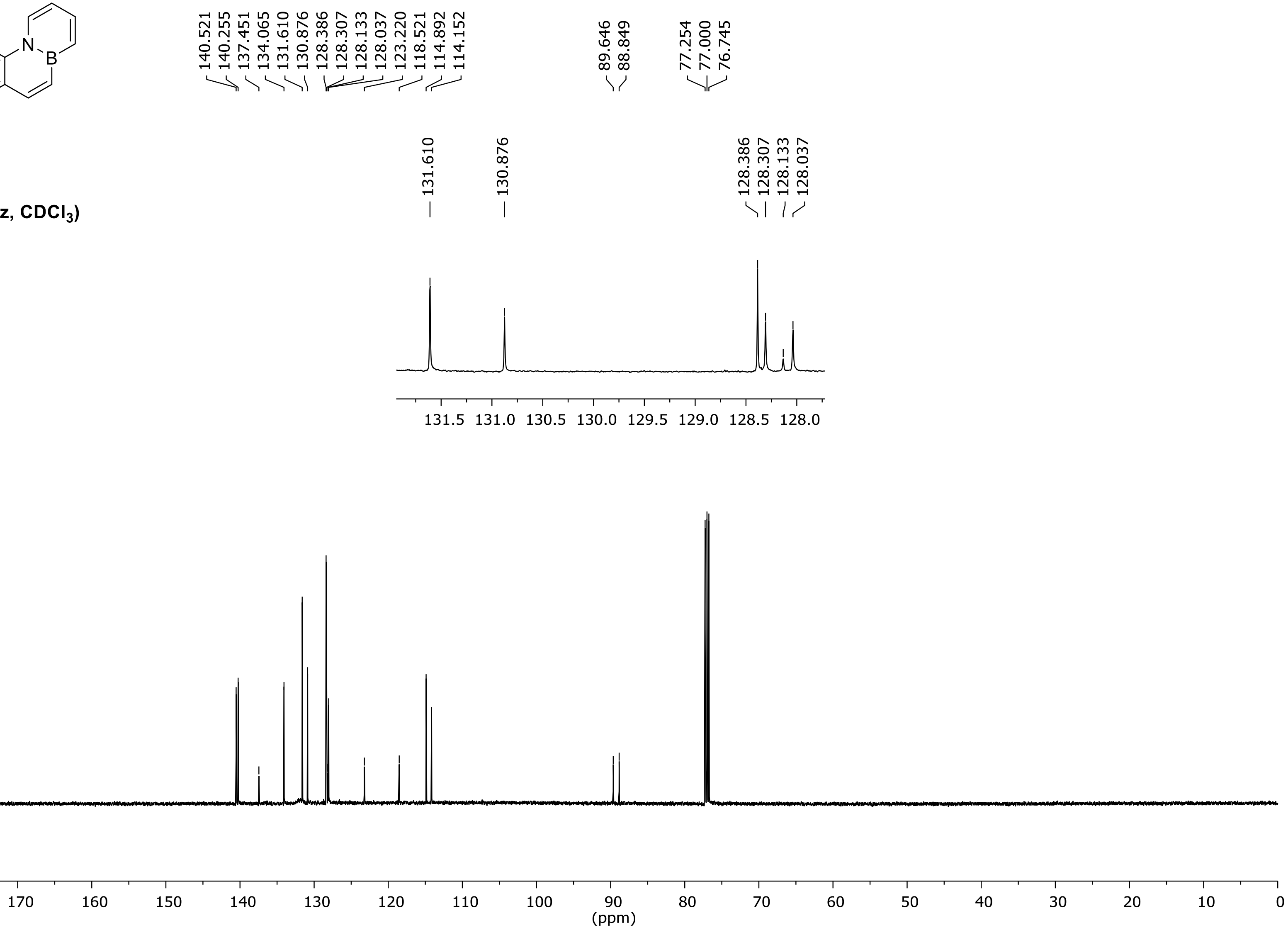

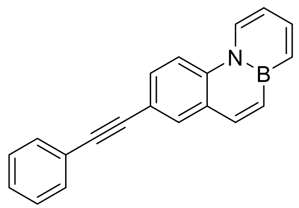

1f

^{11}B-NMR (160 MHz, $CDCl_3$)

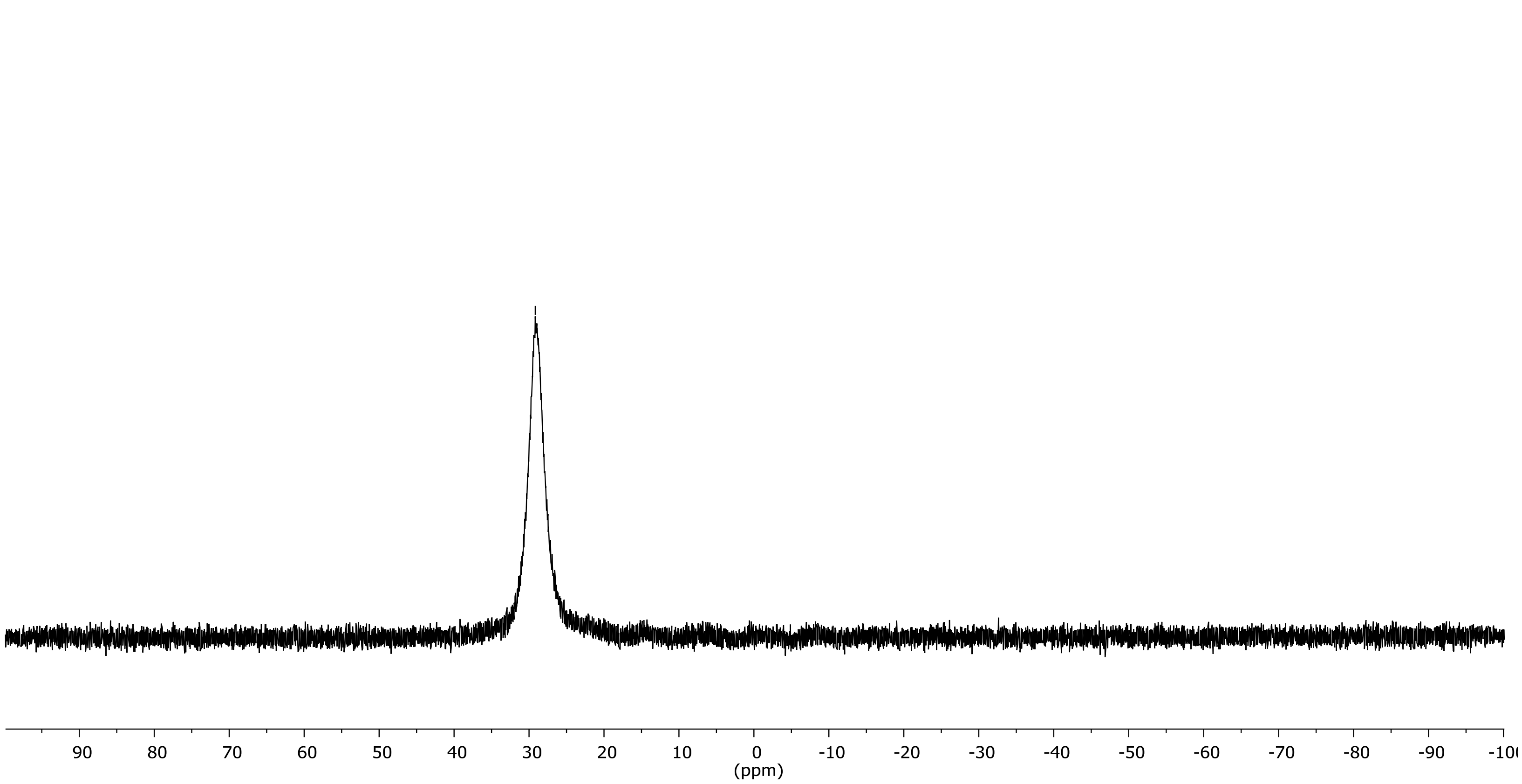

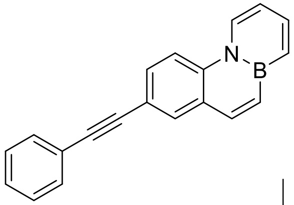

1f

gCOSY-NMR ($CDCl_3$)

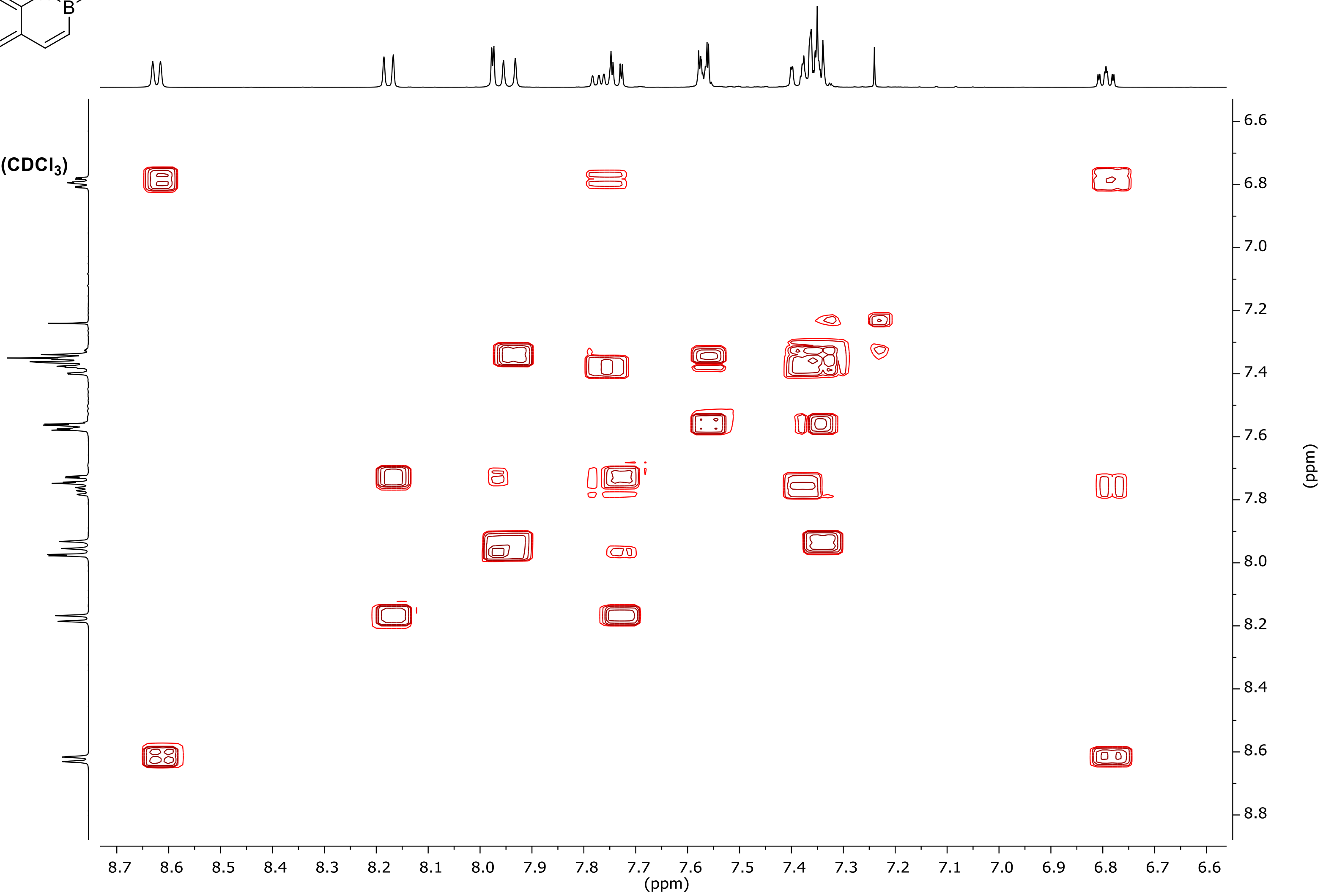

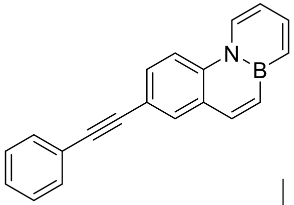

1f

TOCSY-NMR ($CDCl_3$)

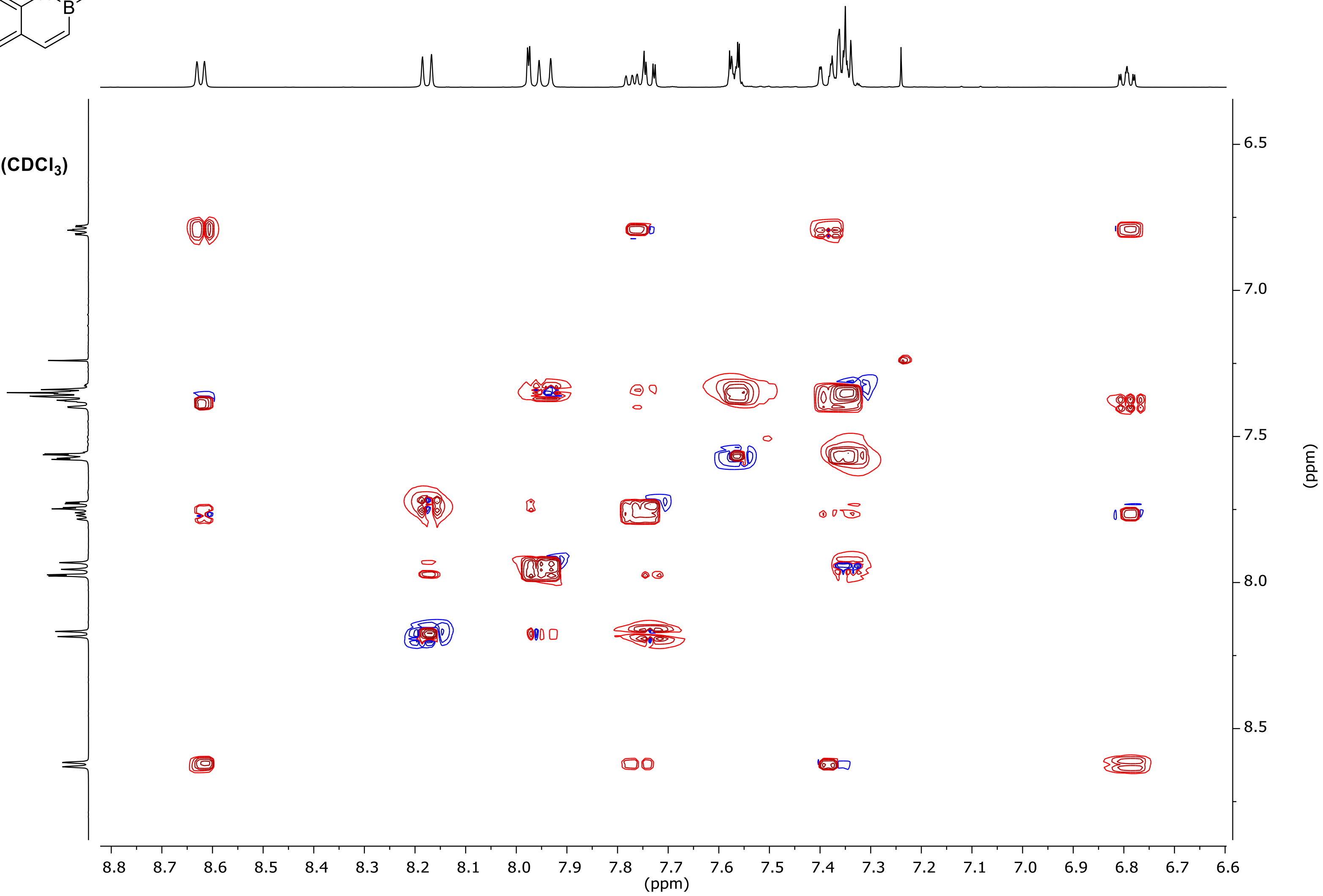

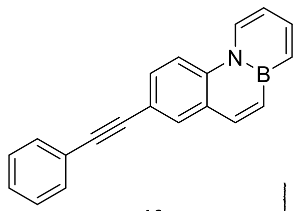

1f

gHSQC-NMR (CDCl$_3$)

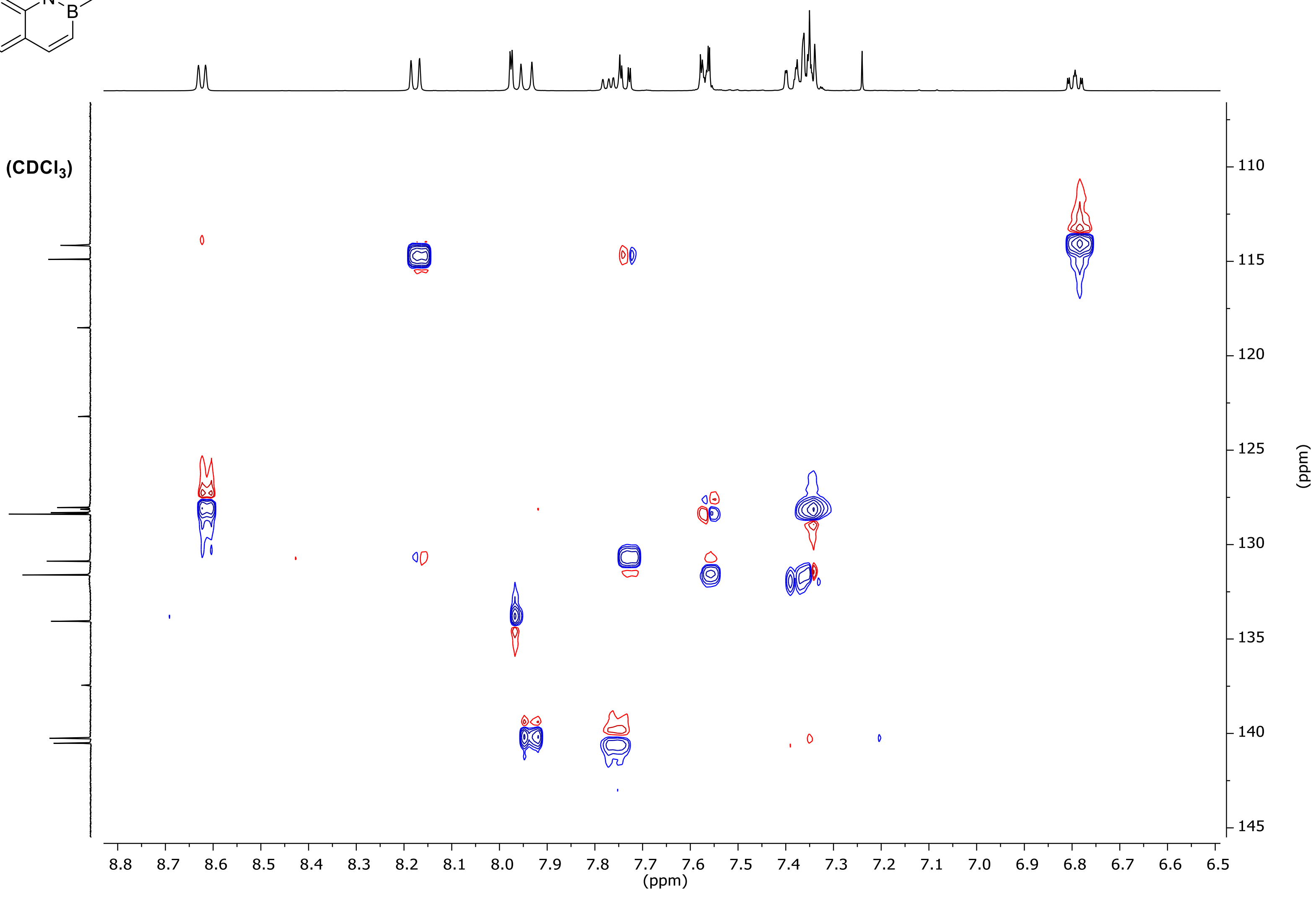

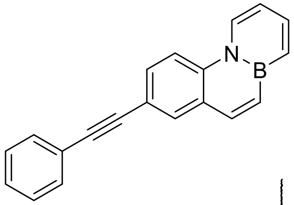

1f

gHMBC-NMR ($CDCl_3$)

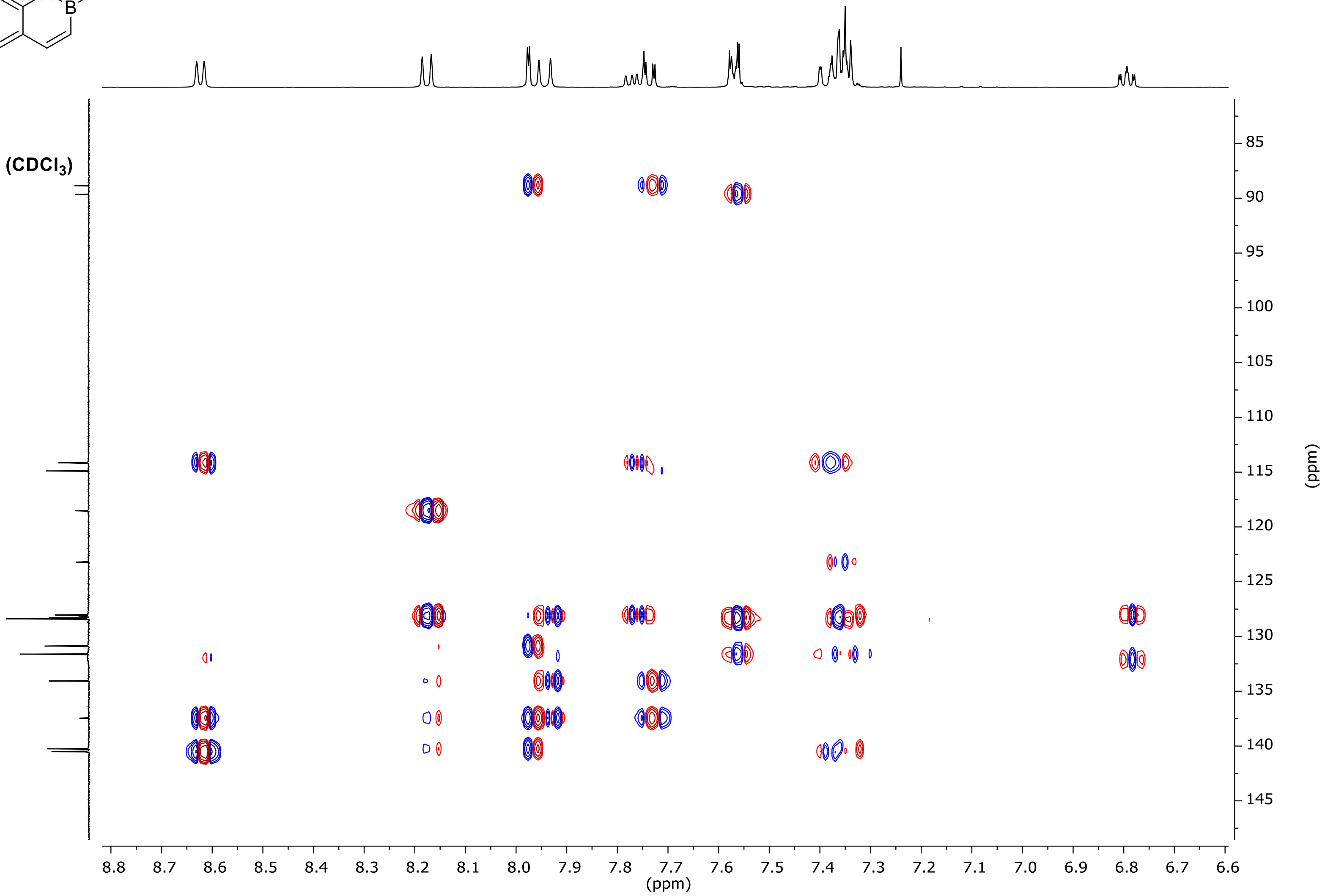

Supplement: File 1 — Experimental details and NMR spectra for all new compounds. [file Beilstein_J_Org_Chem-15-1257-s001.pdf]
